# Substrate Specificity and Kinetic Mechanism of the NAD^+^‑Dependent Deacylase from *Mycobacterium tuberculosis*, Rv1151c (*Mt*-Sirt)

**DOI:** 10.1021/acs.biochem.6c00374

**Published:** 2026-07-13

**Authors:** Drake M. Mellott, Jiyun Zhu, Hudson Fulcher, Thomas D. Meek

**Affiliations:** Department of Biochemistry and Biophysics, 14736 Texas A&M University, College Station, Texas 77843, United States

## Abstract

The pathogen *Mycobacterium tuberculosis* (*Mtb*) relies on extensive metabolic remodeling and post-translational regulation of its proteins to retain persistence in its host, yet these biochemical functions remain largely uncharacterized, including the actions of lysine deacylases. Here, we characterized Rv1151c, the sole annotated NAD^+^-dependent deacylase in *Mtb*, which we designate *Mt*-Sirt. Using synthetic substrates, peptide libraries, and mutational analysis, we showed that *Mt*-Sirt exhibits strong selectivity for peptide substrates containing N^ε^-succinyl-lysine and long-chain fatty-acyl-lysine groups. Substitution of conserved active-site residues (Y53F and R56M) confirmed their roles in substrate recognition and acyl-group discrimination. Inhibition studies revealed that nicotinamide, NADH, ADP-ribose, and mechanism-based thioamide/thiourea analogs inhibited *Mt*-Sirt activity with low-micromolar to nanomolar potency. Initial-velocity, product-inhibition, and dead-end inhibition analyses confirmed that *Mt*-Sirt operates by an equilibrium-ordered kinetic mechanism in which NAD^+^ binds first, followed by the N^ε^-acylated peptide substrate. We further demonstrated that chemically succinylated *Mtb* isocitrate lyase 1 (ICL1), a key enzyme in *Mtb* metabolism, was a substrate of *Mt*-Sirt *in vitro*. Lastly, metabolic labeling with tetradec-13-ynoic acid (Alk-12) revealed widespread lysine and N-terminal fatty acylation in *Mtb* cells, suggesting that *Mt*-Sirt may regulate previously unrecognized fatty-acyl PTMs *in vivo*. Together, these findings provide a kinetic and substrate-specificity analysis of *Mt*-Sirt, and highlight its potential role in controlling reversible lysine acylation in *Mtb*.

## Graphical abstract

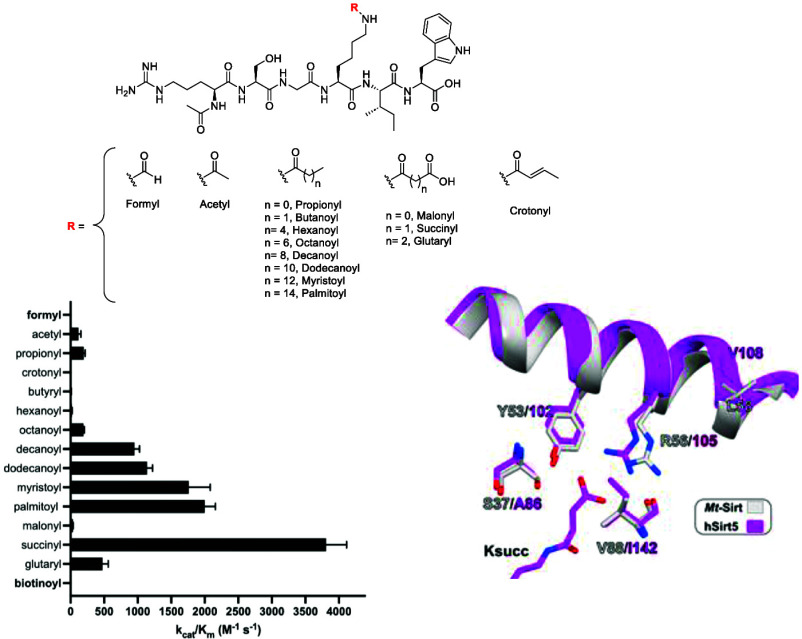

## Introduction

The post-translational modification (PTM) of the ε-amino groups of lysine residues serves as a rapid chemical switch by which organisms can modulate the function of proteins.^1,2^ Generally, these modifications occur on lysine residues of a protein either by the action of an acyl transferase, a methyl transferase, or nonenzymatic acylation with a reactive metabolite such as succinyl-CoA.^3^ Removal of these acyl groups is primarily catalyzed by two enzyme families: NAD^+^-dependent sirtuin deacylases and metal-dependent histone deacetylases.^4^ In addition to these classes of enzymes, activity-based protein profiling identified an orphan serine hydrolase that catalyzes the removal of acyl groups from various lysines from peptides and proteins, suggesting that additional deacylase families remain uncharacterized.^5^

Silent regulators of information (SIR) genes function as “erasers” of the ε-acyl-lysine residues on proteins. These enzymes, conserved from prokaryotes to eukaryotes, employ NAD^+^ as a cosubstrate to catalyze the hydrolysis of an amide bond of acyl-modified lysine residues on proteins, in which one molecule of nicotinamide is released per deacylation event, and the acyl group is transferred from lysine to 2′-*O*-acyl-ADP-ribose. The catalytic mechanism for sirtuins has been elucidated (Figure 1): (a) the carbonyl oxygen of the lysine acyl-group attacks carbon-1′ of NAD^+^ to displace nicotinamide; (b) the resulting 1′-*O*-alkylimidate then cyclizes to form a 1′-2′-cyclic intermediate which, (c) after expulsion of the lysine group, (d) undergoes hydrolysis to afford the 2′-*O*-acyl adenine dinucleotide ribose product (Figure 1).^4–7^ There are seven known human sirtuins (SIRT1–7) that mediate the removal of a large array of acyl groups.^8^ These enzymes, in addition to their differing substrate specificities, have been shown to play a role in diverse biological processes such as aging, gene regulation, energy metabolism, signal transduction, and clinically relevant pathologies such as cancer, cardiovascular diseases, neurodegeneration, and metabolic diseases.^9^

**Figure 1:**
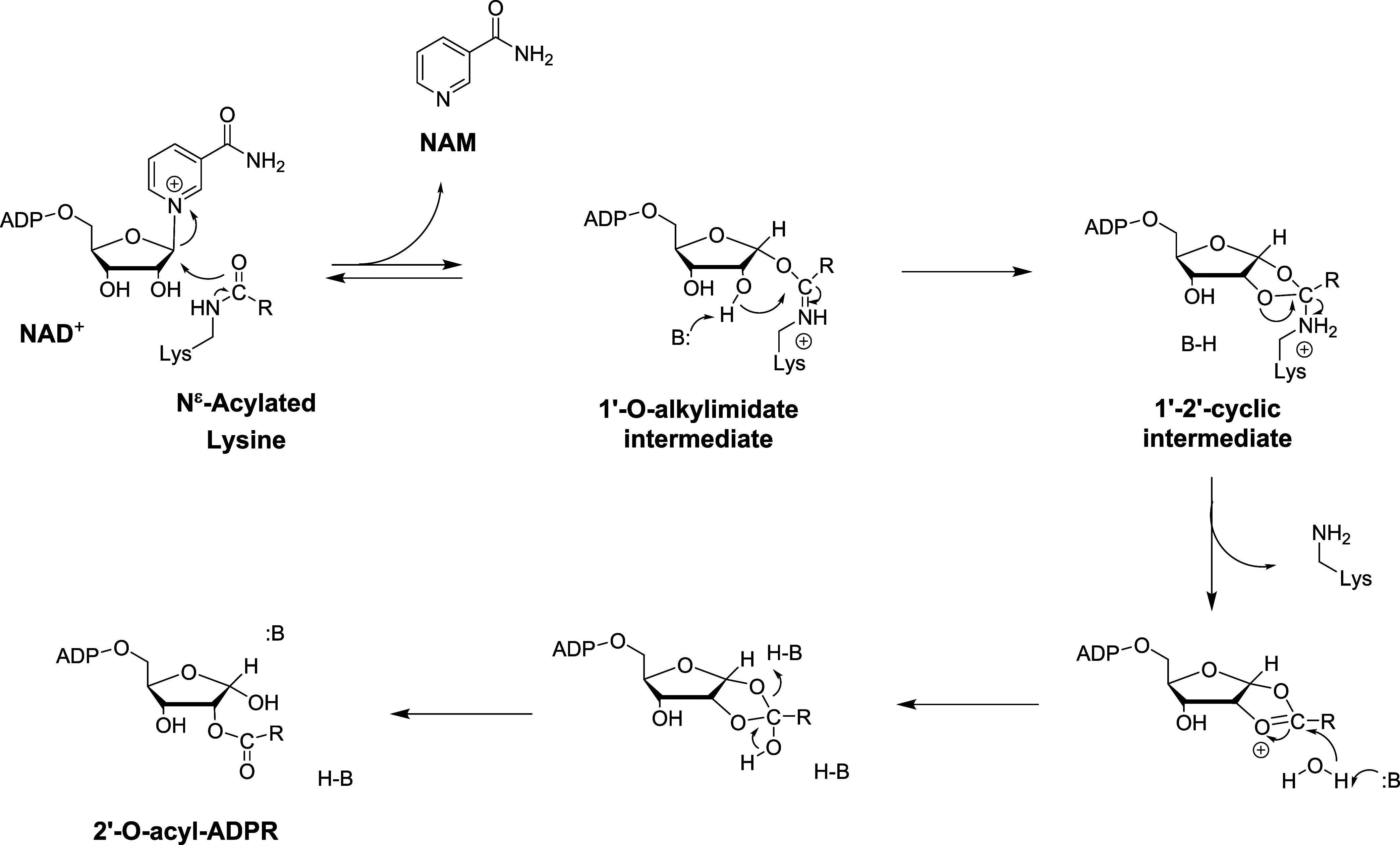
Putative, composite chemical mechanism of NAD^+^-dependent lysine deacylase as derived from previous studies.^6–9^

Of the panoply of ε-aminoacyl-lysyl PTMs known, among the most studied is the N^ε^-acetylation of lysine residues. This PTM has been shown to be present in organisms ranging from prokaryotes to eukaryotes, and also exerts a role in biological functions ranging from metabolism, transcription, DNA repair, among others.^10^ Advances in mass spectrometry have allowed the elucidation of many new and structurally diverse types of lysine PTMs in which the acyl substituents range from carboxylates (glutaryl, malonyl, and succinyl), fatty acid chains of variable lengths (propionyl, butyryl, crotonyl, myristoyl, palmitoyl, etc.), and even cofactors such as biotin and lipoamide.^11^

Many of these PTMs have yet to be characterized with respect to the role they may play on key proteins and the ability of these diverse functionalities to serve as substrates of deacylases.^12^ Furthermore, the role of these modifications is still poorly understood in many eukaryotic systems, but even more so in prokaryotic systems.^13^

However, proteomic data for many species of Gram-negative and Gram-positive bacteria highlight the preponderance of lysine PTMs on numerous proteins that play critical roles in central carbon metabolism.^14^ Of particular interest is the redoubtable bacterial pathogen *Mycobacterium tuberculosis* (*Mtb*). This bacterium contains thousands of reported sites of lysine-based PTMs, including acetylation, glutarylation, propionylation, succinylation, and malonylation of hundreds of proteins in its proteome,^15–19^ and likely many more sites that have yet to be characterized. These modifications reside on a range of proteins, but are particularly enriched in proteins that play roles in key metabolic pathways within *Mtb*, suggesting that many of the modifications may play a part in regulating metabolism.^15–18^ In the case of *Mtb*, akin to many other prokaryotes, there is only one annotated NAD^+^-dependent SIR gene encoded in its genome (Uniprot ID: P9WGG3), in contrast to the seven encoded sirtuins in humans. Although there are limited detailed studies on the substrate specificity of bacterial Sir2 enzymes, it has been demonstrated that they can efficiently hydrolyze chemically diverse acyl-lysine modifications. For example, the *Escherichia coli* enzyme CobB hydrolyzes acyl-lysine groups ranging from N^ε^-acetyl, to N^ε^-succinyl, N^ε^-lipoamide, and the more recently identified N^ε^-2-hydroxyisobutyryl modification.^20–23^ However, for many of these bacterial deacylases, such as the *Mtb* deacylase, Rv1151c (which we designate *Mt*-Sirt), remain uncharacterized in terms of substrate specificity and kinetic mechanism. Although *Mt*-Sirt has been shown to be nonessential for *Mtb* growth under varied carbon sources,^24–26^ we sought to understand the intricate role(s) that this deacylase plays within this intracellular pathogen, particularly as it has been demonstrated to remove lysine PTMs from varied essential *Mtb* proteins and in some cases alter function of these proteins.^27–35^

Here, we present a kinetic study of *Mt*-Sirt. Analysis of synthetic substrates derived from the sequence of the known *Mt*-Sirt protein substrate, acetyl CoA synthetase (ACS), shows that *Mt*-Sirt is an efficient desuccinylase and fatty deacylase. We characterized nicotinamide, ADP-ribose, and NADH as inhibitors of *Mt*-Sirt and also prepared and characterized thioamide and thiourea lysine analogues, some of which proved to be inhibitors of micromolar potency. We also ascertained that *Mt*-Sirt operates via an equilibrium-ordered kinetic mechanism. In addition, we showed that succinylated isocitrate lyase 1, an essential enzyme for the persistence of *Mtb* infection, undergoes chemical succinylation at key lysine residues required for catalytic activity and is an *in vitro* substrate of *Mt*-Sirt. Finally, we demonstrate that *Mtb* undergoes proteome-wide fatty acylation of lysine/N-terminal glycine residues using the biorthogonal probe tetradec-13-ynoic acid (Alk-12),^36^ suggesting that the *in vitro* NAD^+^-dependent fatty deacylase activity of *Mt*-Sirt may play a yet uncharacterized role.

## Materials and Methods

### Reagents

All chemicals were purchased from commercial providers and used without purification, unless otherwise noted.

### Expression and Purification of Mt-Sirt

A pET28a vector containing the sequence for the Rv1151c gene with an N-terminal hexahistidine tag (a generous gift from the laboratory of the late Dr. John Blanchard) was transformed into the T7 Express *lys* ^*Y*^/*I* ^*q*^ strain of *E. coli*. A single colony was picked and expanded overnight at 37 °C in LB media with 30 μg/mL kanamycin. A 10 mL inoculum was added to 1L of LB media containing 30 μg/mL kanamycin, and grown at 37 °C with shaking at 200 rpm until reaching mid log phase (OD_600_ ∼ 0.6). Protein synthesis was induced by the addition of 1 mM isopropyl-1-thio-β-*D*-galactopyranoside, and the cells were allowed to grow overnight at 25 °C. The cells were harvested by centrifugation (5K rpm) and stored at – 20 °C. The cells were lysed in lysis buffer (50 mM sodium phosphate buffer, 300 mM NaCl, and 10 mM imidazole (pH 7.5)) by sonication (50% duty cycle, ThermoFisher) using a five-second pulse for 20 min with 30 s in between pulses, all while on ice. The resulting solution was centrifuged at 16,000*g* for 45 min at 4 °C to remove cell debris, the supernatant was filtered with a 0.45 μm filter, and the filtrate was then loaded onto a 5 mL Nickel NTA agarose column (ThermoFisher) equilibrated with lysis buffer. The solution was equilibrated on the column for 1 h, and then was washed three times with 50 mM sodium phosphate buffer, 300 mM NaCl, and 25 mM imidazole (pH 7.5). The protein was eluted in 50 mM sodium phosphate buffer, 300 mM NaCl, and 250 mM imidazole (pH 7.5). Following analysis by SDS-PAGE, the fractions containing apparently pure *Mt*-Sirt (*M*_r_ ∼ 26 kDa) were pooled and dialyzed three times against 50 mM Tris-HCl, 150 mM NaCl, pH 7.5. The protein was stored in a 50% (v/v) solution of glycerol at – 80 °C. For the preparation of *Mt*-Sirt mutants we used the overlapping primer design approach and carried out mutagenesis using the Pfu Turbo DNA polymerase (Agilent Technologies). We verified the mutations to the gene by sequencing, and then transformed, expressed, and purified the mutant proteins (Y_53_F and R_56_M) as detailed above.

### Synthesis and Kinetic Characterization of Cbz-N^e^-Acyl-lysine-AMC Substrates

*C*arbobenzyloxy-(N^ε^-acyl) lysine-7-amino-4-methylcoumarin substrates were prepared using varied synthetic routes (detailed in the Supporting Information) and their identities were verified by proton NMR (Bruker) and mass spectrometric analysis (ThermoFisher). Detailed methods on the kinetic characterization of these substrates can be found in the Supporting Information.

### Synthesis and Analysis of Acyl-ACS Peptides

N^ε^-acylated lysine-containing peptides of the sequences Ac-RSG**K**I-*W*–OH for which *W* is a non-naturally occurring tryptophan added to enhance spectrophotometric detection during purification, were synthesized on a 0.1 mmol scale using Fmoc-Trp­(Boc)-Wang resin (obtained from Chem Impex and CEM) on a Liberty Blue automated microwave peptide synthesizer (CEM) under standard conditions. These peptide sequences correspond to residues 614–618 of *M. tuberculosis* acetyl-CoA synthetase (ACS). Two strategies were employed for synthesis of a panel of N^ε^-acylated lysine-containing peptides: (1) synthesis of Fmoc-Lys­(R)–OH amino acids, where R is a predefined chemical group (for detailed information on preparation see Supporting Information), or (2) synthesis of the peptide using Fmoc-Lys­(Mtt)–OH, (Mtt, methyltrityl) which allows selective deprotection of the lysine amine side chain, affording the use of anhydride chemistry or amide-bond coupling to produce the peptide of interest. All peptides were acetylated on the N-terminus with a solution of 25% (v/v) Ac_2_O in N,N-dimethylformamide (DMF) and 1.5 equiv of *N*,*N*-diisopropylethylamine (DIEA), followed by 45 min of agitation at room temperature. A ninhydrin test was used to verify completion of reaction. Select peptides synthesized with an N^ε^-Mtt group underwent treatment with 3% (v/v) TFA in DCM/5% triisopropylsilane for 20 min (2×) to remove the N^ε^-Mtt group. Subsequently the resin was washed with DCM (2×), MeOH (2×), DCM (2×), DMF containing 1% (v/v) DIEA (2×), and DMF (2×). The resulting free ε-amine of lysine was then reacted with an acyl anhydride (10 equiv of anhydride in either DMF or toluene and 1.5 equiv of DIEA) ranging from 1 h to overnight (2×), to give the appropriate modified lysine as monitored by ninhydrin test and LC-MS analysis. The peptides were cleaved from the resin by treatment with 92.5% (v/v) trifluoroacetic acid, 5% (v/v) triisopropylsilane, and 2.5% (v/v) water or 85% (v/v) trifluoroacetic acid, 5% (w/v) phenol, 5% (v/v) thioanisole, and 5% (v/v) water (2× for 2–4 h). The reaction mixture was evaporated under reduced pressure or under a stream of *N*_2(g)_, and the resulting residue was precipitated with ice-cold diethyl ether, dried over *N*_2(g)_, dissolved in DMF, and then purified on a C18 reverse-phase HPLC column using semipreparative HPLC (Luna 5 μm C18(2) 100 Å, 21.2 mm, 250 mm, Phenomenex). Fractions containing peptide that were >90% pure, as determined by analytical reverse phase LC-MS (Luna 5 μm C18(2) 100 Å, 4.6 mm, 250 mm, or Luna 5 μm C18(2) 100 Å, 4.6 mm, 50 mm, Phenomenex) were pooled and lyophilized. Mass spectrometric analysis of the peptides by ESI (ThermoFisher) verified the identity of the peptide(s).

### HPLC Kinetic Analysis of the Deacylation of Peptide Substrates by Mt-Sirt

A typical kinetic assay contained variable concentrations of (0–1.5 mM) N^ε^-acylated peptides, 50 mM Tris-HCl (pH 8.0), 1 mM NAD^+^, 1 mM DTT, 0–10% (v/v) DMSO (no change in *Mt*-Sirt activity was observed from 0–10% (v/v) DMSO), and 0.1–1 μM of *Mt*-Sirt to a final reaction volume of 60 μL. Reaction mixtures were incubated for 5 min at 37 °C at which time reactions were quenched by the addition of 60 μL of 10% (v/v) TFA in water or 0.5 N HCl in MeOH, followed by centrifugation for 10 min at 15,000 rpm to remove the precipitated protein. The 110-μL-supernatants were then loaded into a 96-well plate, and 40-μL aliquots were injected into an HPLC equipped with a Kinetex PS C18 reverse phase column (Kinetex 2.6 μm PS C18 100 Å, LC column 150 × 3 mm, Phenomenex). For peptides that contained ω-carboxy N^ε^-acyl lysines, the gradient used was as follows: a 10 min gradient: 95% H_2_O/5% acetonitrile (ACN) increasing to 60% H_2_O/40% ACN in which all solvents contained 0.1% (v/v) formic acid. For N^ε^ -acyl lysine-containing peptides with N-acyl groups ranging from N-acetyl to N-butyl, we employed a 10 min gradient: 95% H_2_O/5% ACN increasing to 40% H_2_O/60% ACN. For longer acyl groups (hexanoyl to palmitoyl), we used a 10 min gradient initiating at 95% H_2_O/5% ACN and ending at 0% H_2_O/100% ACN. Product formation was measured by the increase of the product peak measured as absorbance at 280 nm in comparison to an enzyme-free control at each substrate concentration tested. The area of the product peak was integrated and divided by the sum of the areas of the substrate and product peaks to measure the fraction of the reaction. This value was then multiplied by the concentration of peptide substrate, which was determined by the extinction coefficient of the peptide (5560 M^–1^-cm^–1^) as measured using a Nanodrop (ThermoFisher) or as determined using a standard curve of Ac-Trp-NH_2_ prepared and analyzed by HPLC. The resulting values were then divided by the time of the reaction to give a rate in μM product/sec. To ensure initial rate conditions, the total product formation was ensured to be less than 10%. Rates were then replotted versus substrate concentration and fitted to the Michaelis–Menten eq (eq 1) using GraphPad Prism 8.

### Steady-State Kinetics of Mt-Sirt

Initial velocity and inhibition studies of wild-type and mutant forms of *Mt-Sirt* were conducted under assay conditions described above, in 50 mM Tris (pH 8.0), containing 1 mM DTT, 0–10% (v/v) DMSO, and fixed concentrations of NAD^+^ (1 mM) and N-acetylated-peptides (200 μM), and 0.1–1 μM of *Mt*-Sirt. In terms of studies to elucidate the kinetic mechanism of *Mt*-Sirt, we conducted an initial velocity study with variable concentrations of NAD^+^ (50–800 μM) at changing-fixed concentration of Ac-RSGK_Succ_IW–OH (10–200 μM). For inhibition studies, changing-fixed concentrations of inhibitor (dissolved in 100% DMSO or water) in 50-μL reaction mixtures containing 50 mM Tris-HCl (pH 8.0), 1 mM DTT, and, in general, 40–200 μM fixed concentrations of Ac-RSGK_**Succ**_I-*W*–OH or 1 mM NAD^+^. Reactions were initiated by the addition *Mt*-Sirt to final concentrations of 0.05–0.2 μM. Reactions were allowed to incubate for 5–30 min at 37 °C, after which time they were quenched with 60 μL of 10% (v/v) TFA solution. Reaction mixtures were then centrifuged for 10 min at 15,000 rpm to remove the precipitated protein. The supernatant (110 μL) was loaded into a 96-well plate and analyzed by HPLC (40-μL injection) on a Kinetex PS C18 reverse phase column (Kinetex; 2.6 μm PS C18 100 Å, LC Column 150 × 3 mm, Phenomenex) with elution using a 10 min gradient: 95% H_2_O/5% acetonitrile (ACN) increasing to 60% H_2_O/40% ACN. All solvents contained 0.1% (v/v) formic acid. Analysis of product peaks and calculation of reaction velocity was conducted identically to that described in the preceding section. Plotting of the rate in μM s^–1^ versus inhibitor concentrations were used to determine values of IC_50_ upon data fitting to eqs 7 and 8. Product inhibition studies of NAM (0–800 μM) vs NAD^+^(50–750 μM) and of NAM (0–400 μM) vs Ac-RSGK_Succ_IW–OH (10–200 μM) were performed in kind, as were dead-end inhibition studies of ADPR (0–5000 μM) vs NAD^+^(50–750 μM) and vs Ac-RSGK_Succ_IW–OH (10–200 μM), and Cbz-Lys-AMC­(0–2000 μM) vs NAD^+^(50–750 μM) and vs Ac-RSGK_Succ_IW–OH (10–200 μM).

### Mt-Sirt-Catalyzed Desuccinylation of Chemically Succinylated Mtb ICL1

Recombinant *Mtb* ICL1 protein was prepared as previously described^37,38^ and incubated at 1 mg/mL (20 μM) in 50 mM HEPES (pH 7.5), 150 mM NaCl, 5 mM MgCl_2_ with succinyl-CoA (500 μM) for 3h at 37 °C at a volume of 50 μL. The reaction mixture containing succinylated *Mt*ICL1 was diluted into Assay Buffer (450 μL, 50 mM HEPES (pH 8.5), 137 mM NaCl, 2.7 mM KCl, 1.25 mM MgCl_2_), followed by buffer exchange into assay (4×) via centrifugation using an Amicon Ultra-0.5 mL filter, 30-kDa molecular weight cutoff, 10k rpm. Protein concentration was quantified via Nanodrop (ThermoFisher) and by use of the BCA protein assay (ThermoFisher). Subsequently, 5 μM of succinylated-*Mt*ICL1 and 1 μM of *Mt*-Sirt were added to the *Mt*-Sirt Assay Buffer, followed by the initiation of the reaction by the addition of the appropriate concentrations of substrates and/or inhibitors (1 mM NAD^+^ and 5 mM NAM), to a final volume of 30 μL. The reactions were quenched at indicated time points by the addition of SDS-PAGE loading dye to the samples, followed by heating at 95 °C for 10 min. The protein samples were then separated by SDS-PAGE (Bio-Rad 4–15% Mini-PROTEAN TGX Stain-Free protein gel) and semidry transferred (Bio-Rad) onto an activated PDVF membrane (GE Healthcare Life Sciences) followed by blocking with TBST (1× Tris-buffered saline, 0.1% Tween-20) with 5% wt/v BSA (1 h, RT). Antisuccinyl lysine rabbit polyclonal antibody (PTM Biolabs) was added at a 1:6000 dilution, and the blot was incubated overnight at 4 °C. The membrane was washed 5× with TBST, probed with goat-anti rabbit HRP conjugate antibody (1:4000) (Bio-Rad) in TBST/5% (wt/v) BSA (1h, RT), washed (5×, TBST) followed by addition of ECL substrate (Bio-Rad), and imaged on a ChemiDoc (Bio-Rad).

### Bio-orthogonal Labeling of mc^2^7000 Cells

A detailed procedure for the treatment of mc^2^7000 cell lysates with tetradec-13-ynoic acid (Alk-12, Click Chemistry Tools), followed by covalent labeling of the alkynyl group with Cy7-azide (Click Chemistry Tools) as adapted from Mellott et al.^39^ may be found in the Supporting Information.

## Data Analysis

Initial velocity data obtained at variable concentrations of a single substrate A at fixed concentrations of the other substrate were fitted to (eq 1) in which *v* is the initial velocity,

*E*_t_ is the enzyme concentration, *V*_max_ is the maximum velocity, *k*_cat_ is the turnover number, *A* is the concentration of the variable substrate, and *K*_a_ is the apparent Michaelis constant. Two-substrate initial velocity data obtained at variable concentrations of a single substrate *A* and at changing-fixed concentrations of a second substrate B were fitted to (eqs 2 and 3), in which *K*_ia_ and *K*_a_ are the respective dissociation and Michaelis constants of the dissociation constant of *A* and *K*_b_ is the Michaelis constant of *B*. Data obtained for product and dead-end inhibitors were first plotted in double-reciprocal form, and plots were inspected for slope and intercept effects resulting from the added inhibitor. Data which appeared to conform to competitive, noncompetitive, or uncompetitive inhibition, respectively, were then fitted to eqs 4–6 for which *K*_m_ is the Michaelis constant, and *K*_is_ and *K*_*ii*_ are the apparent slope and intercept inhibition constants, respectively.

1 $$v=\frac{V_{\max}\left[A\right]}{K_{a}+\left[A\right]}=\frac{k_{\mathrm{cat}}\left[E_{t}\right]\left[A\right]}{K_{a}+\left[A\right]}$$

2 $$v=\frac{V_{\max}\left[A\right]\left[B\right]}{K_{\mathrm{ia}}K_{b}+K_{b}\left[A\right]+K_{a}\left[B\right]+\left[A\right]\left[B\right]}$$

3 $$v=\frac{V_{\max}\left[A\right]\left[B\right]}{K_{\mathrm{ia}}K_{b}+K_{b}\left[A\right]+\left[A\right]\left[B\right]}$$

4 $$v=\frac{V_{\max}\left[S\right]}{K_{m}\left(1+\frac{I}{K_{\mathrm{is}}}\right)+\left[S\right]}$$

5 $$v=\frac{V_{\max}\left[S\right]}{K_{m}\left(1+\frac{I}{K_{\mathrm{is}}}\right)+\left[S\right]\left(1+\frac{I}{K_{\mathrm{ii}}}\right)}$$

6 $$v=\frac{V_{\max}\left[S\right]}{K_{m}+\left[S\right]\left(1+\frac{I}{K_{\mathrm{ii}}}\right)}$$

7 $$Y=\frac{y_{\min}+\left(y_{\max}-y_{\min}\right)}{\left(1+10^{\left(\log\mathrm{IC}50-\left[\log I\right]*h\right)}\right)}$$

Other inhibition data were fitted to eq 7, in which IC_50_ is the concentration of inhibition that reduces the enzyme activity to 50%, *y*_min_ and *y*_max_ represent the lower and upper limits of the percent reduction of enzyme activity, and *h* is the Hill slope and the top was *y*_max_ was fixed at 100%. The apparent inhibition constant was obtained by fitting data eq 8.

8 $$K_{i}=\frac{{\mathrm{IC}}_{50}}{\left(1+\frac{\left[A\right]}{K_{m}}\right)}$$

## Results and Discussion

### *Mt*-Sirt Contains an Active Site YxxR Motif

Sequence alignment of *Mt-*Sirt with other known bacterial and mammalian sirtuins, revealed that *Mt-*Sirt contained a YxxR motif (Y_53_ and R_56_), which is also found in human sirtuin 5 (hSIRT5) and bacterial sirtuins from *E. coli* and *Salmonella typhimurium* (CobB) (Figure 2A). Therefore, to try to better understand how this enzyme functions, we created a homology model of the protein using AlphaFold (Figure 2B).^40,41^ Overlay of the homology model with crystal structures of archaeal, human, and bacteria sirtuins assisted in identifying key motifs required for substrate recognition.^42^ Initially, superposition with the structure of *E*. *coli* CobB (PDB: 6RXJ) bound to an N^ε^-acetyl-lysine peptide resulted in a moderate overall structural homology with *Mt*-Sirt (RMSD: 1.01 Å) (Figure 2C). In particular, our attention was drawn to helix bundle six, which contains the YxxR motif of CobB (Y_92_ and R_95_) (Figure 2C,D). While the tyrosine residues of both proteins overlaid favorably, we noticed that the guanidinium group of R_95_ in *E. coli* CobB pointed away from the active-site cavity, while the R_56_ of *Mt*-Sirt pointed inward toward this cavity. The positioning of R_95_ away from the active-site cavity may be due to repulsion between the hydrophobic methyl group of the bound acetyl-lysine peptide and the positive guanidinium group of R_95_. Further, this “shift” is allowed as the area proximal to R_95_ in the CobB structure contains a polar Q_98_ residue that may afford hydrogen bonding with R_95_ guanidinium. Conversely, this type of movement is unlikely to be observed in *Mt*-Sirt as the Q_98_ has been replaced with a leucine (L_59_).

**Figure 2:**
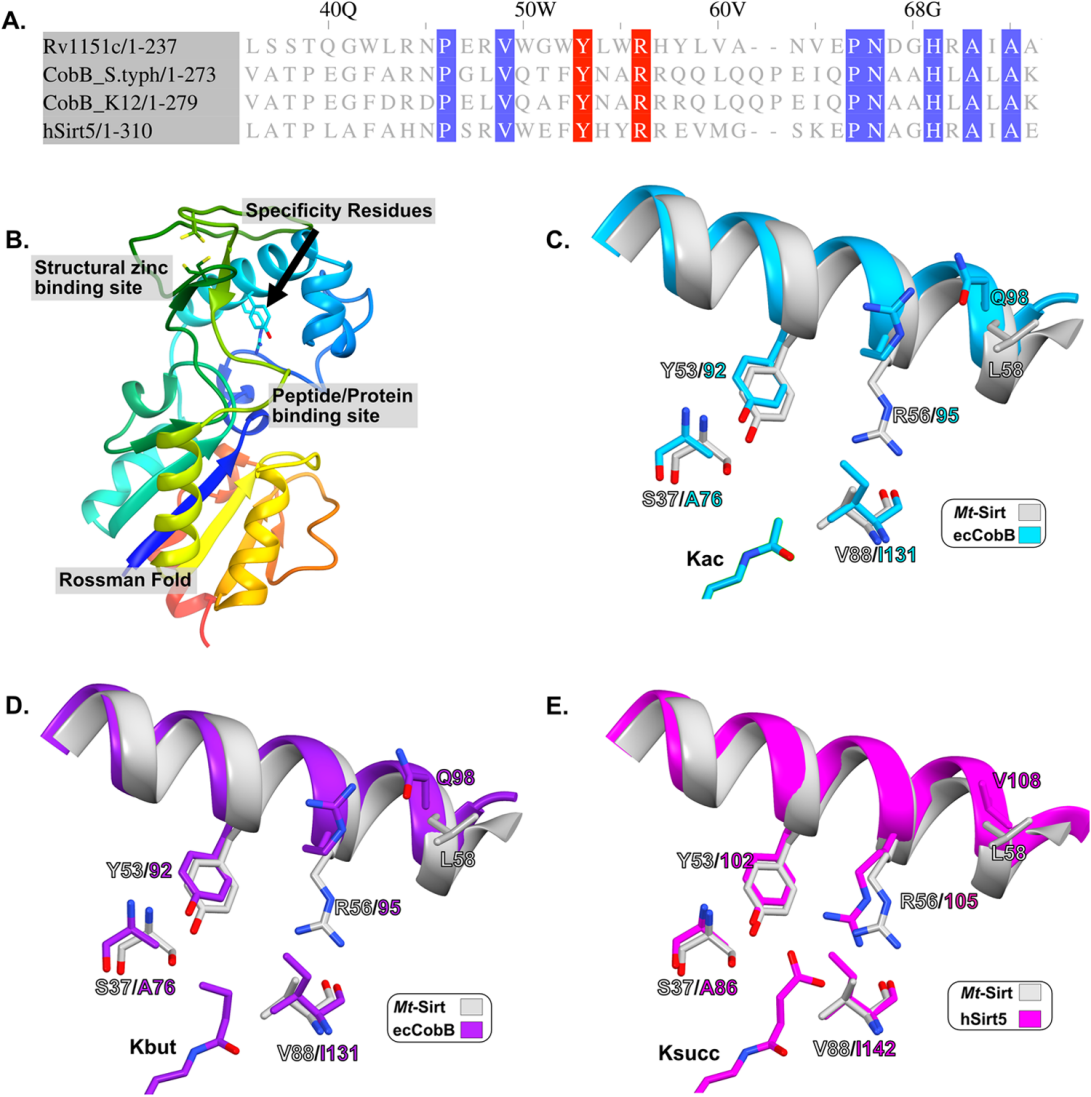
Sequence homology of *Mt*-Sirt and homology structure overlay (A) Sequence alignment of Rv1151c (*Mt*-Sirt) with two bacterial NAD^+^-dependent deacylases and human sirtuin 5. Blue highlighted regions demarcate 80% identity, and the red highlighted regions correspond to the YxxR motif of the enzymes. (B) AlphaFold model of *Mt*-Sirt. (C) Overlay of *Mt*-Sirt AlphaFold homology model (gray) with *E. coli* CobB (PDB: 6RXJ) (blue) bound to an acetylated peptide (Kac, blue). (D) Overlay of *Mt*-Sirt AlphaFold homology model (gray) with *E. coli* CobB (PDB: 6RXK) (magenta) bound to a butyrylated peptide (Kbut, magenta). (E) Overlay of *Mt*-Sirt AlphaFold homology model (gray) with human sirtuin 5 (PDB: 3RIY) (pink) bound to a succinyl peptide (Ksucc, pink).

Beyond this arginine, it has been shown in CobB that A_76_ in concert with I_131_ may play a role in the efficient catalysis of short-chain fatty acyl peptides through a substrate-induced-fit mechanism.^43^ Interestingly, overlay of *Mt*-Sirt with CobB revealed that in lieu of an alanine at this position, *Mt*-Sirt contained a serine (S_37_) (Figure 2C). Close inspection of CobB bound to an N^ε^-butyryl lysine peptide (PDB: 6RXK) revealed that the methyl group of the N^ε^-butyryl lysine group resides proximal to the A_76_ (3.4 Å) of CobB, whereas in *Mt*-Sirt, the polar hydroxyl of S_37_ compared to the more hydrophobic methyl of A_76_, may hinder the binding of short-chain hydrophobic lysine residues (Figure 2D). Subsequent alignment of the *Mt*-Sirt model with the human sirtuin 5 (hSIRT5, a desuccinylase) bound to a succinyl peptide (PDB: 3RIY) resulted in clear overlay of both the R and Y side chains protruding from the helix bundle (Figure 2E). In addition, hSIRT5 and *Mt*-Sirt both contain a hydrophobic residue (V_108_ and L_58_, respectively) where the Q_98_ residue was observed in CobB. This presumed inability to mask the polar guanidinium group may play a role in the limited tolerance of hSIRT5 for short-chain alkyl lysine PTMs^44,45^ when compared to *E. coli* CobB.^46^ Together, this information suggests that *Mt*-Sirt may prefer ω-carboxy-alkyl modifications of lysines, such as succinyl, when compared to shorter chain hydrophobic PTMs such as an acetyl group.^21,44^

### *Mt-*Sirt Shows a Preference for N^ε^-Succinyl-lysine-Containing Substrates

It has been previously shown that *Mt-*Sirt can utilize carbobenzyloxy-(N^ε^-acetyl)­lysine-7-amino-4-methylcoumarin (Cbz-Lys­(N^ε^-acetyl)-AMC) as a substrate, albeit no detailed kinetic data were reported.^47^ Therefore, to test the ability of *Mt-*Sirt to hydrolyze the amide bond of N^ε^-acyl-lysine-containing peptide substrates, we synthesized six different Cbz-Lys­(N^ε^-acyl)-AMC substrates containing six N-acyl groups that are commonly found on modified lysines. (Figure 3A). Initial analysis demonstrated that *Mt-*Sirt catalyzed deacylation of all Cbz-N^ε^-amido-lysinyl-AMC compounds tested, but the most robust substrate was Cbz-Lys­(N^ε^-succ)-AMC (Figure 3B). Kinetic analysis determined that *Mt*-Sirt bound this succinyl-lysine substrate 10-fold tighter (*K*_m_ = 16.5 μM versus 157 μM) and catalyzed the deacylation 1.6-fold faster (*k*_cat_ = 0.015 versus 0.009 s^–1^) when compared to its acetyl counterpart (Figure S1A,B). While we were unable to conduct a complete kinetic analysis of the remaining substrates due to poor solubility, we did note that *Mt*-Sirt exerted poor demalonylase and deglutarylase activity, suggesting that the N^ε^-succinyl lysine PTM may provide the optimal binding interaction with the active site Y_53_ and R_56_ residues. In addition, the poorer activity observed with short-chain hydrophobic lysine PTMs may result from weak binding to the enzyme (substrate saturation was not observed up to 250 μM for propionyl- and butyryl-lysine substrates (Figure S1C)) due putatively to the polar character of R_56_ or S_37_.

**Figure 3:**
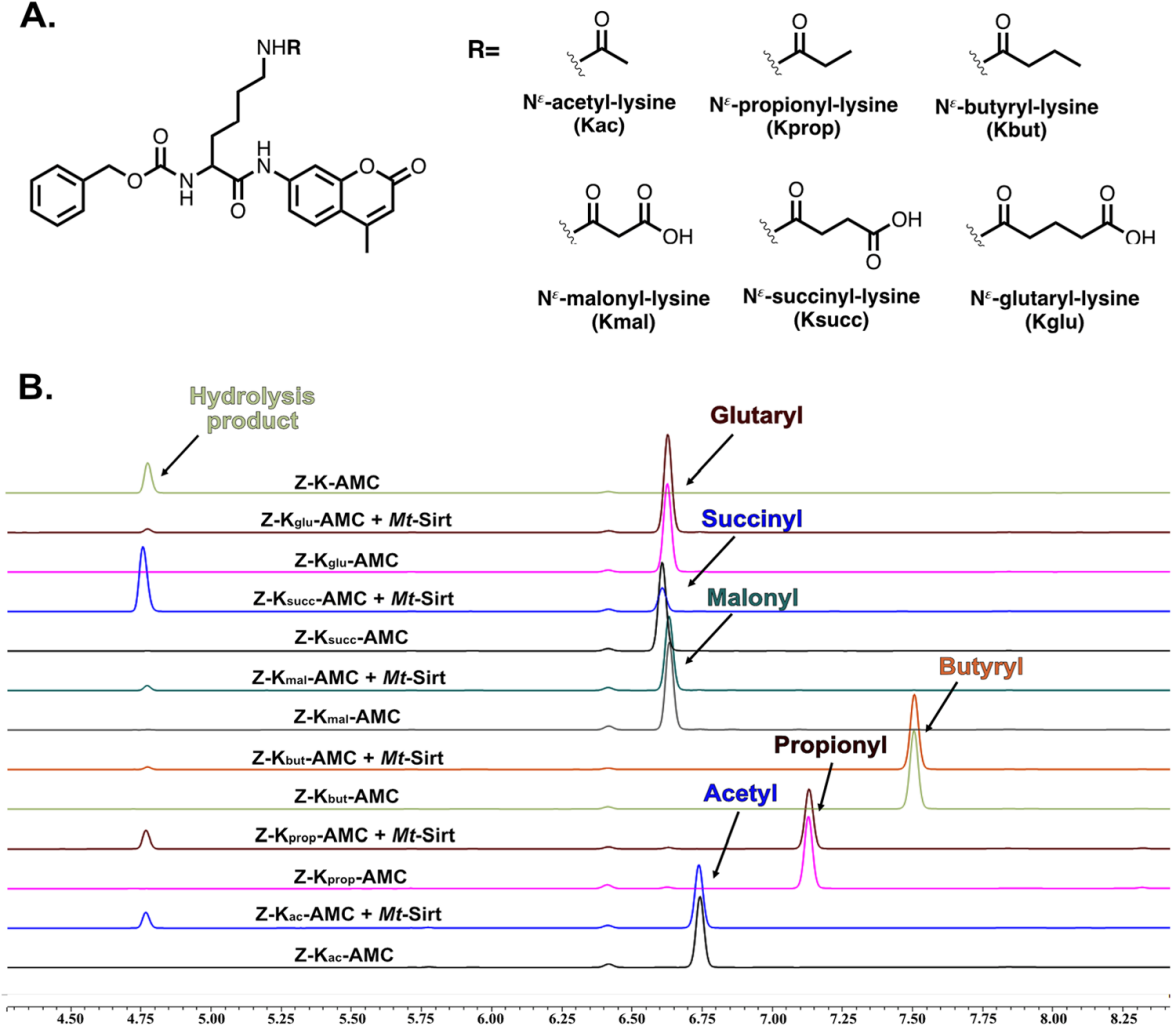
*Mt*-Sirt preferentially catalyzes the deacylation of N^ε^-succinyl lysine *in vitro*. (A) Panel of Cbz-N^ε^-acyl-lysine-AMC substrates. (B) Chromatograms demonstrating the *Mt*-Sirt-catalyzed hydrolysis of the different acyl side chains of the monolysine-AMC substrates shown in (A). Reactions were carried out with 50 μM substrate, 1 μM *Mt*-Sirt, 50 mM Tris-HCl (pH 8.0), 1 mM NAD+ and 1 mM DTT, at 37 °C for 30 min followed by quenching with 10% TFA (v/v) in water.

### Design and Synthesis of Peptide Substrates Derived from Acetyl CoA Synthetase

The modified lysine residues in the Cbz-N^ε^-Lys­(R)-AMC substrates are flanked by bulky hydrophobic groups, and despite their activity as substrates, they were poor substitutes for the naturally occurring N^ε^-acyl-oligopeptides and N^ε^-acyl-protein substrates of *Mt-*Sirt. Accordingly, we prepared a series of oligopeptide substrates to gain further insight into the substrate specificity of this enzyme. These peptides were derived from a deacetylation site of acetyl-CoA synthetase (ACS), specifically the sequence encompassing lysine 617 (-RSG**K**IMR-). This lysine residue has been shown to be acetylated by the action of *Ms*-Pat, a cAMP-dependent, GCN5-related N-acetyltransferase (GNAT) which leads to specific inactivation of the formation of the acetyl-adenylate reaction intermediate ACS.^28^ Restoration of ACS activity is obtained by the removal of the acetyl group from ACS K_617_ by *Mt*-Sirt, underscoring how lysine PTMs can provide *Mtb* a reversible regulatory mechanism to fine-tune the activity of metabolic enzymes. Hexapeptides based on the sequence of the modified lysine in ACS (Ac-**Arg-Ser-Gly-Lys­(R)-Ile-**Trp-OH) were prepared, in which five of these residues comprised the ACS K_617_ sequence (shown in bold). The sixth residue is a C-terminal tryptophan residue added to enable spectrophotometric detection during analysis and purification by HPLC. In these peptides, the acyl groups (**R**) encompassed a range of known lysine PTMs (Figure 4A). Together, these substrates provide ample chemical diversity that allowed us to probe the substrate specificity of *Mt*-Sirt.

**Figure 4:**
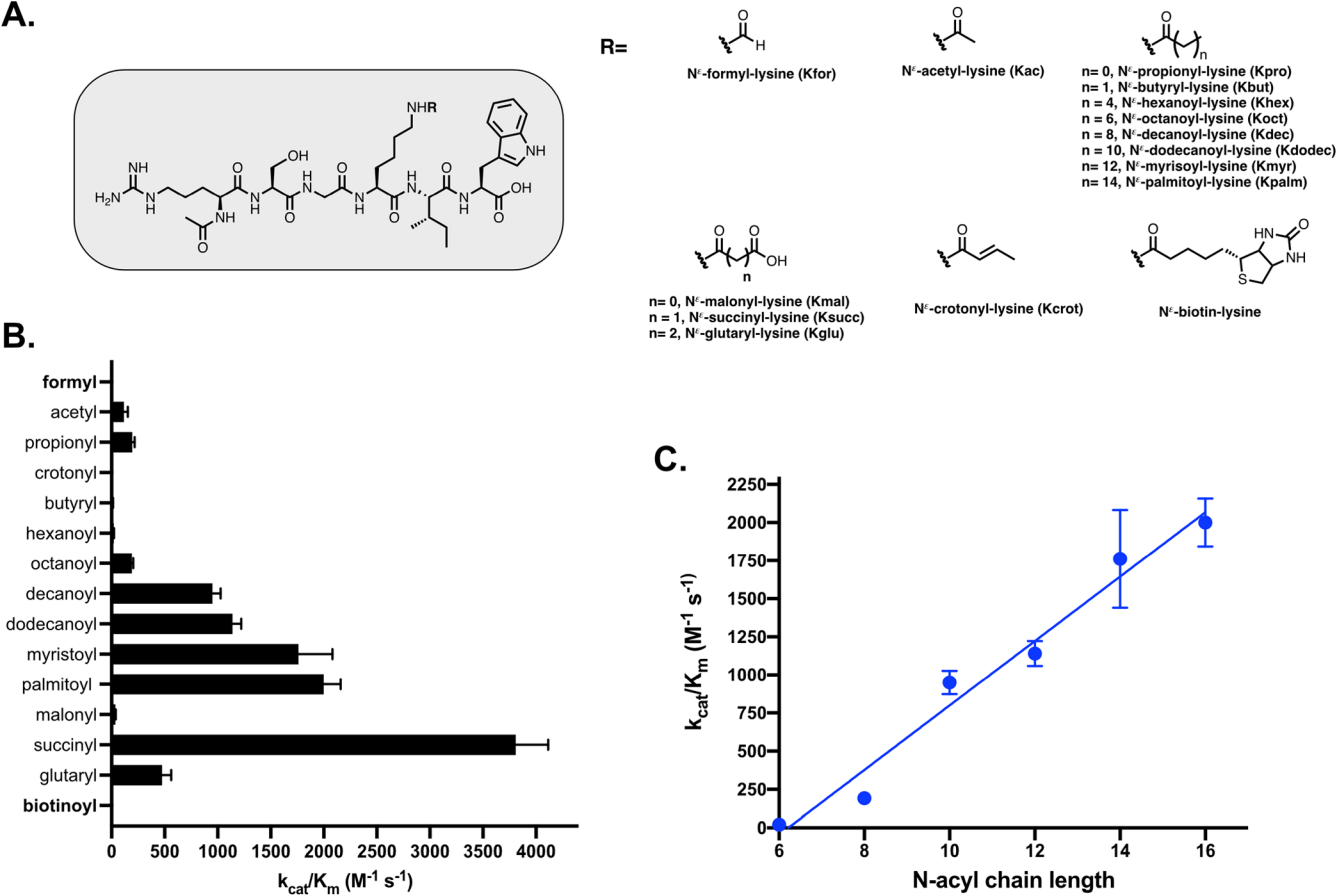
Kinetic analysis of peptides derived from ACS-K617. (A) The hexapeptide scaffolds derived from the residues flanking ACS-K617 and the N^ε^-acyl substituents used in this study. (B) Summary of kinetic analysis of peptides as shown by their specificity constants (*k*_cat_/*K*_m_). Values for crotonyl, butyryl, and hexanoyl were obtained, but are not readily visible in the plot due to their poor kinetic constants. All kinetic constants can be found in Table 1 with data found in Figure S2. (C) Plot of the N^ε^-acyl chain length (hexanoyl to palmitoyl) versus the specificity constant (*k*_cat_/*K*_m_). The line drawn through the data was determined by fitting to y= mx+b.

### *Mt*-Sirt Exerts Robust Lysine Desuccinylase and Fatty-Acid Deacylase Activity

Characterization of *Mt*-Sirt deacylase activity using synthetic hexapeptides derived from amino acids 614–618 of acetyl-CoA synthetase was performed at saturating NAD^+^ (1 mM) and variable peptide concentrations to ascertain the steady-state kinetic parameters *k*_cat_, *K*_m,_ and *k*_cat_/*K*_m_ (Table 1 and Figure 4B). Kinetic data for 15 hexapeptides in which the lysine side chain was modified to N^ε^-formyl, N^ε^-acetyl, and increasingly larger N^ε^-alkyl-amide side chains are shown in Table 1. The N^ε^-formyl-lysine substrate exhibited negligible activity such that we were unable to determine values of *k*_cat_/*K*_m_ or *k*_cat_. The values of *k*_cat_/*K*_m_ and *k*_cat_ for the hexapeptide containing N^ε^-acetyl were 120 M^–1^s^–1^ and 0.12 s^–1^, respectively, and these values diminished appreciably for alkyl side chains of 4–6 carbon atoms.

**Table 1:** Steady-State Kinetic Parameters of Variable Ac-RSGK_Acyl_IW–OH Peptides with *Mt*-Sirt^a^

**acyl group in Ac-RSGK**_**Acyl**_ **IW–OH**	***k***_**cat**_ **/** ***K***_**m**_ **(M** ^**–1**^ **s** ^**–1**^ **)**	**relative** ***k***_**cat**_ **/** ***K***_**m**_	***k***_**cat**_ **(s** ^**–1**^ **)**	**relative** ***k***_**cat**_	***K***_**m**_ **(μM)**
**alkyl**					
acetyl	(1.1 ± 0.1) × 10^2^	0.03	0.112 ± 0.02	0.71	1100 ± 100
propionyl	(1.9 ± 0.2) × 10^2^	0.05	0.078 ± 0.003	0.50	410 ± 40
butyryl	(1.3 ± 0.1) × 10^1^	0.004	0.017 ± 0.001	0.11	1300 ± 100
crotonyl	(1.7 ± 0.5) × 10 °	0.0004	0.007 ± 0.001	0.04	4000 ± 900
hexanoyl	(1.7 ± 0.2) × 10^1^	0.004	0.017 ± 0.001	0.11	100 ± 60
octanoyl	(1.9 ± 0.1) × 10^2^	0.05	0.036 ± 0.001	0.23	180 ± 10
decanoyl	(9.5 ± 0.8) × 10^2^	0.25	0.023 ± 0.001	0.15	24 ± 2
dodecanoyl	(1.1 ± 0.1) × 10^3^	0.29	0.047 ± 0.002	0.30	42 ± 3
myristoyl	(1.8 ± 0.3) × 10^3^	0.47	0.031 ± 0.002	0.20	18 ± 3
palmitoyl	(2.0 ± 0.2) × 10^3^	0.53	0.016 ± 0.004	0.10	8 ± 1
**carboxy-alkyl**					
malonyl	(3.6 ± 0.4) × 10^1^	0.01	0.048 ± 0.003	0.31	1400 ± 100
succinyl	(3.8 ± 0.3) × 10^3^	1.00	0.157 ± 0.004	1.00	42 ± 3
glutaryl	(5.2 ± 0.9) × 10^2^	0.14	0.055 ± 0.004	0.35	110 ± 20

a All above values were determined by fittingthe data obtained by monitoring the rate of deacylated peptide product formation versus the depletion of starting acyl peptide via an HPLC assay followed by fitting of *v* vs [acyl peptide] with eq 1 (Figure S2).

Interestingly, values of *k*_cat_/*K*_m_ increased and those of *K*_m_ decreased as the alkyl side chains lengthened from 8–14 carbons, for which the N^ε^-myristoyl- and N^ε^-palmitoyl-lysyl substrates have the highest (and comparable) values of *k*_cat_/*K*_m_ (1800 and 2000 M^–1^ s^–1^, respectively). Plotting of catalytic efficiency (*k*_cat_/*K*_m_) versus fatty-acyl chain length (hexanoyl to palmitoyl) of the peptide substrate revealed a linear correlation (*R* ^2^ = 0.95), (Figure 4C). However, values of *k*_cat_ decreased linearly when the side chains exceeded 8 carbon atoms. The value of *k*_cat_ for the Ac-RSGK_Palm_IW–OH substrate is only 10% of that of Ac-RSGK_Succ_IW–OH (Table 1), so that observed enhancement of values of *k*_cat_/*K*_m_ for these longer-chain substrates was attributable solely to decreases in values of *K*_m_. The lower values of *k*_cat_ suggest poorer orientation of these substrates for catalysis.

To assess if *Mt-*Sirt can also tolerate bulky N^ε^-acyl modified peptides in addition to fatty acyl peptides, we prepared an N^ε^-biotinoyl hexapeptide. Incubation of *Mt*-Sirt with this substrate yielded no product formation (data not shown), suggesting that *Mt*-Sirt does not tolerate bulky N^ε^-acyl modified lysine residues and instead has a preference for linear N^ε^-fatty acyl modified lysines (octanoyl to palmitoyl).

The finding that *Mt*-Sirt catalyzes deacylation of a long fatty acyl substrate nearly as well as its desuccinylase activity comprises a distinct characteristic of this enzyme, and this apparent dual activity may be a result of it being the only sirtuin encoded in *Mtb*. That *Mt*-Sirt is a desuccinylase is unsurprising in that its modeled structure is similar to that of SIRT5 (Figure 2), and both enzymes have the conserved YxxR sequence that is involved in binding of the carboxylate group of the succinyl-lysine substrate. However, it is unclear how the binding site of *Mt*-Sirt can well accommodate long-chain fatty-acyl-containing substrates, as is the case for SIRT6. SIRT6 was the first NAD^+^-dependent fatty deacylase discovered and has a large hydrophobic tunnel that allows for interaction with long alkyl chains appended to the N^ε^ amine of a lysine-containing substrate.^48^ However, it was later discovered that other sirtuins (including SIRT5 with a polar active site YxxR motif akin to *Mt*-Sirt) demonstrated fatty deacylase activity, suggesting that this class of enzymes has some substrate promiscuity.^45^ To better understand the *Mt*-Sirt fatty deacylase activity from a structural standpoint, we overlaid our AlphaFold model of *Mt*-Sirt with crystal structures of human SIRT2, SIRT3, and SIRT6, the latter of which were all bound to an N^ε^-myristoyl-lysine-containing peptide. Superposition of key SIRT2 residues reported to be involved in forming a hydrophobic channel for substrate binding^49^ with those of *Mt*-Sirt showed a marked shift from the aromatic amino acids in the SIRT2 hydrophobic channel (F_119_, F_131_, Y_139_, F_142_, and F_190_) to aliphatic amino acids in *Mt*-Sirt hydrophobic channel (L_43_, V_49_, L_59_, V_63_, L_107_, respectively) (Figure S3A). Overlay of *Mt*-Sirt with SIRT6 bound to an N^ε^-myristoyl-lysine peptide (PDB: 3ZG6)^48^ indicated relatively poor conservation of those SIRT6 residues proposed to be important in N^ε^-myristoyl-lysine binding based on the previous reports (Figure S3B).

Despite the poor structural overlay and sequence conservation of the *Mt*-Sirt model with the key residues responsible for myristoyl group binding in SIRT2 and SIRT6, the myristoyl group also directly clashed with the YxxR motif of *Mt*-Sirt in both structures. Interestingly, overlay of *Mt*-Sirt with SIRT3 bound to a myristoyl-lysine^50^ revealed that the myristoyl-binding pocket of hSIRT3 is well conserved with that of *Mt*-Sirt, and the alkyl chain did not clash with the YxxR motif of *Mt*-Sirt (Figure S3C). Structural superposition identified 15 residues lining the acyl-lysine binding channel of hSIRT3 (A_146_, T_150_, I_154_, F_157_, R_158_, F_180_, L_199_, I_230_, H_248_, V_292_, F_294_, G_295_, E_296_, L_298_, E_325_), of which nine are strictly identical in *Mt*-Sirt (60%). Further, three positions carry conservative substitutions (Thr_150_→Ser, Phe_180_→Trp, and Ile_230_→Val) that preserve the physicochemical character of the pocket and minimally alter its overall geometry or hydrophobicity. Overall, this preliminary analysis may suggest that *Mt*-Sirt fatty deacylase activity arises from its similar active site architecture to SIRT3. To better elucidate the precise underpinnings of *Mt*-Sirt fatty deacylase activity, determination of a crystal structure of *Mt*-Sirt in complex with an N^ε^-fatty acyl-lysine peptide should be the focus of future work.

Consistent with our previous analysis of the Cbz-N^ε^-acyl-lysine-AMC substrates, *Mt*-Sirt-catalyzed hydrolysis of Ac-RSGK_Succ_IW–OH displayed the highest level of catalytic efficiency of all of the hexapeptide substrates tested (Table 1, *k*_cat_/*K*_m_ = 3.8 ± 0.3 × 10^3^ M^–1^ s^–1^). When an N^ε^-butyryl-lysyl group was present, the relative values of *k*_cat_/*K*_m_ and *k*_cat_ were, respectively, 0.4% and 11% that of Ac-RSGK_Succ_IW–OH, suggesting that the β-carboxyl group of the succinyl side chain likely forms a hydrogen-bonding or electrostatic interaction with R_56_ and possibly Y_53_ on *Mt-*Sirt for both substrate recognition and catalysis, as has been proposed for hSIRT5.^44,51^ Remarkably, the hexapeptide substrates containing a N^ε^-malonyl-lysyl or N^ε^-glutaryl-lysyl residue comprise, respectively, a “retraction” or “elongation” of this β-carboxyl group by a single methylene unit, yet resulted in a both cases with nearly identical reductions in *k*_cat_ values and relative values *k*_cat_/*K*_m_ that were 1 and 14%, respectively, of that of Ac-RSGK_Succ_IW–OH. From this it appears that the N^ε^-succinyl-lysyl residue is ideally suited for deacylation by *Mt-*Sirt.

To further examine this specificity, we conducted kinetic analysis of R_56_M and Y_53_F mutants of *Mt-*Sirt to ascertain how the removal of the polar hydroxyl of the phenolic Y_53_ and effective neutralization of the cationic guanidinium group of R_56_ altered the ability of the enzyme to use the Ac-RSGK_Prop_IW–OH and Ac-RSGK_Succ_IW–OH substrates (Table 2). For the Y_53_F mutant, the *K*_m_ of the Ac-RSGK_Prop_IW–OH peptide increased 4.6-fold (compare 410 to 1900 μM), and the *k*_cat_ slowed nearly 2-fold (0.08 s^–1^ to 0.058 s^–1^) resulting in a 6.3-fold decrease (compare 190 to 30 M^–1^s^–1^) in catalytic efficiency (*k*_cat_/*K*_m_). The lack of saturation observed with the Y_53_F enzyme in the presence of the propionyl substrate likely yielded an inflated *k*_cat_ value (Figure 5A and Table 2). Therefore, Lineweaver–Burke analysis (Figure S4) was used and yielded a *k*_cat_ of 0.022 ± 0.08 s^–1^, which corresponds to a 4-fold decrease versus the wild-type enzyme, highlighting the importance of the Y_53_ residue for effective catalysis of this substrate. Deacylation of the substrate Ac-RSGK_Succ_IW–OH by the Y_53_F mutant yielded values of *k*_cat_/*K*_m_ and *K*_m_ of 320 M^–1^ s^–1^ and 260 μM, respectively, and a *k*_cat_ of 0.09 s^–1^. Compared to the wild-type enzyme, this resulted in a 11.9-fold lower *k*_cat_/*K*_m_ (3800 vs 320 M^–1^ s^–1^), a 6.1-fold higher *K*_m_ (42 vs 260 μM) and a 0.6-fold lower rate of *k*_cat_ (0.157 vs 0.09 s^–1^) (Figure 5B and Table 2). Of interest, both substrates seemed to have similar effects on the three kinetic parameters studied, suggesting that the phenolic group of Y_53_ assists in both binding and catalysis of N^ε^-acyl lysyl peptide substrate regardless of the nature of the side chain, but likely in different fashions. For the Ac-RSGK_Prop_IW–OH substrate, it is likely that the Y_53_F substitution may perturb the conformation of key active site residues required for the efficient binding and catalysis. However, for the Ac-RSGK_Succ_IW–OH substrate, this alteration more likely arises from the loss of a hydrogen-bonding interaction with the β-carboxyl group of succinyl-lysine, resulting in less avid binding and poorer stabilization of the Michaelis complex.

**Figure 5:**
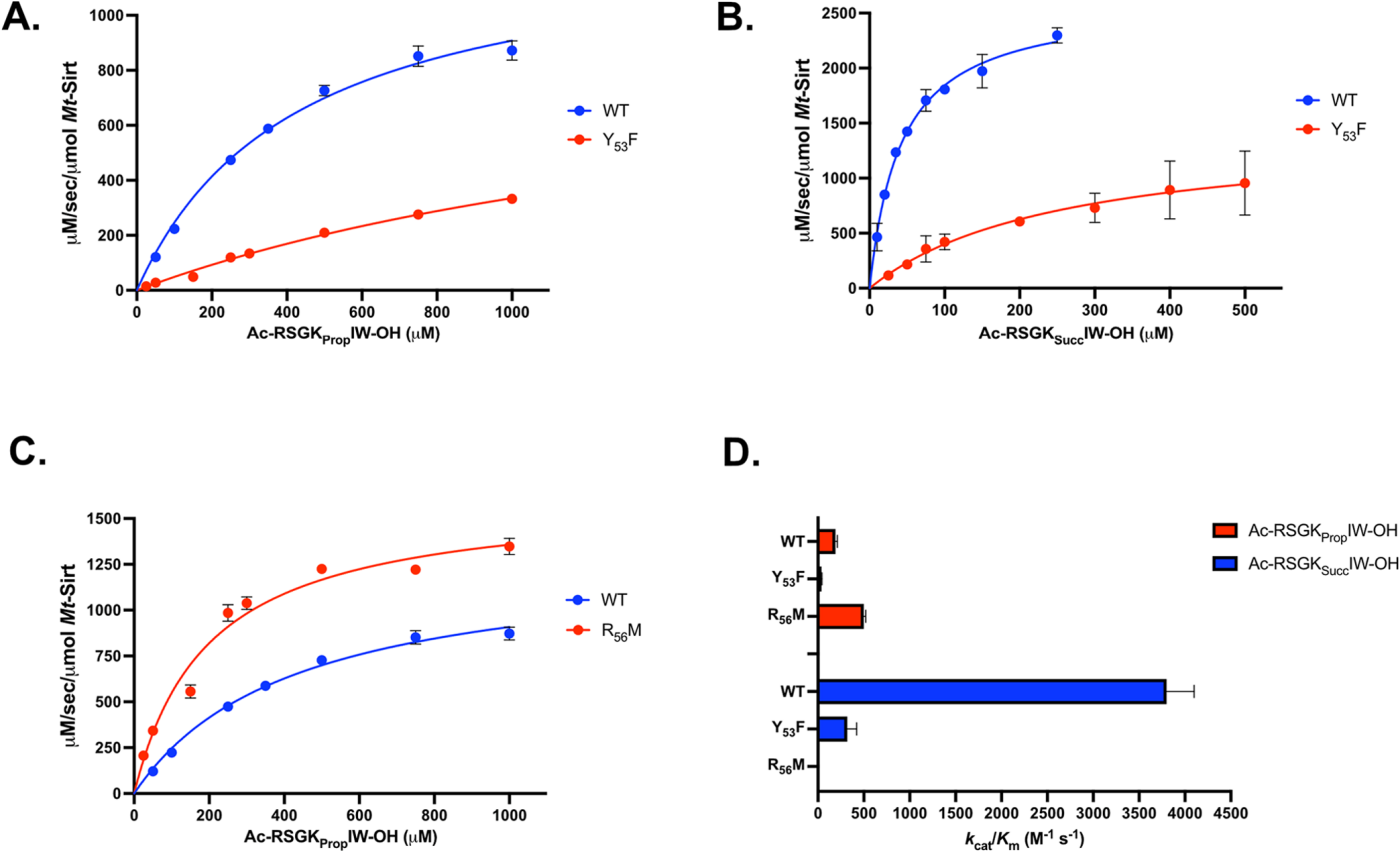
Initial velocity data of WT and *Mt*-Sirt mutants using (A) WT and Y53F *Mt-*Sirt with Ac-RSGKPropIW–OH. (B) WT and Y53F *Mt-*Sirt with Ac-RSGKSuccIW–OH. (C) WT and R56 M *Mt-*Sirt with Ac-RSGKPropIW–OH. (D) Plot showing the *k*_cat_/*K*_m_ of wild-type and mutant *Mt*-Sirt with the respective peptides tested. All lines drawn through experimental data were from fitting to eq 1.

**Table 2:** Kinetic Analysis of Ac-RSGK_Prop_IW−OH and Ac-RSGK_Succ_IW−OH with Y_53_F and R_56_M Mutants of *Mt*-Sirt

**substrate**	**enzyme**	***k***_**cat**_ **(s** ^**–1**^ **)**	***K***_**m**_ **(μM)**	***k***_**cat**_ **/** ***K***_**m**_ **(M** ^**–1**^ **s** ^**–1**^ **)**
Ac-RSGK_Prop_IW–OH	WT	0.078 ± 0.003	410 ± 40	(1.9 ± 0.2) × 10^2^
	Y_53_F	0.058 ± 0.008^a^	1900 ± 350	(3.0 ± 0.7) × 10^1^
	R_56_M	0.10 ± 0.005	200 ± 30	(5.0 ± 0.1) × 10^2^
Ac-RSGK_Succ_IW–OH	WT	0.16 ± 0.004	42 ± 3	(3.8 ± 0.3) × 10^3^
	Y_53_F	0.09 ± 0.01	260 ± 90	(3.2 ± 1.0) × 10^2^
	R_56_M	N/A^b^	N/A^b^	N/A^a^

a Likely overestimated due to the lack of substrate saturation.

b No detectable product was formed in the presence of 200 μM Ac-RSGK_Succ_IW–OH and 1 μM *Mt*-Sirt.

In the case of the R_56_M mutant of *Mt*-Sirt, significant differences in the kinetic parameters of the two substrates were observed. For Ac-RSGK_Prop_IW–OH, when compared to the wild-type enzyme, a 2-fold increase in affinity (*K*_m_ = 410 and 200 μM), a 2.6-fold increase in the specificity constant (*k*_cat_/*K*_m_ = 190 and 500 M^–1^ s^–1^), and a 1.3-fold higher *k*_cat_ (0.077 vs 0.10 s^–1^) was observed (Figure 5C, Table 2). From these changes, we surmised that neutralizing the guanidinium group of R_56_ via installation of a methionine improved substrate recognition for Ac-RSGK_Prop_IW–OH. Subsequent assessment of the kinetics of the R_56_M *Mt-*Sirt mutant with Ac-RSGK_Succ_IW–OH resulted in no detectable product formation at the highest concentration tested. This provided clear evidence that R_56_ is important for binding of Ac-RSGK_Succ_IW–OH, likely due to an ionic interaction between cationic R_56_ and the anionic succinyl group, similar to previous reports for hSIRT5.^44^ Overall, these mutagenesis studies demonstrated that both Y_53_ and R_56_ are important to the function of *Mt*-Sirt and that mutagenesis of the R_56_ residue has the ability to modulate the substrate specificity of the enzyme.

### Product, Substrate Analog, and Mechanism-Based Inhibition of *Mt*-Sirt

We evaluated product inhibition of nicotinamide (NAM), and dead-end inhibition from the substrate analogs adenosine 5′-diphosphoribose (ADPR) and (NADH) as inhibitors of *Mt*-Sirt in the presence of high concentrations of Ac-RSGK_Succ_IW–OH (200 μM) and NAD^+^ (1 mM) (Figure 6A,B and Table 3). NAM was a moderate inhibitor of *Mt*-Sirt with an IC_50_ = 530 ± 42 μM, while ADPR exerted weaker activity (IC_50_ = 3.0 ± 0.2 mM), and NADH resulted in a mere 20% inhibition at a concentration of 5 mM.

**Figure 6:**
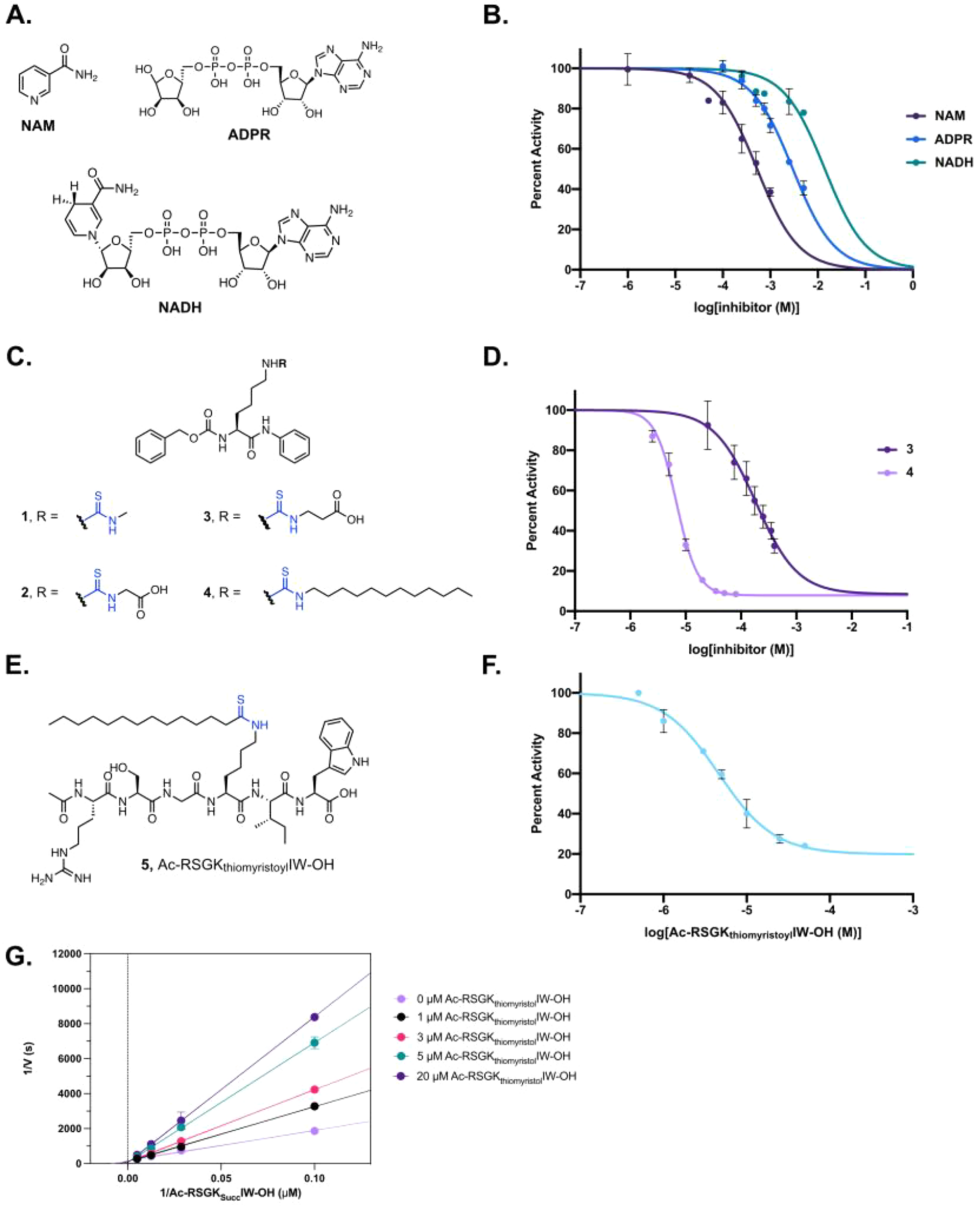
Inhibition of *Mt*-Sirt with NAM, ADPR, NADH, and mechanism-based inhibitors. (A) Structures of NAM, ADPR, and NADH. (B) Residual activity of *Mt*-Sirt observed in the presence of variable concentrations of inhibitor and with fixed NAD^+^ (1 mM) and Ac-RSGK_Succ_IW–OH (200 μM). Data were fitted to eq 7 (C). Structures of thiourea-containing mechanism-based inhibitors used in this study. (D) Residual activity of *Mt*-Sirt observed in the presence of variable concentrations of inhibitor using the same conditions as in (B). (E) Structure of thioamide-containing peptide inhibitor of *Mt*-Sirt. (F) Residual activity of *Mt*-Sirt observed in the presence of 5 using the same conditions as in (B). (G) Double-reciprocal plot of Ac-RSGK_thiomyristoyl_IW–OH vs Ac-RSGKIW–OH at 1 mM NAD^+^. Fitting of data to eq 4 gave values of: *V*_max_ = 0.056 ± 0.004 μM/s, *K*_m_ = 110 ± 20 μM, and *K*_is_ = 4.8 ± 0.6 μM.

**Table 3:** Inhibition Data for Mt-Sirt with Product, Dead-End Inhibitors, and Thioamide-Containing Peptides^a^

**inhibitor**	**IC**_**50**_ **(μM)**
NAM	530 ± 40
ADPR	3000 ± 150
NADH	>5000^b^
**1**	>100^c^
**2**	>100^c^
**3**	150 ± 10
**4**	6.8 ± 0.3
**5**	2.2 ± 0.2

a IC50 values were determined using a 4-parameter fit eq (eq S4).

b The IC50 value could not be determined from the concentration–response curve (20% inhibition at 5 mM).

c We observed less than 10% reduction of activity at 100 μM inhibitor concentration.

We next shifted to developing inhibitors for this enzyme that exploit its catalytic mechanism. Previous work demonstrated that thioamide and thiourea-based inhibitors comprised specific and often potent mechanism-based inhibitors of sirtuins.^52,53^ The putative mechanism of inhibition results from the inability or retardation of the sulfur to undergo the formation of the 1′,2′-cyclic intermediate between the 2′-hydroxyl of the NAD ribose and the thiocarbonyl (Figure 1) of the peptide-derived amide, as has been observed *in crystallo*.^51^

The substitution of the amide carbonyl oxygen with a sulfur is proposed to result in nonideal bond angles for the attack of the 2′-oxygen of the ribose ring onto the 1′-*S*-alkylamidate intermediate when compared to the 1′-*O*-alkylamidate (Figure 1).^54^ Further, the lower electrophilicity of the 1′-*S*-alkylamidate species likely contributes to this diminished rate of reaction.^54^ To exploit this finding, we replaced the N^ε^-acetyl group in the N-carbobenzyloxy-lysine-phenylamide scaffold with N^ε^-thiourea groups (Figure 6). These compounds were synthesized by either preparing the appropriate *tert*-butyl isothiocyanate,^55^ and reacting it with the *ε*-amino group of Cbz-Lys-phenylamide, followed by acid-catalyzed deprotection of the *t*-butyl group to yield the desired thiourea, or by converting the ε-amino group of Cbz-Lys-phenylamide to an isothiocyanate followed by reaction with a primary amine-containing substituent to yield the thiourea.^56^ Neither the thiourea mimic of the propionyl-lysine substrate, compound **1** (Cbz-N^6^-(carbamothioyl)-l-lysine-phenylamide), nor the succinyl-lysine mimic, compound **2** (N^6^-((carboxymethyl)­carbamothioyl)-l-lysine-phenylamide), exhibited significant inhibition of *Mt*-Sirt (<10% reduction of activity at 100 μM). The addition of a methylene group to the thiourea succinyl-lysine mimic, to make a thiourea mimic of the glutaryl-lysine analogue, (**3**, N^6^-((2-carboxyethyl)­carbamothioyl)-l-lysine-phenylamide), resulted in weak inhibition with an IC_50_ = 150 ± 10 μM (Figure 6C,D). Consistent with our analysis of substrates, which indicated that the lowest values of *K*_m_ were observed for the N^ε^-myristoyl- and N^ε^-palmitoyl-containing Ac-RSGK_Acyl_IW–OH, the thiourea analogue (**4**) containing a thiourea mimic of myristoyl-lysine proved to be the most potent inhibitor of the group with an IC_50_ of 6.8 ± 0.3 μM. Incorporation of thiomyristoyl lysine into the ACSK_617_ scaffold (Ac-RSGK_thiomyristoyl_IW–OH, **5**) produced a potent inhibitor of *Mt*-Sirt (IC_50_ = 2.2 ± 0.2 μM, Figure 6F) A plot of Ac-RSGK_thiomyristoyl_IW–OH vs Ac-RSGK_succ_IW–OH in double-reciprocal form (Figure 6G) appeared to conform to competitive inhibition, which upon fitting to eqs 4 and 5 provided the best fitting to eq 4, thereby confirming that it exerted competitive inhibition (*V*_max_ = 0.056 ± 0.004 μM/s, *K*_m_ = 110 ± 20 μM, and *K*_is_ = 4.8 ± 0.6 μM). We also note that the enzyme never fully lost activity with all thioamide and thiourea-containing inhibitors. This residual activity was greater with compound **5**, consistent with findings that enzymatic dethioacylation of the thioamide peptides can occur with NAD^+^-dependent deacylases^57^ and was confirmed by the formation of the dethioacylated product of (**5**) upon incubation of *Mt*-Sirt, NAD^+^, and (**5**) the absence of substrate (data not shown). Together, these data show that chemical probes to target *Mt*-Sirt could be developed to study how inhibition of *Mt*-Sirt activity alters the post-translational protein modification landscape of *Mtb*.

### Kinetic Mechanism of *Mt*-Sirt

Previous studies determined that human sirtuin-2 (hSIRT2) follows a random sequential bi–bi kinetic mechanism in which the free enzyme binds to either the acyl N^ε^-lysinyl-peptide substrate or NAD^+^ to form the first set of central complexes, but has a preference for binding to the peptide substrate first.^58^ In more recent studies of the hSIRT2 homologue hSIRT6, it has been suggested that NAD^+^ binds first to the enzyme,^59^ in variance with the mechanism of hSIRT2. As *Mt*-Sirt has structural similarity to hSIRT5 and hSIRT2 and functional similarity to hSIRT5 and hSIRT6, we sought to analyze its kinetic mechanism. As Ac-RSGK_Succ_IW–OH was the most catalytically efficient substrate in our survey, we utilized this as the peptide substrate to conduct initial velocity studies with *Mt*-Sirt. Initial velocity data of Ac-RSGK_Succ_IW–OH (10–200 μM) vs NAD^+^ (50–800 μM), when plotted in double-reciprocal form, conformed to an intersecting pattern in which the changing-fixed substrate (Ac-RSGK_Succ_IW–OH) exerted both slope and intercept effects, thereby indicating a sequential kinetic mechanism (Figure 7A).

**Figure 7:**
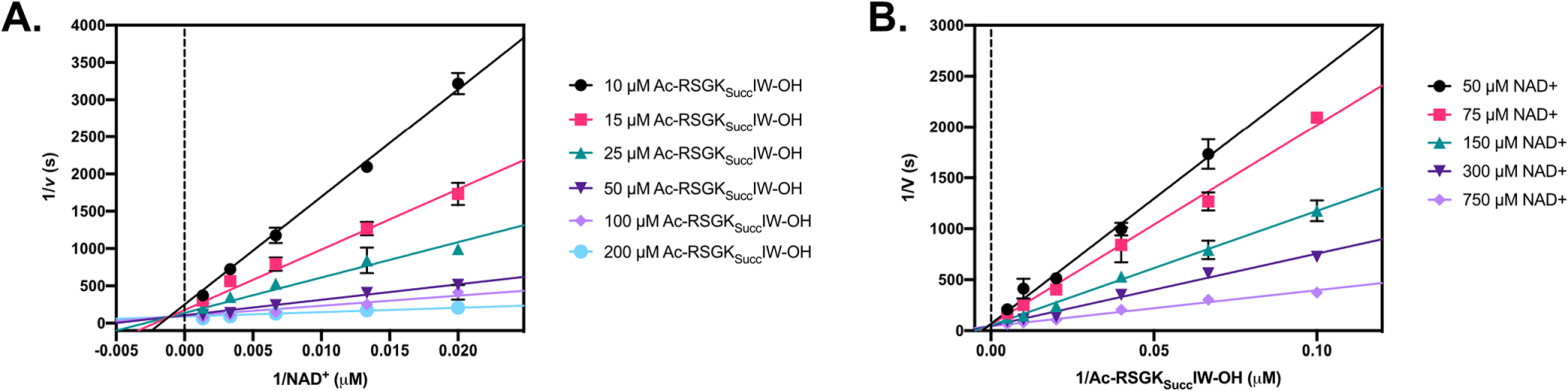
Initial velocity study of *Mt*-Sirt. (A) Double-reciprocal plot of 1/*v* vs 1/NAD^+^ (50–800 μM) at changing-fixed concentration of Ac-RSGK_Succ_IW–OH (10–200 μM), with lines drawn through the experimental data from fitting to eq 3. (B) Double-reciprocal plot of 1/*v* vs 1/Ac-RSGK_Succ_IW–OH (10–200 μM) at changing-fixed NAD^+^(50–800 μM), with lines drawn through the experimental data from fitting to eq 3.

When the initial velocity data were plotted as NAD^+^ vs Ac-RSGK_Succ_IW–OH (Figure 7B), the double-reciprocal plot was intersecting, and all lines converged on the y-intercept, which indicates an equilibrium-ordered kinetic mechanism in which NAD^+^ binds in rapid equilibrium before the binding of the peptide substrate.

We fitted the initial velocity data in Figure 7 to both eq 2 (sequential bi bi mechanism) and eq 3 (equilibrium-ordered mechanism). Although both fits gave equal values of *R* ^2^ in the linear regression, from fitting to eq 2, a value for *K*_a_ = 20 ± 50 μM was obtained, indicating a better overall fit to eq 3. The kinetic parameters obtained from fitting to 3 were: *V*_max_ = 0.0133 ± 0.0008 μM/sec, *K*_iNAD+_ = 1300 ± 400 μM, *K*_Ac‑RSGKSuccIW–OH_ = 30 ± 9 μM (Table 4). The ordered mechanism observed for *Mt*-Sirt in which the N^ε^-acyl-lysinyl peptide substrate binds after binding of NAD^+^ is in agreement with the ordered kinetic mechanism of hSIRT6, although it was unclear in the previous study with hSIRT6 whether or not the binding of NAD^+^ was in rapid equilibrium.^60^ A consequence of this equilibrium-ordered mechanism is that only a suitable N^ε^-acyl-lysinyl residue within a protein or peptide substrate is able to “lock” NAD^+^ onto *Mt-*Sirt and initiate catalysis, thereby avoiding the binding and hydrolysis of inappropriate substrates.^47^ Such a mechanism imbues high selectivity for specific N^ε^-acyl-lysinyl substrates as would seem appropriate for an enzyme involved in complex cellular signaling.

**Table 4:** Initial Velocity, Product, and Dead-End Inhibition Studies of Mt-Sirt^a^

**initial velocity study**							
**variable substrate**	**changing-fixed substrate**	**pattern type**	***K***_**iNAD+**_ **(μM)**	***K***_**NAD+**_ **(μM)**	***K***_**Ac‑RSGKSuccIW–OH**_ **(μM)**	***V***_**max**_ **(μM/s)**	**eqn fitted**
Ac-RSGK_Succ_IW–OH	NAD^+^	Equilibrium-Ordered Intersecting on 1/*v* -axis	1300 ± 400		30 ± 9	0.0133 ± 0.0008	3

**product inhibition studies**							
**variable substrate**	**inhibitor**	**fixed substrate**	**inhibition pattern**	**app** ***K***_**m**_ **(μM)**	***K***_**is**_ **(μM)**	***K**_**ii**_* **(μM)**	**eqn fitted**
NAD^+^	NAM	Ac-RSGK_Succ_IW–OH (0.2 mM)	NC	240 ± 20	210 ± 20	500 ± 100	5
Ac-RSGK_Succ_IW–OH	NAM	NAD^+^ (1 mM)	C	57 ± 5	60 ± 5		4; r^2^ = 0.9912
Ac-RSGK_Succ_IW–OH	NAM	NAD^+^ (1 mM)	NC	62 ± 5	85 ± 10	600 ± 200	5; r^2^ = 0.9935

**dead-end inhibition studies**							
**variable substrate**	**inhibitor**	**fixed substrate**	**inhibition pattern**	**app** ***K***_**m**_ **(μM)**	***K***_**is**_ **(μM)**	***K**_**ii**_* **(μM)**	**eqn fitted**
NAD^+^	ADPR	Ac-RSGK_Succ_IW–OH (0.2 mM)	NC	260 ± 20	250 ± 20	1400 ± 100	5
Ac-RSGK_Succ_IW–OH	ADPR	NAD^+^ (1 mM)	NC	46 ± 4	1800 ± 400	2600 ± 400	5
NAD^+^	Cbz-Lys-AMC	Ac-RSGK_Succ_IW–OH (0.04 mM)	UC	770 ± 90		1400 ± 200	6
NAD^+^	Cbz-Lys-AMC	Ac-RSGK_Succ_IW–OH (0.2 mM)	UC	120 ± 10		8000 ± 2000	6
Ac-RSGK_Succ_IW–OH	Cbz-Lys-AMC	NAD^+^ (1 mM)	C	140 ± 10	6000 ± 1000		4

a pH 7.5; C, NC, UC; competitive, noncompetitive (mixed), and uncompetitive inhibition, app *K*_m_, apparent Michaelis constant; *K*_is_, and *K_ii_*, slope and intercept inhibition constants.

We next performed product and dead-end inhibition studies using nicotinamide (NAM), adenosine diphosphate ribose (ADPR), and Cbz-Lys-AMC (Figure 8 and Table 4). Previous studies of sirtuins have shown that NAM can undergo a partial reverse reaction with the ADPR-peptide alkylimidate intermediate and regenerate both NAD^+^ and peptide substrates, thus effectively inhibiting catalysis of the deacylase half-reaction.^60^ NAM has also been ubiquitously observed to be the first product released from the enzyme,^4–7,61^ which would result in noncompetitive inhibition by NAM vs both substrates. The double reciprocal plot of 1/*v* vs 1/[NAD^+^] with changing-fixed concentrations of NAM and a high fixed concentration of 200 μM Ac-RSGK_Succ_IW–OH was a series of lines intersecting to the left of the 1/*v*-axis, indicative of noncompetitive (mixed) inhibition, as was observed for other sirtuin-like enzymes (Figure 8A).^7,62^ Fitting of the data to eq 5 resulted in kinetic parameters of *K*_NAD+_ = 240 ± 20 μM, *K*_is_ = 210 ± 20 μM and *K_ii_* = 500 ± 100 μM (Table 4). This noncompetitive inhibition would result from binding of NAM to an enzyme–product or enzyme-intermediate complex, and a slope effect would also result from binding of NAM to free enzyme to which its precursor, NAD^+^, binds. The double-reciprocal plot of 1/*v* versus 1/[Ac-RSGK_Succ_IW–OH] with changing-fixed concentrations of NAM and a high fixed level of NAD^+^ (1 mM) also resulted in a series of lines that intersected slightly to the left of the 1/*v*-axis, consistent with noncompetitive inhibition (Figure 8B). Fitting of these data to both eqs 4 and 5 led to a slightly better fit to eq 5, with kinetic parameters of *K*_Ac‑RSGKSuccIW–OH_ = 62 ± 5 μM, *K*_is_ = 80 ± 10 μM, and *K_ii_* = 600 ± 200 μM (Table 4).

**Figure 8:**
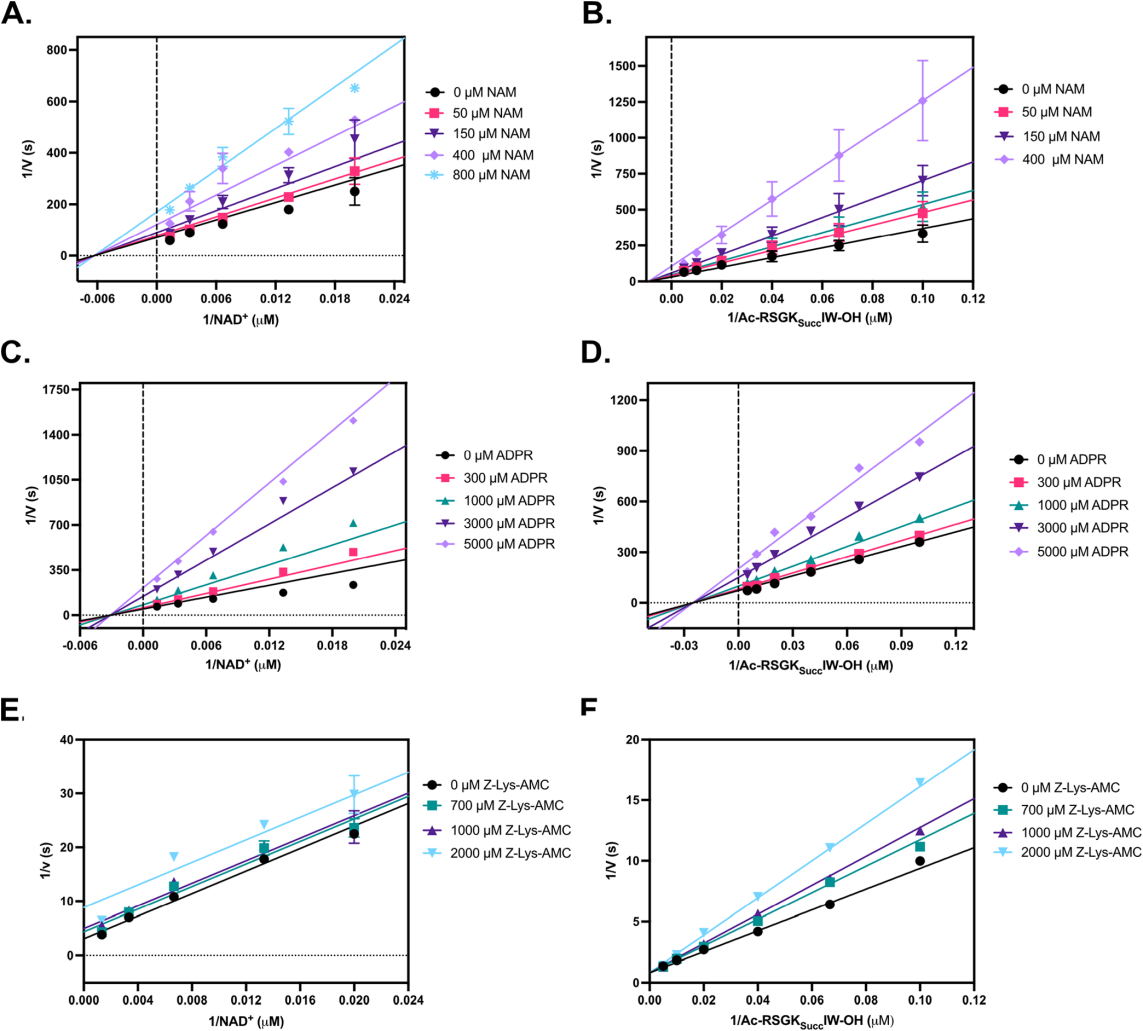
Inhibition of *Mt*-Sirt by product (NAM) and dead-end inhibitors (ADPR) and Cbz-Lys-AMC vs both substrates. The lines drawn through the experimental data points in the six double-reciprocal plots were from fitting of the data to either eqs 4, 5, or 6. (A) Plot of 1/*v* vs1/[NAD^+^] (50–1000 μM) at changing-fixed concentrations of NAM (0–800 μM) and 200 μM of Ac-RSGKSuccIW–OH, with data derived from fitting of the data to eq 5. (B) Plot of 1/*v* vs1/[Ac-RSGKSuccIW–OH] (10–200 μM) at changing-fixed concentrations of NAM (0–400 μM) and 1000 μM of NAD^+^, with data derived from fitting to eq 4. (C) Plot of 1/*v* vs1/[NAD^+^] (50–1000 μM) at changing-fixed concentrations of ADPR (0–5000 μM) and 1 mM of NAD^+^, with data derived from fitting to eq 5. (D) Plot of 1/*v* vs1/[Ac-RSGKSuccIW–OH] (10–200 μM) at changing-fixed concentrations of ADPR (0–5000 μM) and 1000 μM of NAD^+^, with data derived from fitting to eq 5. (E) Plot of 1/*v* vs 1/[NAD^+^] (50–750 μM) at changing-fixed concentrations of Cbz-Lys-AMC (0–2000 μM) and 40 μM of Ac-RSGKSuccIW–OH, with data derived from fitting to eq 6. (F) Plot of 1/*v* vs1/[Ac-RSGKSuccIW–OH] (10–200 μM) at changing-fixed concentrations of Cbz-Lys-AMC (0–2000 μM) and 1000 μM of NAD^+^, with data derived from fitting to eq 4.

ADPR structurally resembles both the 2′-*O*-acyl-ADPR product and NAD^+^, so that one may expect its binding to free enzyme and to an enzyme–product complex. ADPR was a noncompetitive inhibitor vs NAD^+^ (*K*_NAD+_ = 260 ± 20 μM, *K*_is_ = 250 ± 20 μM and *K_ii_* = 1400 ± 100 μM; Figure 8C and Table 4) and a noncompetitive inhibitor vs Ac-RSGK_Succ_IW–OH (*K*_Ac‑RSGKSuccIW–OH_ = 47 ± 6 μM, *K*_is_ = 500 ± 20 μM and *K_ii_* = 2200 ± 100 μM; Figure 8D and Table 4). The noncompetitive inhibition vs both substrates suggested that ADPR binds to two enzyme forms, and we propose these to be free enzyme and an enzyme–product complex.

The peptide substrate analogue, Cbz-Lys-AMC, was an apparent competitive inhibitor vs Ac-RSGK_Succ_IW–OH at 1 mM NAD^+^ (Figure 8F), which upon fitting to eq 4 gave kinetic parameters of *K*_Ac‑RSGKSuccIW–OH_ = 140 ± 100 μM and *K*_is_ = 6000 ± 1000 μM, indicating that this dead-end inhibitor binds to the E-NAD^+^ enzyme form. When NAD^+^ was the variable substrate at two fixed concentrations of Ac-RSGK_Succ_IW–OH (0.04 and 0.2 mM), the two double-reciprocal plots were comprised of a series of parallel lines, indicative of uncompetitive inhibition (Figures 8E and S5G,H). Fitting of the data to eq 6 resulted in kinetic parameters *K*_NAD+_ = 760 ± 70 μM and *K_ii_* = 1600 ± 200 (0.04 mM Ac-RSGK_Succ_IW–OH) and *K*_NAD+_ = 120 ± 10 μM and *K_ii_* = 8000 ± 2000 (0.2 mM Ac-RSGK_Succ_IW–OH). The uncompetitive inhibition is consistent with an ordered mechanism in which NAD^+^ binds before Ac-RSGK_Succ_IW–OH, which again supports the equilibrium-ordered mechanism. Also, for the two Cbz-Lys-AMC vs NAD^+^ plots, the increase in the value of *K_ii_* at the higher concentration of fixed Ac-RSGK_Succ_IW–OH was as expected because Cbz-Lys-AMC is a competitive inhibitor of Ac-RSGK_Succ_IW–OH. While we are unable to ascertain the order, or lack thereof, of the products of Mt-Sirt, we may conclude that the binding of substrates conforms to an equilibrium-ordered mechanism in which NAD^+^ binds in rapid equilibrium prior to the binding of Ac-RSGK_Succ_IW–OH.

### N^ε^-Succinylated *Mt*ICL1 is a Substrate of *Mt*-Sirt and an *Mt*ICL1 Succinylation Mimic Exhibits Altered Loop Closure

We next sought to determine if *Mt*-Sirt can catalyze desuccinylation of a key *Mtb* enzyme that is proposed to undergo lysine succinylation. Proteomic studies demonstrated that lysines 189, 322, and 334 of isocitrate lyase-1 (ICL1) of *Mtb*, an essential enzyme for persistent infection of hosts by *Mtb*,^63,64^ are subject to N^ε^-succinylation.^15,65^ It is worth noting that since succinate is a product of MtICL1 catalysis, it is sensible that N^ε^-succinylation via succinyl-CoA could be a possible mechanism for its regulation. This concept is further exemplified by structural and CRISPRi data showing that a transcriptional regulator of *Mt*ICL1, *Mt*RamB, binds to succinyl-CoA and locks it in a transcriptionally inactive conformation, which reduces *icl1* transcript levels,^66^ suggesting *Mt*ICL1 activity may be regulated by succinyl-CoA both transcriptionally and posttranslationally. To assess the relevance of these lysine PTMs on *Mt*ICL1 activity, we prepared mimics of N^ε^-succinylation (Lys to Glu mutation) for K_189_, K_322_, and K_334_ of the enzyme. Consistent with previous findings,^16^ K_322_E and K_334_E resulted in minor alterations of enzyme activity (Figure 9B and S6). However, *Mt*ICL1 K_189_E mutation resulted in a near-total loss of enzymatic activity compared to wild-type (*k*_cat_/*K*_m_ = 9.5 ± 0.3 M^–1^ s^–1^ versus (4.3 ± 0.2) × 10^5^ M^–1^ s^–1^) (Figure S6). These data suggested that the succinylation of lysine 189 of *Mt*ICL1 could serve to modulate metabolic flux through the glyoxylate shunt, a critical pathway for the persistence of *Mtb* in the host,^64^ and thus removal of such PTMs by *Mt*-Sirt could play an important role in *Mtb* metabolism.

**Figure 9:**
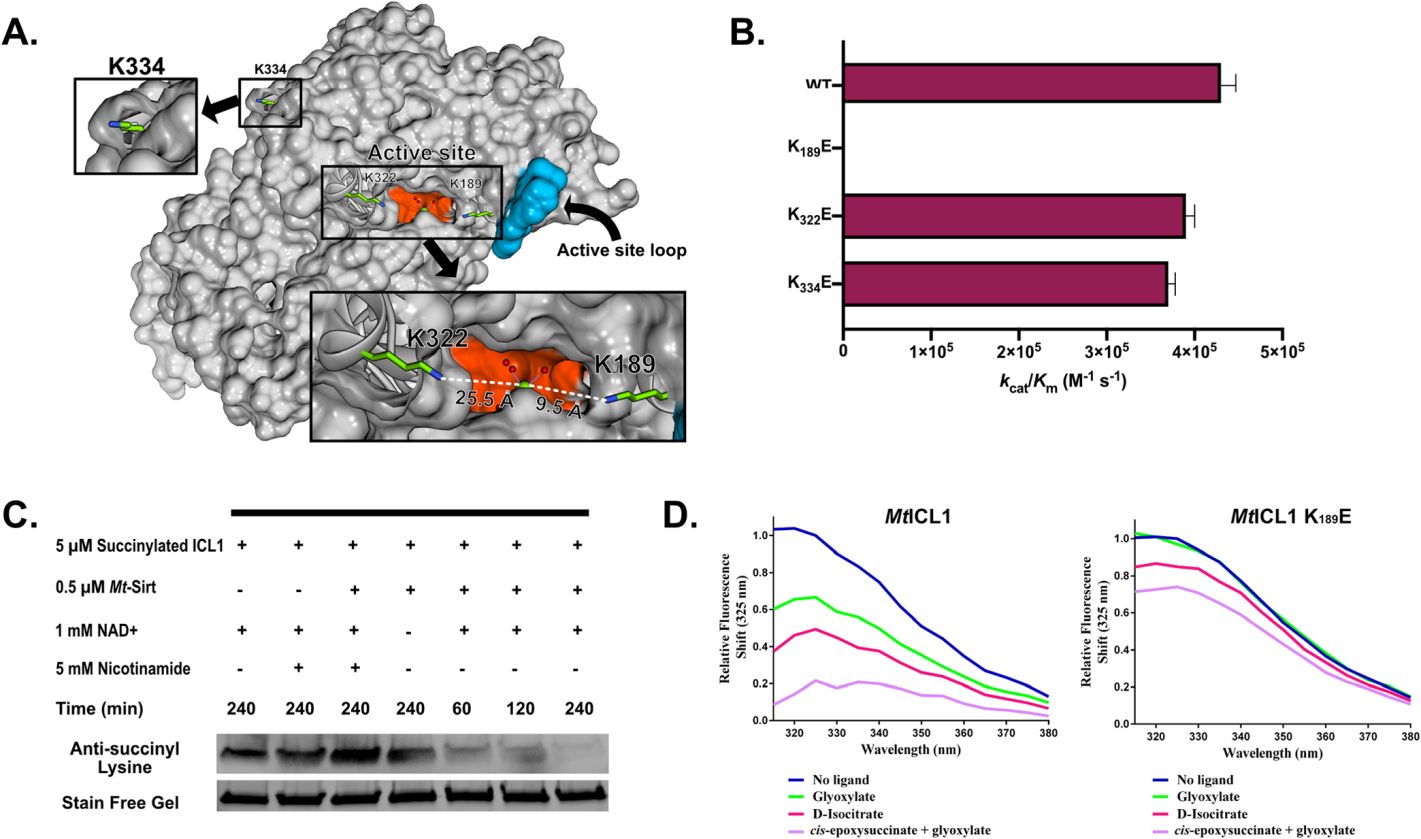
Succinylated *Mt*ICL1 is a substrate of *Mt*-Sirt and succinyl lysine mimics of *Mt*ICL1 exhibit altered kinetic behavior. (A) Surface view of *Mt*ICL1 shown as a monomer (PDB: 1F61). Sites of lysine succinylation identified in proteomic studies are shown in yellow, of which one, K189, resides in the active site loop (blue). Zoom-in view of the active site shows the distances between the N^ε^-amine of K189 and K322 and the bound active-site Mg^2+^ (orange). (B) Second-order rate constants (*k*_cat_/*K*_m_) as determined by steady-state analysis of *Mt*ICL1 lysine to glutamate succinyl mimics. (C) Western blot demonstrating desuccinylation of lysine residues from chemically succinylated *Mt*ICL1 by *Mt*-Sirt. (D) Intrinsic tryptophan fluorescence of *Mt*ICL1 (1 μM) and *Mt*ICL1 K189E (1 μM) with added glyoxylate (1 mM), D-isocitrate (1 mM), and the *Mt*ICL1 covalent inactivator *cis*-epoxysuccinate (100 μM) in the presence of glyoxylate (1 mM).

Accordingly, we treated recombinant *Mt*ICL1 with variable concentrations of succinyl-CoA. We observed concentration-dependent succinylation of the protein as determined via Western blotting using an antisuccinyl lysine antibody (Figure S7A). Mass spectrometric analysis of *Mt*ICL1 tryptic peptides following succinyl-CoA labeling identified succinylation of K_189/190_, K_322_, K_331_, and K_334_ (Figure S7B), consistent with modifications observed in the proteomic literature, and suggested that these modified lysine residues may exhibit enhanced reactivity and/or facile accessibility on the protein surface.^67^ Subsequently, incubation of modified *Mt*ICL1 with *Mt*-Sirt resulted in nearly complete removal of the succinyl groups, as observed by Western blotting, confirming that *Mt*ICL1 is a substrate of *Mt*-*Sirt* (Figure 9C).

As *Mt*ICL1 is a substrate of *Mt*-Sirt, we sought to better understand the mechanism behind the significant loss of activity for the *Mt*ICL1 K_189_E mimic of succinylation. We posited that the net charge change of two, induced by this mutation, may result in altered loop closure of the enzyme active site loop^19,68^ thereby leading to the observed loss in enzyme activity. To assess this, we employed intrinsic tryptophan fluorescence to monitor the closure of the *Mt*ICL1 active-site loop, which contains its catalytic Cys_191_ residue.^37,38,69^ Incubation of wild-type *Mt*ICL1 in the presence and absence of (co)­substrates (glyoxylate and D-isocitrate) and a known uncompetitive covalent inactivator of *Mt*ICL1 (*cis*-epoxysuccinate (*cis*-EpS))^37^ in the presence of glyoxylate resulted in significant fluorescent quenching, consistent with movement such as closure of this loop (Figure 9D). Conversely, under the same conditions, the *Mt*ICL1 K_189_E mutant showed negligible quenching, consistent with diminution of movement of this loop. Further, differential scanning fluorimetry of both *Mt*ICL1 K_189_E and wild-type *Mt*ICL1 in the presence of glyoxylate and *cis*-EpS plus glyoxylate revealed negligible thermal stabilization for the *Mt*ICL1 K_189_E mutant (0 and 2 °C, respectively) whereas significant stabilization of wild-type *Mt*ICL1 (5 and 22 °C, respectively) was observed (Figure S8A,B). To ensure the reduction in fluorescence quenching and loss in thermal stability of *Mt*ICL1 K_189_E was not due to a loss in substrate binding, we employed isothermal calorimetry and observed clear saturatable isotherms for the binding of glyoxylate to both K_189_E and wild-type *Mt*ICL, consistent with glyoxylate binding to both enzymes (Figure S8C,D). Together, these data suggested that loop closure of *Mt*ICL1 K_189_E, not substrate binding, is impeded by a mimetic of lysine succinylation. Therefore, if lysine succinylation of *Mt*ICL1 K_189_ occurs at a reasonable stoichiometry in *Mtb*, it could, in concert with *Mt*-Sirt, modulate *Mt*ICL1 enzymatic activity. Looking beyond *Mt*ICL1, there are other reported substrates of *Mt*-Sirt as well as acyltransferase enzymes that can carry out lysine succinylation, such as Rv0802c.^70,71^

### Fatty Acid Acylation of Proteins Occurs in the *Mtb* Proteome

Given that we identified that *Mt*-Sirt exerts deacylase activity on lysine residues containing N-fatty acyl groups, we sought to determine the extent of fatty acid protein modification in the *Mtb* proteome. Therefore, we cultured an attenuated *Mtb* strain (mc^2^7000)^72,73^ in the presence of Alk-12, an alkynyl analog of myristic acid (Figure 10A).^36^ Upon lysis, we conducted bio-orthogonal (click) labeling with a Cy7-azide to the alkyne-labeled target proteins. Following labeling, we treated the samples with hydroxylamine to remove thioester linkages to cysteine residues, leaving only, in theory, residues of proteins modified with an amide bond (N^ε^-lysine and N-terminal glycine myristoylation) (Figure 10A).^48,74^ Resolving the samples by SDS-PAGE and assessing in-gel fluorescence, we identified an abundance of Alk-12-modified proteins that exist in the *Mtb* proteome (Figure 10B); thus, suggesting that long-chain fatty acid modification of *Mtb* proteins and the deacylase activity of *Mt*-Sirt may play relevant and yet uncharacterized intracellular roles.

**Figure 10:**
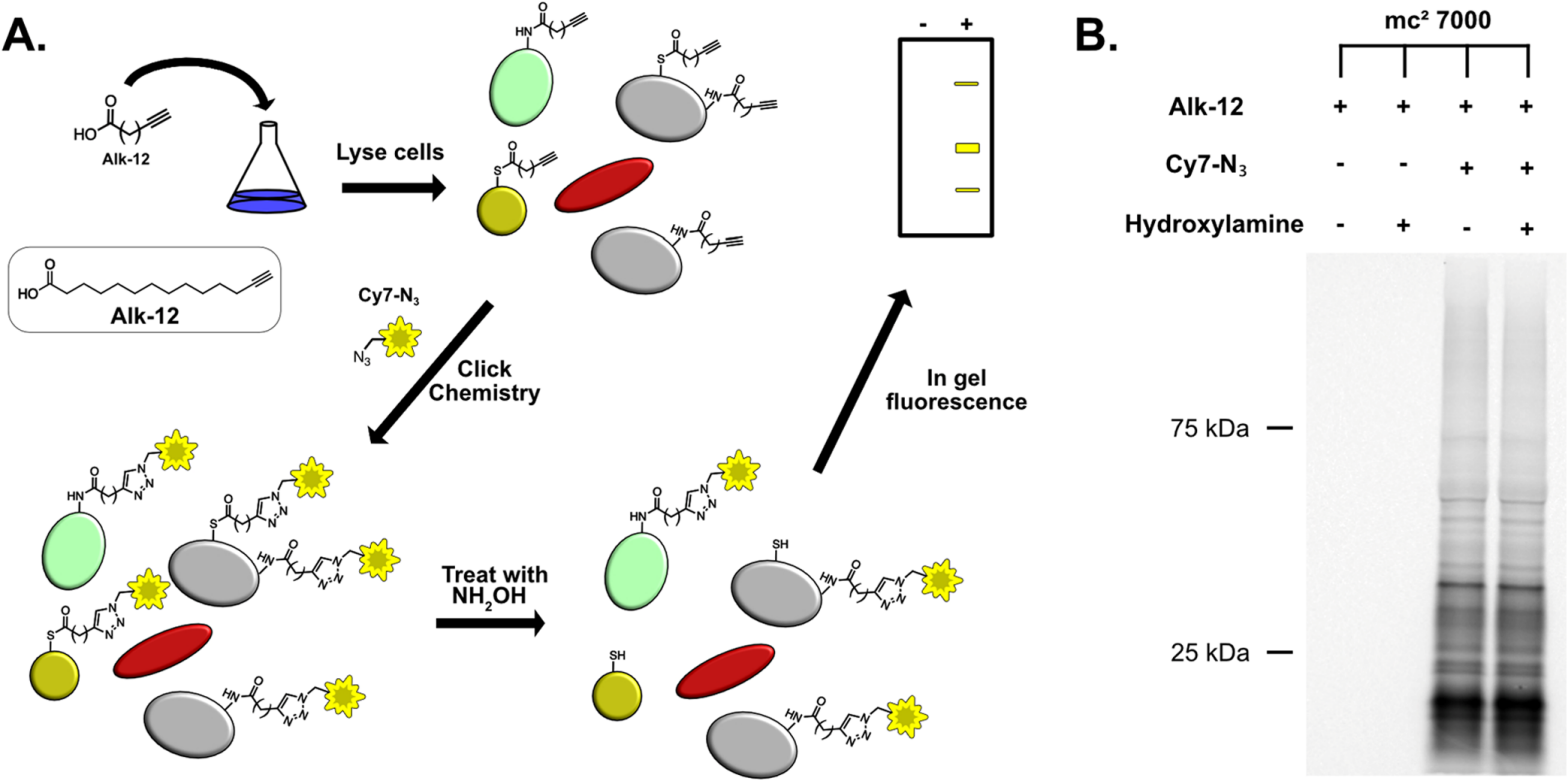
*Mtb* proteins are labeled with the fatty acid metabolic probe Alk-12. (A) Overview of experimental workflow employed to assess Alk-12 fatty acylation in *Mtb*. (B) In-gel fluorescence of Cy7-labeled proteins that are covalently modified by Alk-12, consistent with the fatty acylation of these proteins.

## Conclusions

Through a combination of sequence alignment and homology modeling, we propose that *Mt*-Sirt, the sole Sir2-like lysine deacylase of *Mtb*, may exert desuccinylase activity of N^ε^-succinyl-lysyl peptides. Synthesis and steady-state kinetic characterization of Cbz-N^ε^-acyl-lysinyl-AMC and a panel of N^ε^-acyl-lysinyl-containing hexapeptide substrates demonstrated that N^ε^-succinyl-lysine-containing substrates were the most active of those evaluated, and the unexpected finding that N^ε^-acyl-lysinyl-containing hexapeptides in which the acyl group was either myristoyl or palmitoyl comprised highly efficient substrates. Mutational analysis of *Mt*-Sirt identified that R_56_ is essential for the desuccinylase activity of the enzyme, but not for efficient deacylation of short alkyl chain substrates. Intriguingly, the Y_53_F mutation impacts the activity of the enzyme for both succinyl and propionyl substrates, suggesting its phenol functionality may perturb the conformation of key active-site residues required for efficient substrate binding and catalysis. Analysis of known sirtuin inhibitors nicotinamide and adenosine 5′-diphosphoribose resulted in moderate inhibition of the enzyme, while NADH was a poor inhibitor. However, preparation of mechanism-based N^ε^-acyl thiourea and thioamide analogs provided more potent inhibitors of *Mt-*Sirt (IC_50_ = 2.2–6.8 μM). We conducted kinetic analysis of *Mt*-Sirt, which produced plots consistent with an equilibrium-ordered kinetic mechanism in which NAD^+^ binds first to the enzyme in rapid equilibrium, followed by the N-acylated peptide. We demonstrated that *Mt*-Sirt can catalyze desuccinylation of a known succinylated protein, *Mtb* ICL1. To better understand the consequences of *Mt*ICL1 lysine succinylation, we assessed *Mt*ICL1 lysine succinylation mimetics (Lys to Glu) and showed the marked reduction in activity of a K_189_E mimic likely arises from ineffective active-site loop closure. We finally sought to initially assess the abundance of fatty acid-acyl modified lysine residues in the *Mtb* proteome by using the metabolic label Alk-12, an alkynyl analog of myristic acid. Using this probe, we demonstrated that extensive N^ε^-myristolyation of *Mtb* proteins occurred, suggesting that the deacylase activity of *Mt*-Sirt on longer-chain acyl groups may play as-yet uncharacterized roles in *M. tuberculosis*.

## Supplementary Material



## Notes

Experimental procedures for synthetic chemistry, including NMR data, LC-MS traces, equations for data fitting, additional enzymatic data, including kinetic mechanisms data, biophysical data, and supplementary methods (PDF)

## †

Department of Biological Chemistry & Molecular Pharmacology, The Blavatnik Institute, Harvard Medical School, 250 Longwood Ave, Boston, Massachusetts 02115, United States

## Notes

This work is dedicated to the memory of Prof. John S. Blanchard.

## Abbreviations

ACN: acetonitrile
ACS: acetyl CoA synthetase
ADPR: adenosine diphosphate ribose
AMC: 7-amino-4-methyl-coumarin
BSA: bovine serum albumin
Cbz: carbobenzyloxy
oA: coenzyme A
DCM: dichloromethane
DIEA: *N*,*N*-diisopropylethylamine
DMF: *N*,*N*-dimethylformamide
DMSO: dimethyl sulfoxide
DTT: dithiothreitol
ESI: electro-spray ionization
ECL: electrochemiluminescence
Fmoc: 9-fluorenylmethoxycarbonyl
Hepes: (4-(2-hydroxyethyl)-1-piperazineethanesulfonic acid)
HPLC-MS: high-performance liquid chromatography–mass spectrometry
HRP: horse radish peroxidase
*Mtb*: *Mycobacterium tuberculosis*
Mtt: methyltrityl
NAD^+^: nicotinamide adenine dinucleotide (oxidized)
NADH: nicotinamide adenine dinucleotide (reduced)
NAM: nicotinamide
PTM: post-translational modification
PVDF: polyvinylidene fluoride
SDS-PAGE: sodium dodecyl sulfate polyacrylamide gel electrophoresis
Sirt: sirtuin
TFA: trifluoroacetic acid
Tris: tris­(hydroxymethyl)­aminomethane
