# Supplementary material for: Substrate Specificity and Kinetic Mechanism of the NAD+‑Dependent Deacylase from Mycobacterium tuberculosis, Rv1151c (Mt-Sirt)
Source: Biochemistry. 2026 Jul 13;65(15):2400–18. doi: 10.1021/acs.biochem.6c00374 (PMC13445592; doi:10.1021/acs.biochem.6c00374)
Supplement: Supplementary file 1 [file bi6c00374_si_001.pdf]

Supplementary Information

Substrate Specificity and Kinetic Mechanism of the NAD$^+$ Dependent Deacylase from *Mycobacterium tuberculosis*, Rv1151c (*Mt*-Sirt)

Drake M. Mellott,[a] Jiyun Zhu,[a,b] Hudson Fulcher,[a] and Thomas D. Meek[a,*]

[a]Department of Biochemistry and Biophysics, Texas A&M University, College Station, TX 77843, United States

[b]Current Address:
Department of Biological Chemistry & Molecular Pharmacology, The Blavatnik Institute, Harvard Medical School, 250 Longwood Ave, Boston, Massachusetts 02115, United States

*Corresponding author

Supplementary Figures……………………………………………………………………S2-9

Supplementary methods…………………………………………………………………S10-12

Synthetic methods………………………………………………………………………S13-21

LC-MS spectra……………………………………………………………………………S22-40

NMR spectra………………………………………………………………………………S41-50

References…………………………………………………………………………………S51

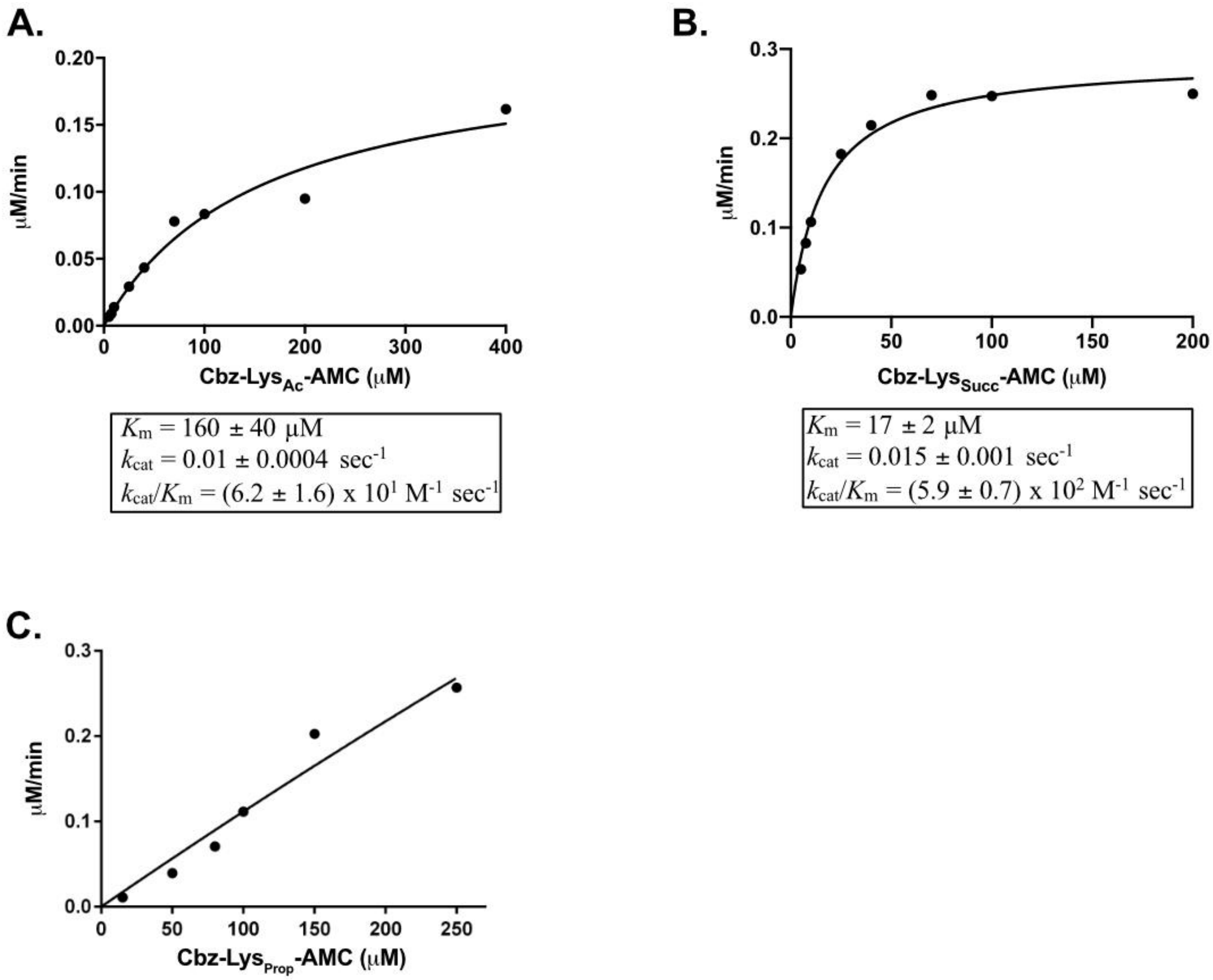

Figure S1. Michaelis-Menten kinetics of Cbz-Lys-AMC substrates. Kinetic parameters of Cbz-Lys$_{Ac}$-AMC (**A**) and Cbz-Lys$_{Succ}$-AMC (**B**) obtained from fitting data to eq. 1. **C**. Kinetics of Cbz-Lys$_{Prop}$-AMC fit to y = mx + b where k_{cat}/K_m = 17 ± 2 M^{-1} sec^{-1}

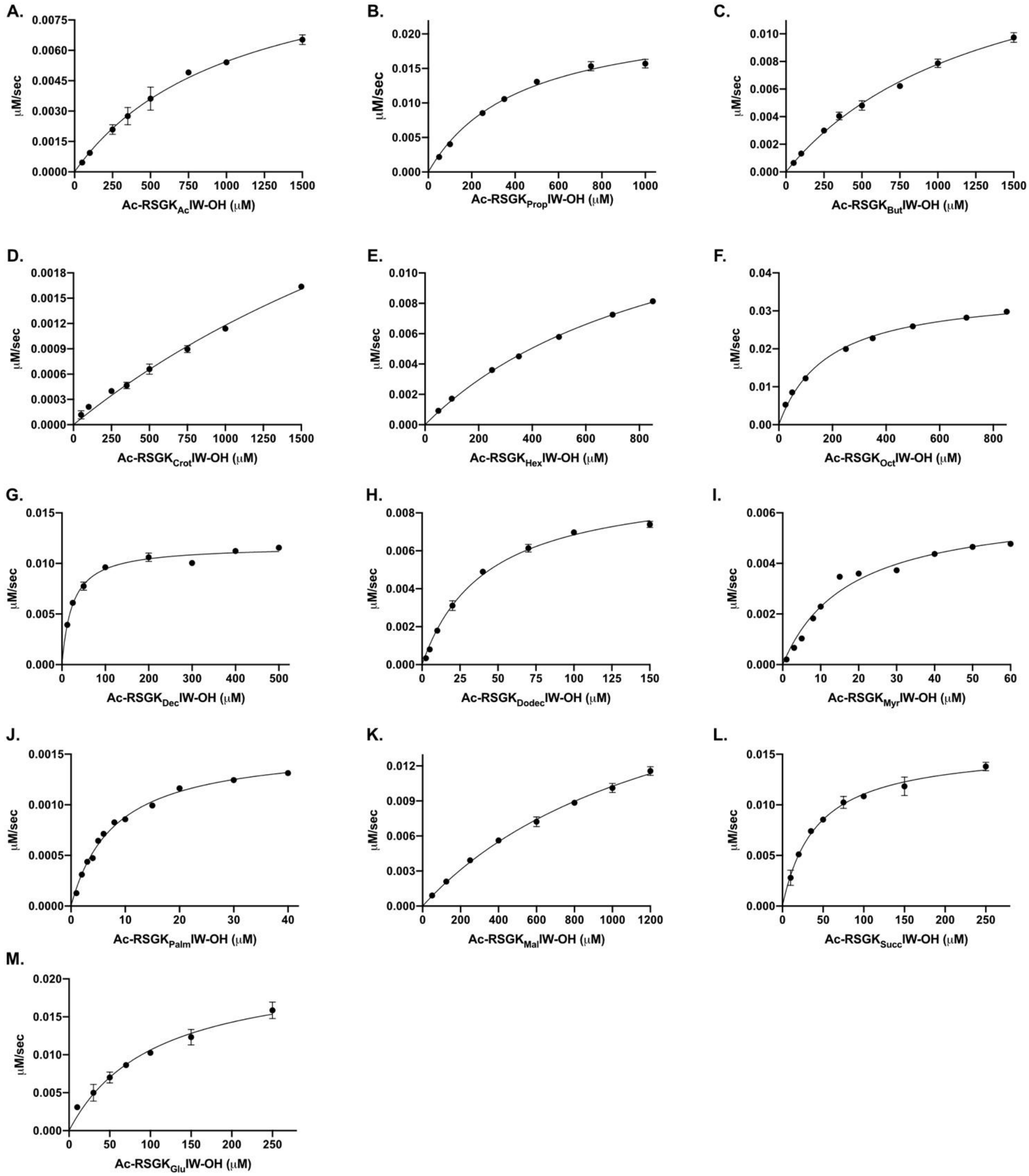

Figure S2. Michaelis-Menten kinetics of *Mt*-Sirt-catalyzed de-acylation Ac-RSGK$_{R}$IW-OH peptides with a fixed concentration of 1mM NAD^{+}. All data were fit to Eq. 1 and all values from these experiments are reported in Table 1.

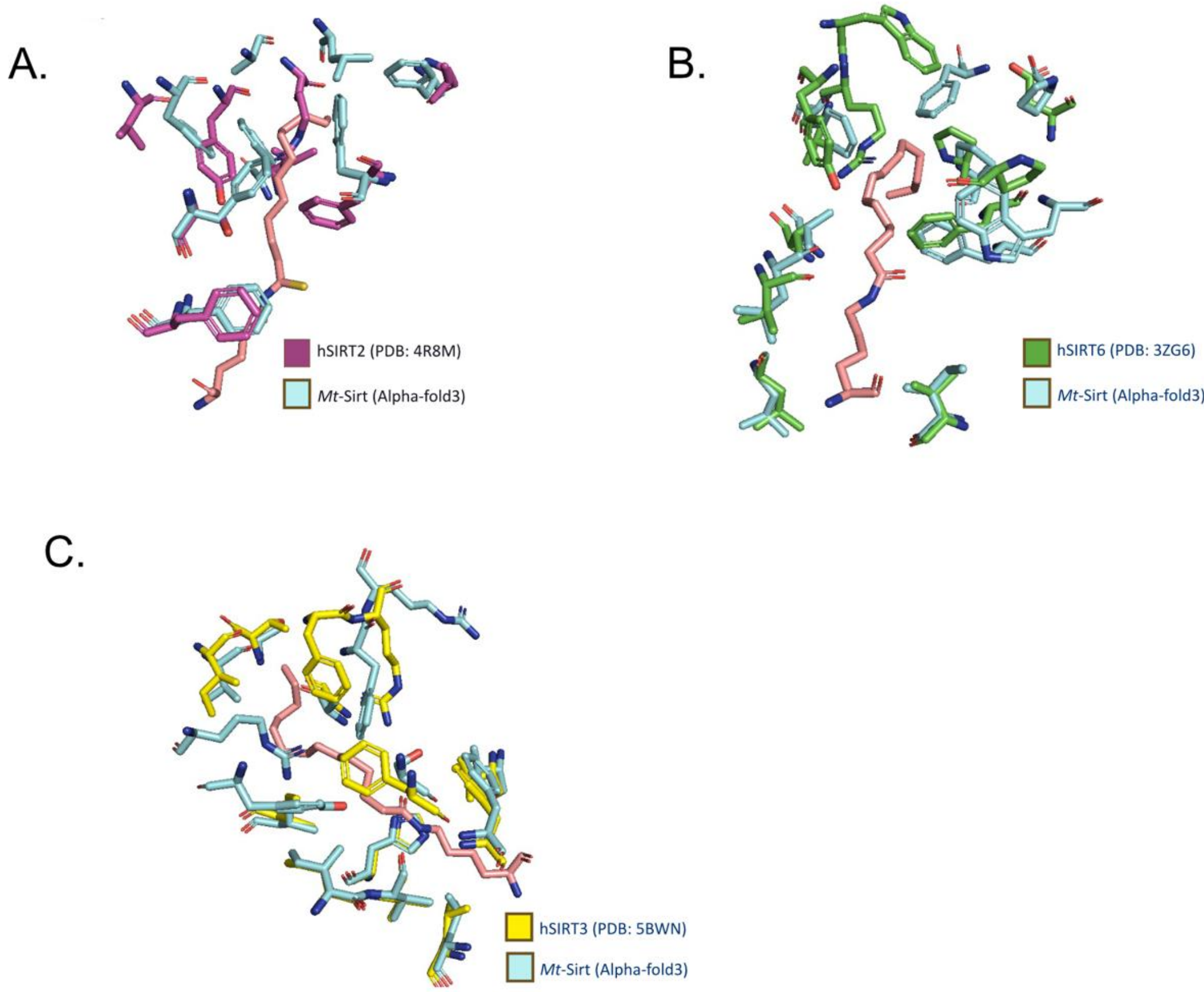

Figure S3. A. Mt-Sirt Alphafold3 model overlaid with hSIRT2 (PDB: 4R8M) bound to a peptide with an N^{e}-myristoyl-lysine residue. Stick display of key residues shown herein and the myristic acid portion of the lysine is shown in salmon. B. Mt-Sirt Alphafold3 model hSIRT6 (PDB: 3ZG6) bound to a peptide with an N^{e}-myristoyl-lysine residue. Stick display of key residues shown herein and the myristic acid portion of the lysine is shown in salmon. C. Mt-Sirt Alphafold3 model hSIRT3 (PDB: 5BWN) bound to a peptide with an N^{e}-myristoyl-lysine residue. Stick display of key residues shown herein and the myristic acid portion of the lysine is shown in salmon.

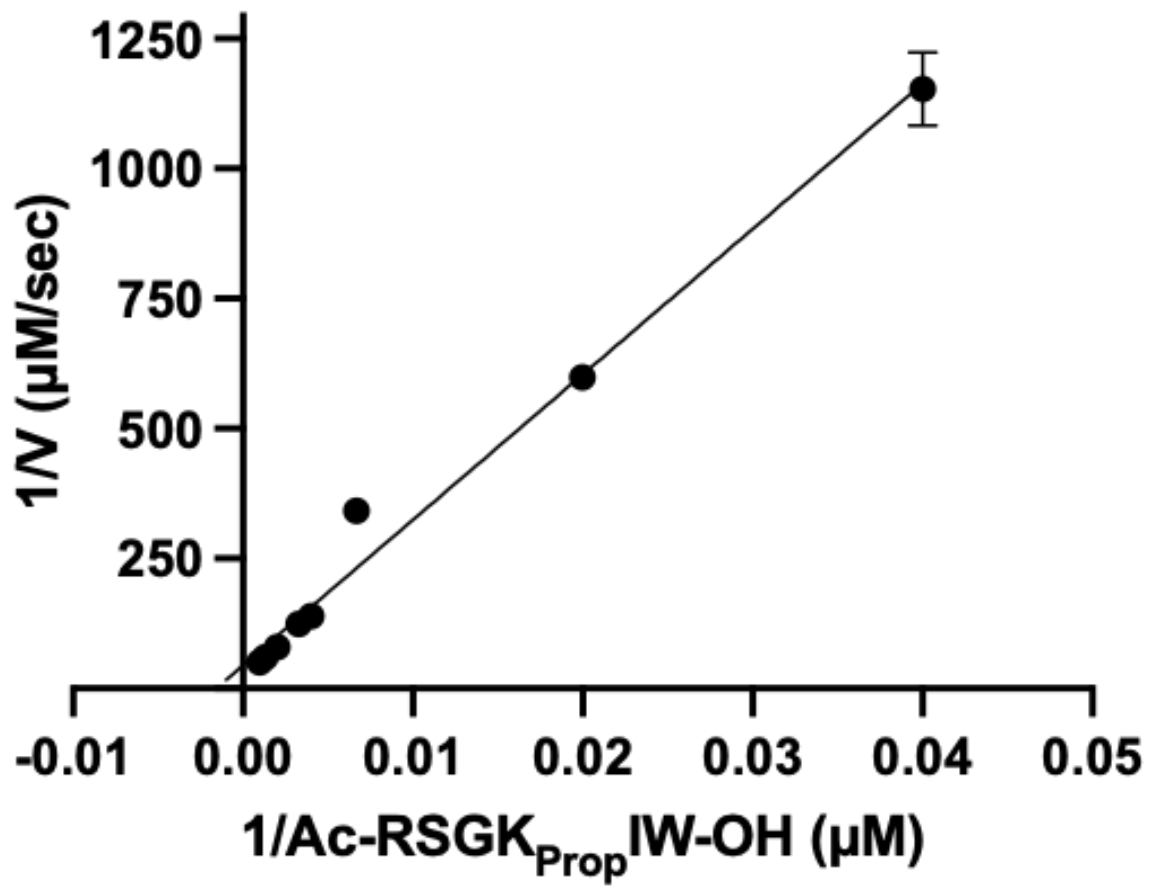

Figure S4. Lineweaver Burke analysis of Ac-RSGK$_{Prop}$IW-OH peptide with *Mt*-Sirt ($Y_{53}F$ mutant) with a fixed concentration of 1 mM NAD^+. Data was fit to y = mx + b, where $k_{cat} = 0.022 \pm 0.008$ sec^{-1}, $K_m = 630$ µM and $k_{cat}/K_m = 3.5 \times 10^1$.

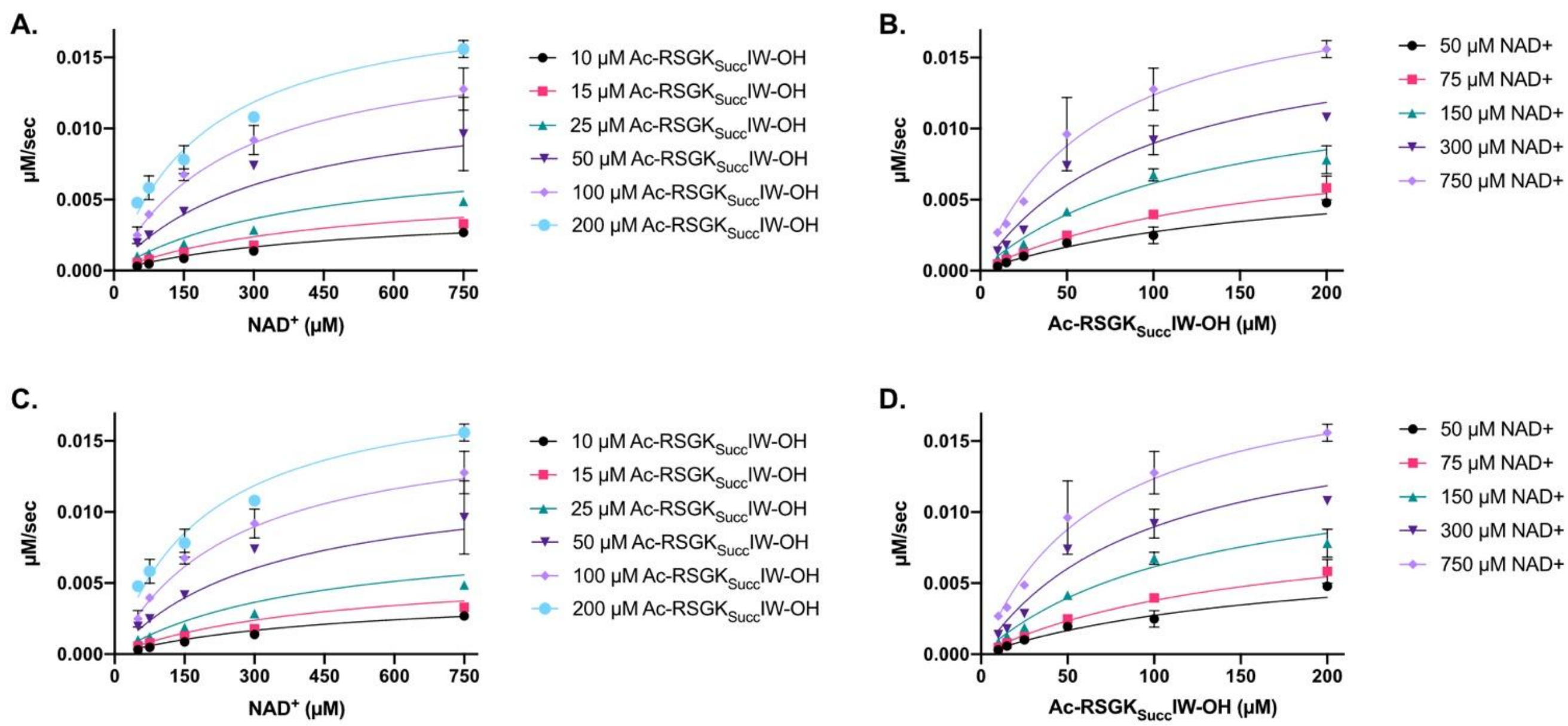

Figure S5. Initial velocity data for *Mt*-Sirt . **A**. NAD^+ (50-800 µM) versus Ac-RSGK$_{Succ}$IW-OH (10-200 µM) fitted to eq. 2. **B**. Ac-RSGK$_{Succ}$IW-OH (10-200 µM) versus NAD^+ (50-750 µM) fitted to eq. 2. **C**. NAD^+ (50-800 µM) versus Ac-RSGK$_{Succ}$IW-OH (10-200 µM) fitted to eq. 3. **B**. Ac-RSGK$_{Succ}$IW-OH (10-200 µM) versus NAD^+ (50-750 µM) fitted to eq. 3.

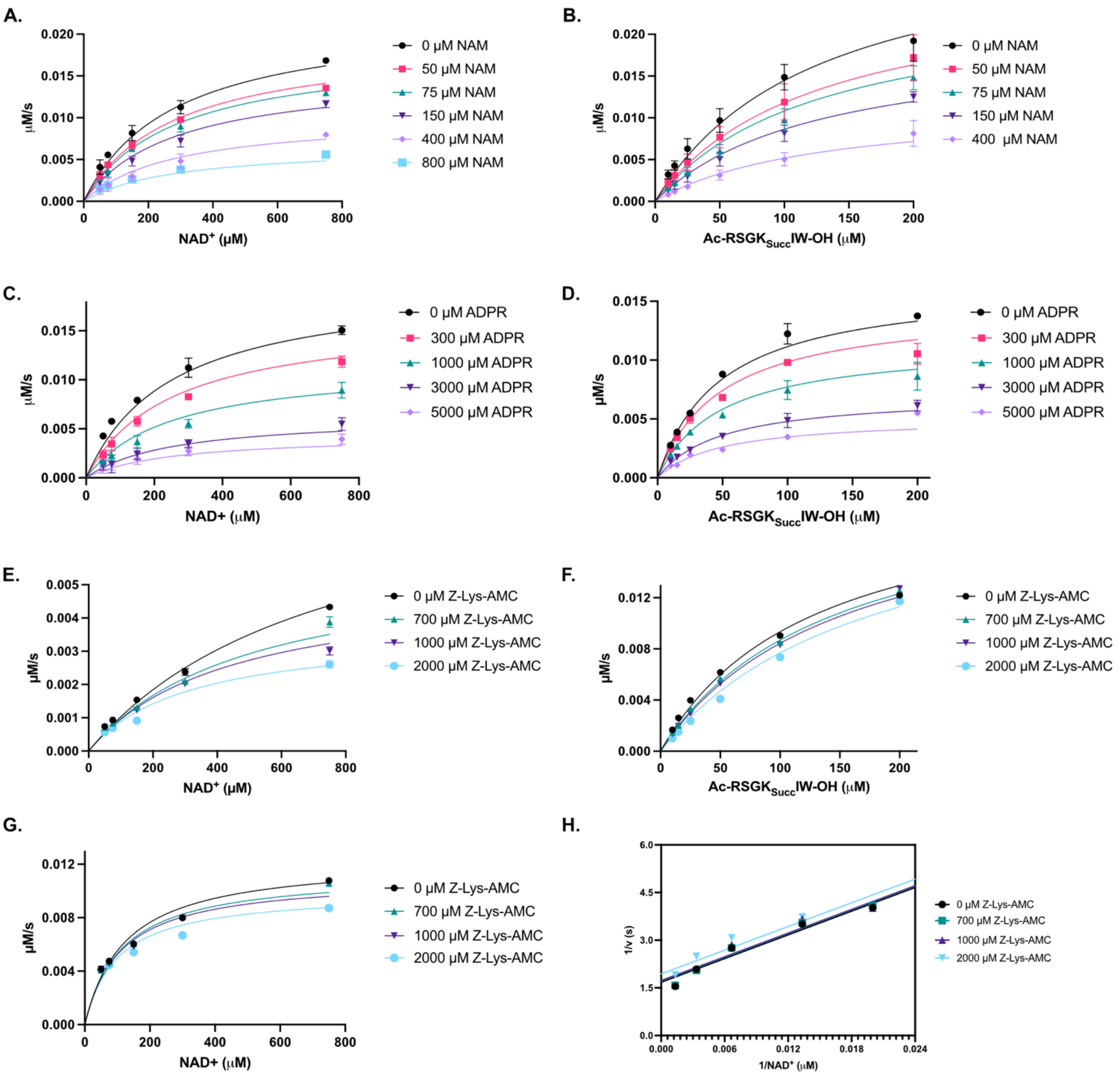

Figure S6. Product inhibition of *Mt*-Sirt by NAM. **A.** NAM (0-800 μM) vs. NAD$^+$ (50-750 μM) with fixed Ac-RSGK$_{Succ}$IW-OH (200 μM) fitted to eq. 5. **B**. NAM (0-400 μM) vs. Ac-RSGK$_{Succ}$IW-OH (10-200 μM) with fixed NAD$^+$ (1 mM) fitted to eq. 5. **C**. ADPR (0-5000 μM) vs. NAD$^+$ (50-750 μM) at fixed Ac-RSGK$_{Succ}$IW-OH (200 μM) fitted to eq. 5 **D**. ADPR (0-5000 μM) vs. Ac-RSGK$_{Succ}$IW-OH (10-200 μM) at fixed NAD$^+$ (1 mM) fitted to eq. 5. **E.** Z-Lys-AMC (0-2mM) vs. NAD$^+$ (50-750 μM) at fixed Ac-RSGK$_{Succ}$IW-OH (40 μM) fitted to eq. 6. **F**. Z-Lys-AMC (0-1.5 mM) vs. Ac-RSGK$_{Succ}$IW-OH (10-200 μM) with fixed NAD$^+$ (1000 μM) fitted to eq 4. **G**. Z-Lys-AMC (0-2mM) vs. NAD$^+$ (50-750 μM) at fixed Ac-RSGK$_{Succ}$IW-OH (200 μM) fitted to eq. 6. **H**. Double reciprocal plotting of data in **G**. fit to the linear form of eq. 6.

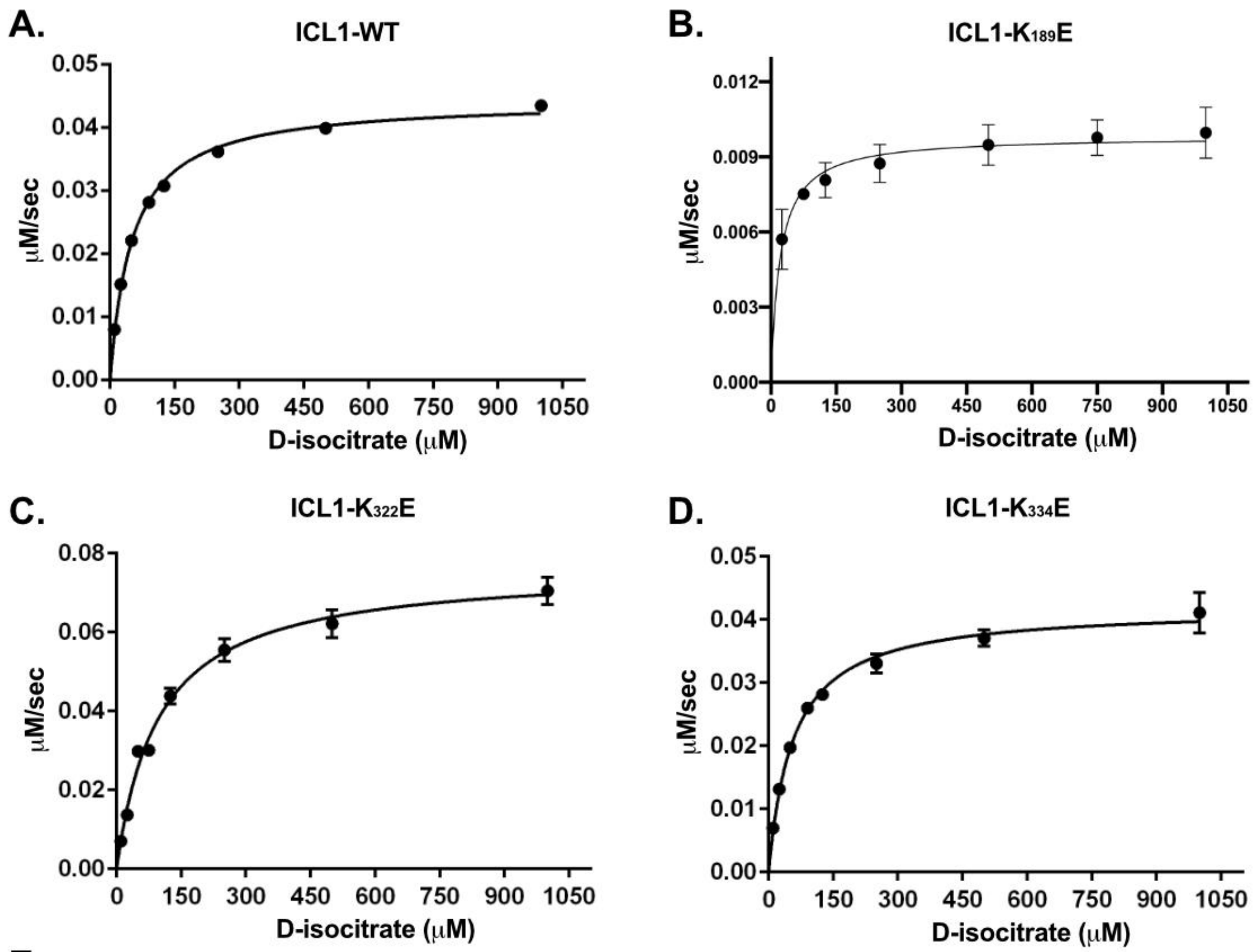

E.

Enzyme	Equation	K_m (μM)	k_{cat} (s^{-1})	k_{cat}/K_m (M^{-1} s^{-1})
ICL1 WT	eq. S1	51 ± 2	22 ± 0.2	$4.3 \pm 0.5 \times 10^5$
ICL1-K189E	eq. S1	22 ± 4	$(20 \pm 6) \times 10^{-4}$	$9.5 \pm 0.3 \times 10^1$
ICL1-K322E	eq. S1	97 ± 8	38 ± 1	$3.9 \pm 0.4 \times 10^5$
ICL1-K334E	eq. S1	57 ± 8	21 ± 1	$3.7 \pm 0.5 \times 10^5$

Figure S7. Michaelis-Menten kinetics of *Mt*ICL1 wild-type and succinylation mimic mutants. **A-D** Varied concentration of D-isocitrate were incubated with fixed ICL1 concentration and linear rates were plotter versus substrate concentration and fitted to eq. S1. **E.** Kinetic parameters determined by fitting of the listed plot to the listed equation.

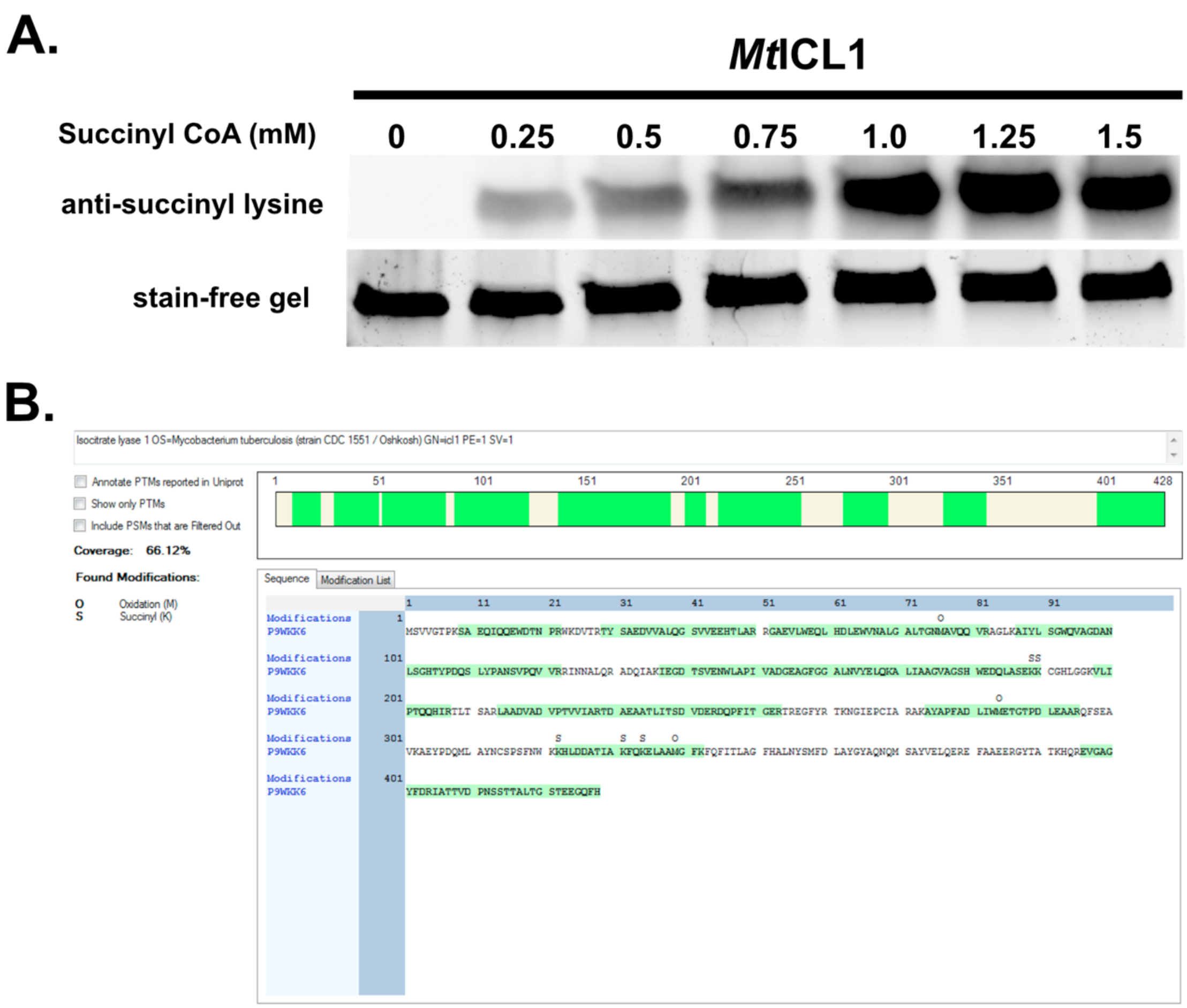

Figure S8. Chemical labeling of *Mt*ICL1 with succinyl CoA and peptide mapping of succinylated *Mt*ICL1. **A**. Western blotting using an anti-succinyl lysine polyclonal antibody to detect lysine succinylation of *Mt*ICL1 that had been treated with varied concentrations of succinyl CoA. **B**. Coverage map of chemically succinylated *Mt*ICL1 (treated with 500 µM succinyl CoA) which underwent tryptic digestion and mass spectrometry analysis. Lysine residues which are labeled with an "S" demarcate a succinylated lysine site as identified by MS/MS analysis.

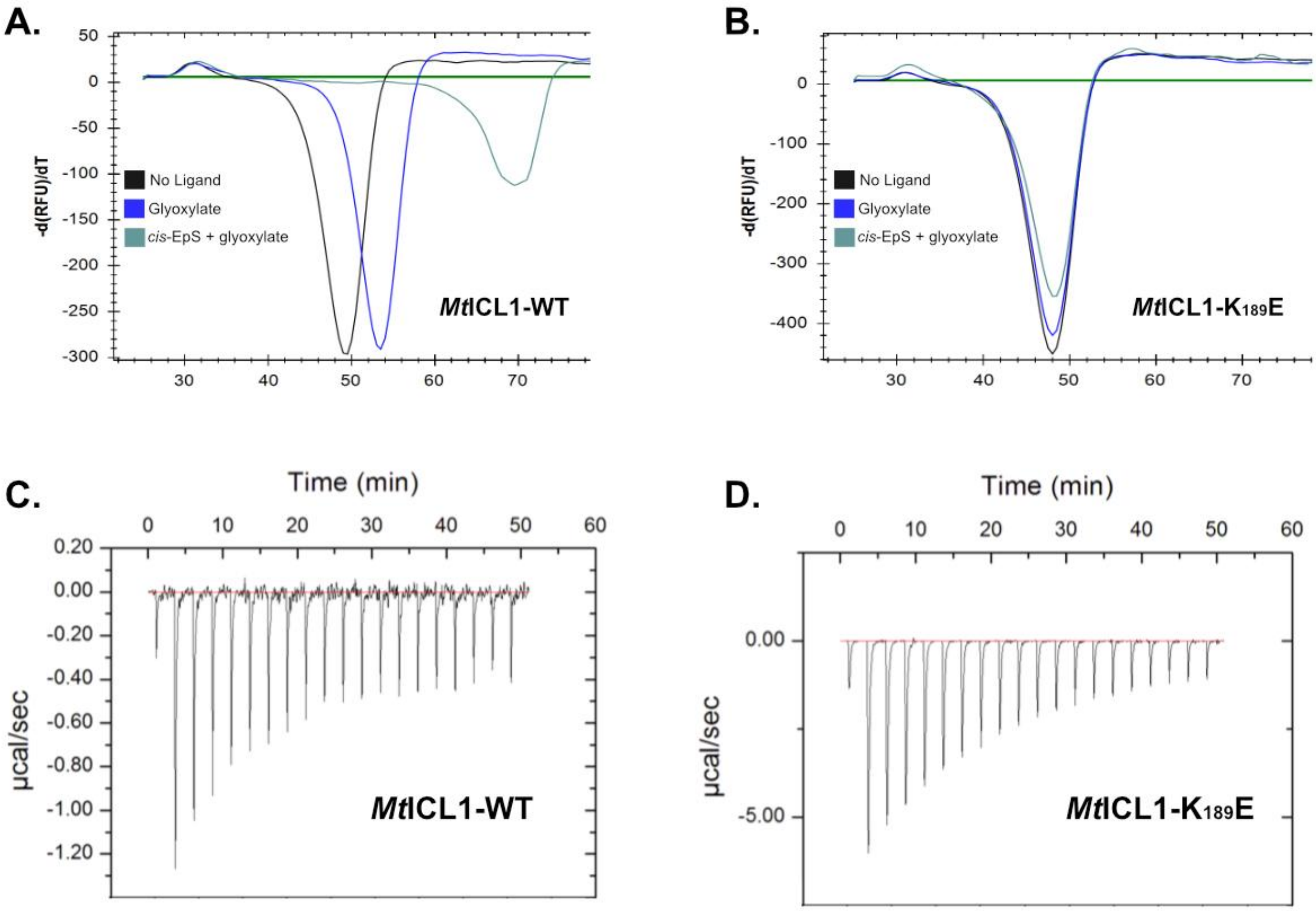

Figure S9. Thermal shift and ITC for *Mt*ICL1 wild-type and K189E succinylation mimetic. **A**. Thermal shift with wild-type *Mt*ICL1 (10 µM) in the presence of no ligand, 1 mM glyoxylate, or 1 mM glyoxylate and 100 µM *cis*-epoxysuccinate (*cis*-EpS). **B**. Thermal shift with *Mt*ICL1-K189E (10 µM) in the presence of no ligand, 1 mM glyoxylate, or 1 mM glyoxylate and 100 µM *cis*-epoxysuccinate (*cis*-EpS). **C**. ITC isotherms demonstrating the binding of glyoxylate to wild-type *Mt*ICL1 (50 µM). **D**. ITC isotherms demonstrating the binding of glyoxylate to *Mt*ICL1-K189E (50 µM).

Cbz-N^{e}-acyl-lysine-AMC HPLC kinetic analysis

To a 1.5 mL Eppendorf tube, 50 mM varied Z-Lys-AMC substrate (dissolved in 100% DMSO), were diluted into DMSO at a 10x concentration and added to 50 mM Tris-HCl (pH 8.0), 1 mM NAD^{+} and 1 mM DTT. [1] To this reaction mixture, *Mt*-Sirt in 50 mM Tris-HCl (pH 8.0) with 1 mM DTT was added at a final concentration of 1 µM to a final volume of 60 uL. The reaction was incubated at 37°C for 30 minutes followed by quenching with 10% TFA (v/v) in water (60 µL). Reaction mixtures were centrifuged for 10 minutes at 15,000 rpm to remove precipitated protein and the supernatant (110 µL) was loaded into to a 96-well plate and analyzed by HPLC (40-mL injection) on a Kinetex PS C18 reverse phase column (Kinetex™ 2.6 µm PS C18 100 Å, LC column 150 x 3 mm, Phenomenex) with elution using a 10-minute gradient: 95% H_2O/5% acetonitrile (ACN) increasing to 0% H_2O/100% ACN. All solvents contained 0.1% (v/v) formic acid. Product formation was measured by the increase of the product peak measured as absorbance at 280 nm in comparison to a no enzyme control.

Cbz-N^{e}-acyl-lysine-AMC kinetic analysis using discontinuous fluorescent assay

To a PCR strip (VWR) varied concentrations of selected Z-Lys(R)-AMC (0-400 µM), dissolved in 100% DMSO, were diluted into DMSO at a 10x concentration and added to 50 mM Tris-HCl, 137 mM NaCl, 2.7 mM KCl, 1 mM $MgCl_2$ (pH 8.5) containing 400 µM NAD^{+} as previously reported.[1] To this reaction mixture, *Mt*-Sirt in 50 mM Tris-HCl, 137 mM NaCl, 2.7 mM KCl, 1 mM $MgCl_2$ (pH 8.5) buffer was added at a final concentration of 400 nM to a final volume of 180 µL. The reaction was allowed to proceed for varied time points in duplicate (2, 5, 10, 15, 20, 25 mins) at which time 25 uL of the reaction mixture was removed and quenched in 225 uL of 50 mM Tris-HCl, 137 mM NaCl, 2.7 mM KCl, 1 mM $MgCl_2$, 10% v/v DMSO (pH 8.5) containing 400 units of trypsin (Sigma) and 2 mM nicotinamide in a black, clear bottom 96 well plate (Greiner). The plate was covered and incubated at 37ºC for 45 minutes followed by reading the fluorescence at 360/460 ex/em on a Biotek® Synergy M2 plate reader equipped with temperature control. The RFU values obtained were converted to µM using a standard AMC curve that was generated under the identical buffer conditions listed above and then multiplied by 10 to account for the dilution of the reaction. The µM concentration obtained was then plotted versus time and fit to y= mx +b to determine the rate of the reaction (µM/min) at a given concentration of substrate followed by plotting the rate versus substrate concentration and fitting with Eq. 1 or y = mx + b if saturation was not observed under the given assay conditions.

Kinetic analysis of MtICL1

Recombinant *Mtb* ICL1 proteins was prepared as previously described[2, 3] and mutants ($K_{189}E$, $K_{322}E$, $K_{334}E$) were prepared used the overlapping primer design approach using Pfu Turbo DNA polymerase (Agilent Technologies). The enzymes were assayed in 96-well clear plates (Greiner) consisting of 250-µL reaction mixtures containing 50 mM HEPES (pH 7.5), 10 mM $MgCl_2$, and 1 mM DTT at 37ºC. In the direction of isocitrate cleavage, product glyoxylate was reacted with 10 mM phenylhydrazine to form its phenylhydrazone product, which was measured spectrophotometrically at 324nm (ε324 = 17,000 $M^{-1}cm^{-1}$). For continuous analysis of *Mt*ICL1 activity, 4 nM of the enzyme (wild type, $K_{322}E$, and $K_{334}E$) or 5 µM ($K_{189}E$) in 50 mM HEPES (pH 7.5), 5 mg/mL BSA, 10 mM $MgCl_2$, 1 mM DTT, pH 7.5 was added to a reaction mixture containing variable concentrations of D-isocitrate (0-1 mM). The rate of the initial velocity for each substrate concentration (µM s^{-1}) was determined and then plotted versus substrate

concentration and fitted to Michaelis-Menten equation Eq. 1 to determine the steady state parameters of V_{max} and K_m for each enzyme.

Chemical Succinylation of Mtb ICL1

Recombinant *Mtb* ICL1 protein was prepared as previously described[2, 3] and incubated at 1 mg/mL in 50 mM HEPES, 150 mM NaCl, 5 mM $MgCl_2$, pH 7.5 with varied concentrations of succinyl CoA (0-1.5 mM) for six hours at 37ºC at a total volume of 50 µL. Following incubation, the samples were frozen to quench the reaction and then 5 µM of the labeled protein was then separated by SDS-PAGE (Biorad 4-15% Mini-PROTEAN® TGX Stain-Free™ protein gel) and semi-dry transferred (Bio-Rad) onto an activated PDVF membrane (GE Healthcare Life Sciences) followed by blocking with TBST/5% wt/v BSA (1 hr, RT). Anti-succinyllysine rabbit polyclonal antibody (PTM Biolabs) was added at a 1:6,000 dilution and incubated overnight at 4°C. The membrane was washed 5x with TBST, probed with goat-anti rabbit HRP conjugate antibody (1:4,000) (Bio-Rad) in TBST/5% wt/v BSA (1 h, RT), washed (5x, TBST) followed by addition of ECL substrate (Bio-Rad) and imaged (ChemiDoc, Bio-Rad).

Intrinsic protein fluorescence of MtICL1

*Mt*ICL1 or *Mt*ICL1 K_{189}E (1 µM of monomer) in 50 mM HEPES, 10 mM $MgCl_2$, 1 mM DTT (pH 7.5) was combined with glyoxylate (1 mM), D-isocitrate (1 mM), and *cis*-epoxysuccinate (100 µM) with glyoxylate (1 mM). Reaction mixtures (200 µL) were excited at λ_{ex} = 295 nm, and the intrinsic fluorescence emission spectra (λ_{em} = 315–380 nm) was determined following a 10-minute incubation at 37ºC using a Biotek Synergy plate reader with black, clear-bottom, 96-well plates (Greiner). The λ_{max} of ICL1 fluorescence was determined to be 325 nm. All data were corrected by subtracting the background fluorescence observed in a sample of buffer. Upon addition of ligand the spectrum was recorded multiple time points. Each spectrum was normalized, assigning the fluorescent intensity of protein alone at 325 nm to 1.

Differential scanning fluorimetry

To a 96 well PCR plate, 0.5 mg/mL (10 µM monomer) *Mt*ICL1 and *Mt*ICL1 K_{189}E in 50 mM HEPES, 10 mM $MgCl_2$ (pH 7.5) in the presence of 5x Sypro dye from a 5000x stock (Invitrogen) and varied ligands (glyoxylate (1 mM), D-isocitrate (1 mM), *cis*-epoxysuccinate (100 µM) with glyoxylate (1 mM). The samples were placed in a real-time PCR (Bio-Rad) and melting was assessed from 20ºC to 95ºC in increments of 0.5ºC every 30 seconds. The thermal melts were then processed using the Bio-Rad CFX Maestro software and changes in melting transition temperature versus the non-liganded protein was assessed.

Isothermal calorimetry with MtICL1

ITC binding assays conducted at 25°C using a MicroCal iTC200 microcalorimeter (GE) with power setting of 11 µcal sec^{-1} and stirring of 750 rpm. The reference cell (200 µL) was loaded with diH_2O water, and the sample cell (280 µL) contained 50 µM *Mt*ICL1 or *Mt*ICL1 K_{189}E in 50 mM HEPES, 10 mM $MgCl_2$, 1 mM DTT, and 5% (v/v) glycerol (pH 7.5). The injection syringe was filled with glyoxylate (11 mM) in the same buffer as listed above. Ligand into buffer titrations were carried out to allow for background subtraction of the heat of dilution and also mixing. Titration of glyoxylate with the *Mt*ICL1 proteins of interest were carried out and the data generated was not analyzed for binding thermodynamics or affinity due to the high reported ratio

of hydrate to aldehyde for glyxoyate in aqueous medium (163:1)[4] and evidence that *Mt*ICL1 can copurify with glyoxylate,[2] both of which complicate accurate analysis.

Bio-orthogonal Labelling of MtSirt in mc²7000 Cells

To a 10-mL culture of mc²7000 in 7H9/OADC medium grown to OD_{600} = 0.8-1.0, a 50 mM solution of tetradec-13-ynoic acid (Alk-12, Click Chemistry Tools) in DMSO was added to the cells to reach a final concentration of 50 µM (10 µL). A control culture with DMSO alone was included. These cells were grown for an additional 24 hours at 37ºC after which time, cells were centrifuged (4k rpm), washed 6x with ice cold PBS buffer, and stored at -20°C. The stored pellets were resuspended (500 µL) in a mixture containing 200 mM Tris-HCl (pH 8.0), 4% wt/v CHAPS, 1 M NaCl, 8 M urea, 5 mM nicotinamide, and 50 uL cOmplete EDTA-free Protease Cocktail Inhibitor (1 tablet dissolved in 2 mL MilliQ H_2O, 25x, Roche) and 50 µL lysozyme (0.1 mg/mL). Cells were disrupted using a bead-beater homogenizer containing silica-zirconium beads for 60 sec, followed by immersion of 3 min on ice (8x). The lysate was centrifuged at 13,000 rpm at 4 °C for 15 min, and the supernatant was removed. Protein concentration was determined using the BCA assay. Labeling of the Alk-12 treated cell lysates occurred in a stepwise fashion, where TBTA in 1:4 DMSO: n-butanol (v/v) (100 µM final) was added to $CuSO_4$ in water (1 mM final), followed by the addition of Cy7-azide (Click Chemistry Tools) in DMSO (20 µM final), 24 µg cell lysate, and finally sodium ascorbate in water (2.5 mM final). This reaction was incubated at room temperature for 1.5 hours, and quenched by the addition ice-cold methanol (200 µL), chloroform (75 µL), and water (150 µL), followed by vortexing and centrifugation at 15,000 x g at 4 ºC for 15 minutes. The top layer of liquid was carefully removed and discarded, and the protein precipitate formed between two liquid phases was not disturbed. An additional 1 mL of ice-cold methanol was added into the tube, and the sample was vortexed and then centrifuged at 15,000 x g at 4 ºC for 20 minutes. The supernatant was discarded, and this was washing step was repeated an additional time. Following removal of the supernatant the protein pellet was allowed to air-dry at room temperature. To the dried pellet 4% SDS buffer (4% SDS (w/v), 50 mM TEA, 150 mM NaCl, pH 7.4) was added, and the pellet was sonicated to achieve a clarified solution. Then, the solution was either added to 4x loading dye (240 mM Tris-HCl, 8% SDS (w/v), 0.004 % bromophenol blue (to avoid fluorescence from the dye), DTT (5 mM final), and MilliQ water to a final volume of 24 µL or to 4x loading dye (240 mM Tris-HCl, 8% SDS (w/v), 0.004 % bromophenol blue (to avoid fluorescence from the dye), DTT (5 mM final), 60 mM hydroxylamine (pH 7.2), and MilliQ water to a final volume of 24 µL. The samples were heated to 95 ºC for 10 minutes, followed by a brief centrifugation at 15,000 x g for 1 min. Protein standards (Bio-Rad, Precision Plus Protein™ Dual Color Standards) were diluted 10,000-fold in 4% SDS buffer and 4x loading dye to avoid strong fluorescence from the standards during imaging. Samples were analyzed on a 4-12% Bis-Tris Nu-PAGE gel (Invitrogen). To each well, 20 µg of labeled lysate was loaded, and the gel was run at 60 V for 30 minutes followed by 120 V for ~90 minutes. The gels were destained (10% acetic acid, 40% methanol, 50% water) for 1 hour to overnight in the dark, followed by imaging the gel on a ChemiDoc imager (Bio-Rad) using the Cy7 blot setting. To visualize the two fluorescent proteins of the protein standards ladder, we used the rhodamine setting. The gels were also analyzed by Coomassie Brilliant Blue R-250 staining (Bio-Rad) to ensure equal loading.

Synthesis:

Method A. Amide formation with 7-amino-4-methyl coumarin
Cbz-Lys and 7-amino-4-methyl coumarin (1mmol/1mmol lysine) was purged with $N_{2(g)}$ yielding positive pressure. Anhydrous tetrahydrafuran (12 mL/mmol lysine) was was added followed by the addition of N,N-diisopropylethylamine (3 mmol/1 mmol lysine). Propylphosphonic anhydride in a 50% wt. solution of ethyl acetate (2 mmol/1 mmol lysine) was added dropwise followed by allowing the reaction to proceed overnight. Solvents were removed under reduced pressure and the yellow oil was solubilized in DCM and washed with water (4x) and brine (2x). The organic layer was dried over Na_2SO_4 and removed under reduced pressure.

***N*^6-Acetyl-*N*^2-((benzyloxy)carbonyl)-*L*-lysine Cbz-Lys(ac)-OH).**[5] A flask charged with Cbz-Lys-OH (300 mg, 1.07 mmol) was purged with $N_{2(g)}$. Then anhydrous pyridine (10 mL) was added followed by the dropwise addition of acetic anhydride (101 uL, 1.07 mmol). The reaction was allowed to proceed overnight followed by acidification to pH 1 with 2 N HCl and extraction with ethyl acetate (3x 20 mL). The pooled organic layers were then washed with 5% $NaCO_3$ and this bicarbonate layer was acidified to pH 1 and extracted with ethyl acetate (3x, 20 mL). To combined organic layers were washed with brine (2x, 20 mL), dried over Na_2SO_4, and concentrated under reduced pressure. The product was moved directly to the next step with no purification.

Benzyl (*S*)-(6-acetamido-1-((4-methyl-2-oxo-2*H*-chromen-7-yl)amino)-1-oxohexan-2-yl)carbamate (Cbz-Lys(ac)-AMC). Method A. Z-Lys(ac)-OH (256 mg, 0.8 mmol) and 7-amino-4-methylcoumarin (140 mg, 0.8 mmol), N,N-diisopropylethylamine (420 µL, 2.44 mmol), T3P (950 mL, 1.6 mmol). Semi-preparative HPLC of the molecule resulted in 90 mg (23.5% yield). LC-MS t_R: 4.14 min, m/z 480.07 [M+1H], $C_{16}H_{22}N_2O_5$, Calcd. 480.21 [M+1H] ^{1}H NMR (400 MHz, DMSO) δ 1.26 – 1.50 (m, 4H), 1.56 – 1.76 (m, 2H), 1.77 (s, 3H), 2.41 (d, *J* = 1.2 Hz, 3H), 3.01 (q, *J* = 6.3 Hz, 2H), 4.16 (dd, *J* = 10.8, 5.6 Hz, 1H), 5.05 (s, 2H), 6.27 (d, *J* = 1.4 Hz, 1H), 7.28 – 7.43 (m, 5H), 7.51 (dd, *J* = 8.6, 2.1 Hz, 1H), 7.62 (d, *J* = 7.6 Hz, 1H), 7.69 – 7.83 (m, 3H), 10.45 (s, 1H).

Benzyl (*S*)-(1-((4-methyl-2-oxo-2*H*-chromen-7-yl)amino)-1-oxo-6-propionamidohexan-2-yl)carbamate (Cbz-Lys(prop)-AMC). Z-Lys-OH (150 mg, 0.535 mmol) was dissolved in 1N NaOH (2 mL) / THF (2mL), covered with a rubber septum, purged with $N_{2(g)}$ and cooled to 0ºC on an ice bath. Propionyl chloride (50 uL, 0.56 mmol) was added dropwise and the reaction was allowed to warm to RT and was stirred overnight. The reaction was then cooled in an ice bath and washed with ice-cold Et_2O (10 mL). The aqueous phase was acidified to pH ~4 with 1N HCl and extracted with ethyl acetate (20 mL, 3x). The combined organic phases were dried over Na_2SO_4, and concentrated under reduced pressure to yield the crude product which was progressed to the next step without purification. Subsequent coupling using Method A. Cbz-Lys(prop)-OH (78 mg, 0.24 mmol), 7-amino-4-methyl coumarin (42 mg, 0.24 mmol), THF (5 mL), DIEA, (167 µL, 0.96 mmol), T3P (286 µL, 0.48 mmol). Semi-preparative HPLC of the crude molecule resulted in pure title molecule (28.4 mg, 24% yield). LC-MS t_R: 4.37 min, m/z 492.16 [M-1H], $C_{27}H_{31}N_3O_6$, Calcd. 492.22 [M-1H] ^{1}H NMR (400 MHz, MeOD) δ 1.10 (t, *J* = 7.6 Hz, 3H), 1.38 – 1.64 (m, 5H), 1.69 – 1.97 (m, 2H), 2.17 (q, *J* = 7.6 Hz, 2H), 2.47 (d, *J* = 1.3 Hz, 3H), 3.13 – 3.26 (m, 2H), 4.26 (q, *J* = 6.9 Hz, 1H), 5.04 – 5.22 (m, 2H), 6.14 – 6.30 (m, 1H), 7.11 – 7.45 (m, 5H), 7.45 – 7.57 (m, 1H), 7.71 (d, *J* = 8.7 Hz, 1H), 7.82 (d, *J* = 15.9 Hz, 1H).

Benzyl (*S*)-(6-butyramido-1-((4-methyl-2-oxo-2*H*-chromen-7-yl)amino)-1-oxohexan-2-yl)carbamate (Cbz-Lys(but)-AMC). Z-Lys-OH (150 mg, 0.535 mmol) was dissolved in 1N NaOH (2 mL) / THF (2mL), covered with a rubber septum, purged with $N_{2(g)}$ and cooled to 0ºC on an ice bath. Butyryl chloride (58 uL, 0.56 mmol) was added dropwise and the reaction was allowed to warm to RT and was stirred overnight. The reaction was then cooled in an ice bath and washed with ice-cold Et_2O (10 mL). The aqueous phase was acidified to pH ~4 with 1N HCl and extracted with ethyl acetate (20 mL, 3x). The combined organic phases were dried over Na_2SO_4, and concentrated under reduced pressure to yield the crude product which was progressed to the next step without purification. Subsequent coupling using Method A. Cbz-Lys(but)-OH (112 mg, 0.32 mmol), 7-amino-4-methyl coumarin (56 mg, 0.24 mmol), THF (7 mL), DIEA, (223 µL, 1.29 mmol), T3P (381 µL, 0.64 mmol). Semi-preparative HPLC of the crude molecule resulted in pure title molecule (45.6 mg, 28% yield). LC-MS t_R: 4.580 min, m/z 506.19 [M-1H], $C_{28}H_{33}N_3O_6$, Calcd. 506.24 [M-1H]. ^1H NMR (400 MHz, MeOD) δ 0.90 (t, *J* = 7.4 Hz, 3H), 1.34 – 1.70 (m, 7H), 1.69 – 1.99 (m, 2H), 2.12 (dd, *J* = 8.0, 6.8 Hz, 2H), 2.46 (d, *J* = 1.3 Hz, 3H), 3.12 – 3.28 (m, 2H), 4.27 (t, *J* = 7.2 Hz, 1H), 5.12 (d, *J* = 3.6 Hz, 2H), 6.15 – 6.30 (m, 1H), 7.34 (dt, *J* = 18.2, 6.7 Hz, 5H), 7.49 (dd, *J* = 8.9, 2.0 Hz, 1H), 7.67 (d, *J* = 8.7 Hz, 1H), 7.74 – 7.86 (m, 1H).

Benzyl *tert*-butyl (6-((4-methyl-2-oxo-2*H*-chromen-7-yl)amino)-6-oxohexane-1,5-diyl)(*S*)-dicarbamate (Cbz-Lys(boc)-AMC). Method A. Cbz-Lys(boc)-OH (400 mg, 1.05 mmol), 7-amino-4-methyl coumarin (184 mg, 1.05 mmol), THF (12 mL), DIEA (550 µL, 3.15 mmol), T3P (1.25 mL, 2.1 mmol). Semi-preparative HPLC yielded (344 mg, 61% yield). LC-MS t_R: 5.27 min, m/z 438.1 [M+1H, -Boc], $C_{29}H_{35}N_3O_7$, Calcd. 538.25 [M+1H], 438.2[M+1, -Boc]

(*S*)-3-((5-(((Benzyloxy)carbonyl)amino)-6-((4-methyl-2-oxo-2*H*-chromen-7-yl)amino)-6-oxohexyl)amino)-3-oxopropanoic acid (Cbz-Lys(mal)-AMC). A round bottom flask charged with Z-Lys(boc)-AMC (150 mg, 0.28 mmol) was purged with $N_{2(g)}$ yielding positive pressure. Anhydrous DCM (4 mL) was added followed by 33% v/v TFA (2 mL) and the reaction was allowed to proceed for 1.5 h at which time the solvents were removed under reduced pressure to yield Cbz-Lys-AMC • TFA. The residue was co-evaporated with Et_2O (2x, 10 mL) and then moved to the next step. To the flask, mono-*t*-butyl malonate (71 mg, 0.46 mmol) was added followed by purging with $N_{2(g)}$ to yield positive pressure. Anhydrous DCM (5 ml) was added followed by the addition of N,N-diisopropylethylamine (265 µL, 1.52 mmol). Propylphosphonic anhydride in a 50% wt. solution of ethyl acetate (452 mL, 0.76 mmol) was added dropwise and allowed to proceed overnight at RT. The reaction mixture was diluted in DCM (~30 mL) and washed with water (15 mL, 4x) and brine (15 mL, 2x). The organic layer was dried over Na_2SO_4, and solvents were removed under reduced pressure. The crude product was then solubilized in DCM (5 mL) and 40% TFA v/v (2 mL) and the solvents were removed upon completion as determined by LC-MS. The crude residue was dissolved in a minimal amount of DMF and subjected to semi-preparative HPLC purification to yield the pure title compound (90.6 mg, 62% yield). LC-MS t_R: 4.08 min, m/z 522.16 [M-1H], $C_{27}H_{29}N_3O_8$, Calcd. 524.2 [M-1H] ^1H NMR (400 MHz, MeOD) δ 1.34 – 1.67 (m, 4H), 1.66 – 1.96 (m, 2H), 2.45 (d, *J* = 1.2 Hz, 3H), 3.17 – 3.26 (m, 4H), 4.25 (s, 1H), 5.11 (d, *J* = 3.0 Hz, 2H), 6.07 – 6.30 (m, 1H), 7.10 – 7.42 (m, 5H), 7.47 (dd, *J* = 8.5, 2.1 Hz, 1H), 7.68 (d, *J* = 8.7 Hz, 1H), 7.73 – 7.82 (m, 1H).

(S)-4-((5-(((Benzyloxy)carbonyl)amino)-6-((4-methyl-2-oxo-2H-chromen-7-yl)amino)-6-oxohexyl)amino)-4-oxobutanoic acid (Cbz-Lys(succ)-AMC). A round bottom flask charged with Z-Lys(boc)-AMC (150 mg, 0.28 mmol) was purged with $N_{2(g)}$ yielding positive pressure. Anhydrous DCM (4 mL) was added followed by 33% v/v TFA (2 mL) and the reaction was allowed to proceed for 1.5 h at which time the solvents were removed under reduced pressure. The residue was co-evaporated with Et_2O (2x, 10 mL) and then moved to the next step. To the flask succinic anhydride (28 mg, 0.28 mmol) was added followed by purging the flask with $N_{2(g)}$ to yield positive pressure. Anhydrous DMF (3 mL) and then DIEA (300 uL, 1.7 mmol) was added. The reaction was allowed to proceed overnight at RT at which time the reaction mixture was diluted with H_2O (20 mL), acidified to pH 2, and extracted with ethyl acetate (3x, 20 mL). The pooled organic layer was washed with brine (2x, 15 mL) dried over Na_2SO_4, and concentrated under reduced pressure. This crude product was dissolved in a minimal amount of DMF and subjected to semi-preparative HPLC purification to yield the pure title compound (94 mg, 62% yield). LC-MS t_R: 4.03 min, m/z 536.2 [M+1H], $C_{28}H_{31}N_3O_8$, Calcd. 536.21 [M+1H] ^{1}H NMR (400 MHz, DMSO) δ 1.17 – 1.53 (m, 4H), 1.52 – 1.83 (m, 2H), 2.28 (t, *J* = 6.7 Hz, 2H), 2.35 – 2.47 (m, 5H), 3.02 (q, *J* = 6.3 Hz, 2H), 3.39 (q, *J* = 7.0 Hz, 2H), 4.15 (td, *J* = 8.3, 5.1 Hz, 1H), 5.05 (s, 2H), 6.27 (d, *J* = 1.4 Hz, 1H), 7.08 – 7.46 (m, 4H), 7.51 (dd, *J* = 8.6, 2.1 Hz, 1H), 7.62 (d, *J* = 7.6 Hz, 1H), 7.68 – 7.88 (m, 3H), 10.46 (s, 1H).

5-(*tert*-Butoxy)-5-oxopentanoic acid. A round bottom flask charged with glutaric anhydride (2 g, 17.5 mmol) and $ZnCl_2$ (360 mg, 2.6 mmol) was purged with $N_{2(g)}$ yielding positive pressure. Tert-butyl alcohol (10 ml) was added and the reaction was heated to 60ºC and monitored by TLC (p-anisaldehyde stain) and was stopped after 18 hours. The reaction mixture was allowed to cool followed by the addition of sat. $NaCO_3$ (20 mL) followed by extraction with DCM (50 mL, 3x). The aqueous phase was then acidified with conc. H_2SO_4 to pH 1 and extracted with DCM (50 mL, 3x). The acidified organic extract was then washed with brine (2x, 40 mL), dried over Na_2SO_4 and concentrated to yield the title compound as a clear oil in high purity (1.04 g, 31.5% yield). ^{1}H NMR (400 MHz, $CDCl_3$) δ 1.29 (s, 1H), 1.46 (s, 9H), 1.94 (q, *J* = 7.3 Hz, 2H), 2.32 (t, *J* = 7.3 Hz, 2H), 2.43 (t, *J* = 7.4 Hz, 2H).

(*S*)-5-((5-(((Benzyloxy)carbonyl)amino)-6-((4-methyl-2-oxo-2*H*-chromen-7-yl)amino)-6-oxohexyl)amino)-5-oxopentanoic acid (Cbz-Lys(glut)-AMC). A round bottom flask charged with Z-Lys(boc)-AMC (150 mg, 0.28 mmol) was purged with $N_{2(g)}$ yielding positive pressure. Anhydrous DCM (4 mL) was added followed by 33% v/v TFA (2 mL) and the reaction was allowed to proceed for 1.5 h at which time the solvents were removed under reduced pressure to yield Cbz-Lys-AMC • TFA. The residue was co-evaporated with Et_2O (2x, 10 mL) and then moved to the next step. To the flask **5-(*tert*-butoxy)-5-oxopentanoic acid** (105 mg, 0.56 mmol) was added followed by purging the flask with $N_{2(g)}$ to yield positive pressure. Anhydrous DCM (5 ml) was added followed by the addition of N,N-diisopropylethylamine (243 µL, 1.4 mmol). Propylphosphonic anhydride in a 50% wt. solution of ethyl acetate (333 mL, 0.56 mmol) was added dropwise and allowed to proceed overnight at RT. The reaction mixture was diluted in DCM (~30 mL) and washed with water (15 mL, 4x) and brine (15 mL, 2x). The organic layer was dried over Na_2SO_4, and solvents were removed under reduced pressure. The crude product was then solubilized in DCM (5 mL) and 40% TFA v/v (2 mL) and the solvents were removed upon completion as determined by LC-MS. The crude residue was dissolved in a minimal amount of DMF and subjected to semi-preparative HPLC purification to yield the pure title

compound (80.0 mg, 52% yield). LC-MS t_R: 4.06 min, m/z 550.19 [M-1H], $C_{29}H_{33}N_3O_8$, Calcd. 550.23 [M-1H] ^{1}H NMR (400 MHz, MeOD) δ 1.34 – 1.64 (m, 4H), 1.68 – 1.94 (m, 4H), 2.21 (dd, J = 8.1, 6.8 Hz, 2H), 2.30 (t, J = 7.3 Hz, 2H), 2.40 – 2.52 (m, 3H), 3.19 (t, J = 6.6 Hz, 2H), 4.27 (dd, J = 8.8, 5.4 Hz, 1H), 5.13 (d, J = 3.3 Hz, 2H), 6.23 (s, 1H), 7.34 (ddt, J = 19.5, 13.8, 7.2 Hz, 5H), 7.47 (dd, J = 8.7, 2.1 Hz, 1H), 7.68 (d, J = 8.7 Hz, 1H), 7.72 – 7.86 (m, 1H).

Method B. Synthesis of Fmoc-Lysine(x)-OH amino acids
The appropriate carboxylic acid chain and N-hydroxysuccinimide (0.96 mmol/1 mmol acid) was dissolved in anhydrous DMF (0.75 mL/mmol acid) under a positive pressure of $N_{2(g)}$. The dropwise addition of N,N' diisopropylcarbodiimide (0.96 mmol/1 mmol acid) was added to the reaction and stirred for two hours. The reaction mixture was filtered and the filtrate was directly transferred to a flask containing Fmoc-Lys-OH (0.75 mmol/1 mmol acid) dissolved in DMF (2 mL/1 mmol acid) and N,N-diisopropylethylamine (1.9 mmol/1 mmol acid). Following 30 minutes, the reaction was quenched with water (1 mL/1 mmol acid), acidified with 1 M HCl (1 mL/1 mmol acid), and extracted with ethyl acetate (3x). The organic layers were washed with saturated bicarbonate (3x), brine (2x), and dried over Na_2SO_4. Silica chromatography (0-3% MeOH/DCM, 0.5% formic acid) gave the title compounds as white/off white fluffy solids which upon LC-MS analysis were moved directly to SPPS.

***N*^{2}-(((9*H*-Fluoren-9-yl)methoxy)carbonyl)-*N*^{6}-propionyl-*L*-lysine (Fmoc-Lys(prop)-OH).**
Method B. Propionic acid (1.2 mL, 16.3 mmol), N-hydroxysuccinimide (1.81 g, 15.7 mmol), DMF (12 mL), N,N' diisopropylcarbodiimide (2.44 mL, 15.7 mmol), Fmoc-Lys-OH (4g, 10.85 mmol), DIEA (5.48 mL, 31.5 mmol), and DMF (35 mL). Silica column (0-5% MeOH/DCM, 0.5% formic acid) to yield (2.59 g, 56% yield). LC-MS t_R: 4.49 min, m/z 425.24 [M+1H], $C_{24}H_{28}N_2O_5$, Calcd. 425.2 [M+1H]

***N*^{2}-(((9*H*-Fluoren-9-yl)methoxy)carbonyl)-*N*^{6}-butyryl-*L*-lysine (Fmoc-Lys(but)-OH).**
Method B. Butanoic acid (1.87 mL, 20.4 mmol), N-hydroxysuccinimide (2.27 g, 19.7 mmol), DMF (15 mL), N,N' diisopropylcarbodiimide (3.05 mL, 19.7 mmol), Fmoc-Lys-OH (5g, 13.6 mmol), DIEA (6.86 mL, 39.4 mmol), and DMF (35 mL). Silica column (0-5% MeOH/DCM, 0.5% formic acid) to yield (4.21 g, 71% yield). LC-MS t_R: 4.69 min, m/z 439.23 [M+1H], $C_{25}H_{30}N_2O_5$, Calcd. 439.22 [M+1H]

***(E)*-N^2-(((9H-Fluoren-9-yl)methoxy)carbonyl)-N^6-(but-2-enoyl)-L-lysine (Fmoc-Lys(crot)-OH).** Method B. Crotonic acid (1.75 g, 20.4 mmol), N-hydroxysuccinimide (2.27 g, 19.7 mmol), DMF (15 mL), N,N' diisopropylcarbodiimide (3.05 mL, 19.7 mmol), Fmoc-Lys-OH (5g, 13.6 mmol), DIEA (6.86 mL, 39.4 mmol), and DMF (35 mL). Silica column (0-5% MeOH/DCM, 0.5% formic acid) to yield (3.0 g, 50% yield). LC-MS t_R: 9.83 min, m/z 437.1 [M+1H], $C_{25}H_{28}N_2O_5$, Calcd. 437.20 [M+1H]

***N*^{2}-(((9*H*-Fluoren-9-yl)methoxy)carbonyl)-*N*^{6}-tetradecanoyl-*L*-lysine (Fmoc-Lys(myr)-OH).**
Method B. Myristic acid (2.3 g, 10.2 mmol), N-hydroxysuccinimide (1.3 g, 9.9 mmol), DMF (8 mL), N,N' diisopropylcarbodiimide (1.53 mL, 9.9 mmol), Fmoc-Lys-OH (2.5 g, 6.8 mmol), DIEA (3.43 mL, 19.7 mmol), and DMF (20 mL). Silica column (0-5% MeOH/DCM, 0.5%

formic acid) to yield (1.74 g, 44% yield). LC-MS t_R: 7.47 min, m/z 579.2 [M+1H], $C_{35}H_{50}N_2O_5$, Calcd. 579.37 [M+1H]

***N*[2]-(((9*H*-Fluoren-9-yl)methoxy)carbonyl)-*N*[6]-(3-(*tert*-butoxy)-3-oxopropanoyl)-*L*-lysine (Fmoc-Lys(mal-O-*t*But)-OH).** Method B. Mono-*t*-butyl malonate (3.14 g, 20.4 mmol), N-hydroxysuccinimide (2.27 g, 19.7 mmol), DMF (15 mL), N,N' diisopropylcarbodiimide (3.05 mL, 19.7 mmol), Fmoc-Lys-OH (5g, 13.6 mmol), DIEA (6.86 mL, 39.4 mmol), and DMF (35 mL). Silica column (0-5% MeOH/DCM, 0.5% formic acid) to yield (5.3 g, 77% yield). LC-MS t_R: 5.06 min, m/z 511.19 [M+1H], $C_{28}H_{34}N_2O_7$, Calcd. 511.24 [M+1H], m/z 455.23 [M+1H, - *t*But], $C_{24}H_{26}N_2O_7$, Calcd. 455.17 [M+1H, - *t*But]

***N*[2]-(((9*H*-Fluoren-9-yl)methoxy)carbonyl)-*N*[6]-(4-(*tert*-butoxy)-4-oxobutanoyl)-*L*-lysine (Fmoc-Lys(succ-O-*t*But)-OH).** Method B. Mono-*t*-butyl succinate (3.53 g, 20.4 mmol), N-hydroxysuccinimide (2.27 g, 19.7 mmol), DMF (15 mL), N,N' diisopropylcarbodiimide (3.05 mL, 19.7 mmol), Fmoc-Lys-OH (5g, 13.6 mmol), DIEA (6.86 mL, 39.4 mmol), and DMF (35 mL). Silica column (0-5% MeOH/DCM, 0.5% formic acid) to yield (4.1 g, 57% yield). LC-MS t_R: 5.15 min, m/z 523.32 [M-1H], $C_{29}H_{36}N_2O_7$, Calcd. 524.25 [M-1H]

***N*[2]-(((9*H*-Fluoren-9-yl)methoxy)carbonyl)-*N*[6]-(5-(*tert*-butoxy)-5-oxopentanoyl)-*L*-lysine (Fmoc-Lys(glut-O-*t*But)-OH).** Method B. Mono-*t*-butyl glutaryl (3.06 g, 16.3 mmol), N-hydroxysuccinimide (1.81 g, 15.7 mmol), DMF (12 mL), N,N' diisopropylcarbodiimide (2.44 mL, 15.7 mmol), Fmoc-Lys-OH (4g, 10.85 mmol), DIEA (5.48 mL, 31.5 mmol), and DMF (35 mL). Silica column (0-5% MeOH/DCM, 0.5% formic acid) to yield (3.9 g, 66% yield). LC-MS t_R: 5.23 min, m/z 537.42 [M+1H], $C_{30}H_{38}N_2O_7$, Calcd. 537.27 [M+1H]

Isothiocyanate Synthesis

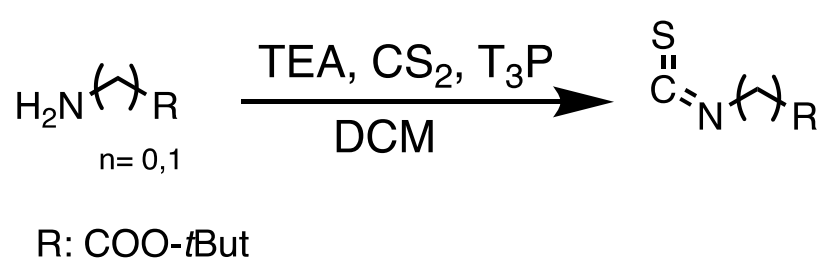

Inhibitor Synthesis

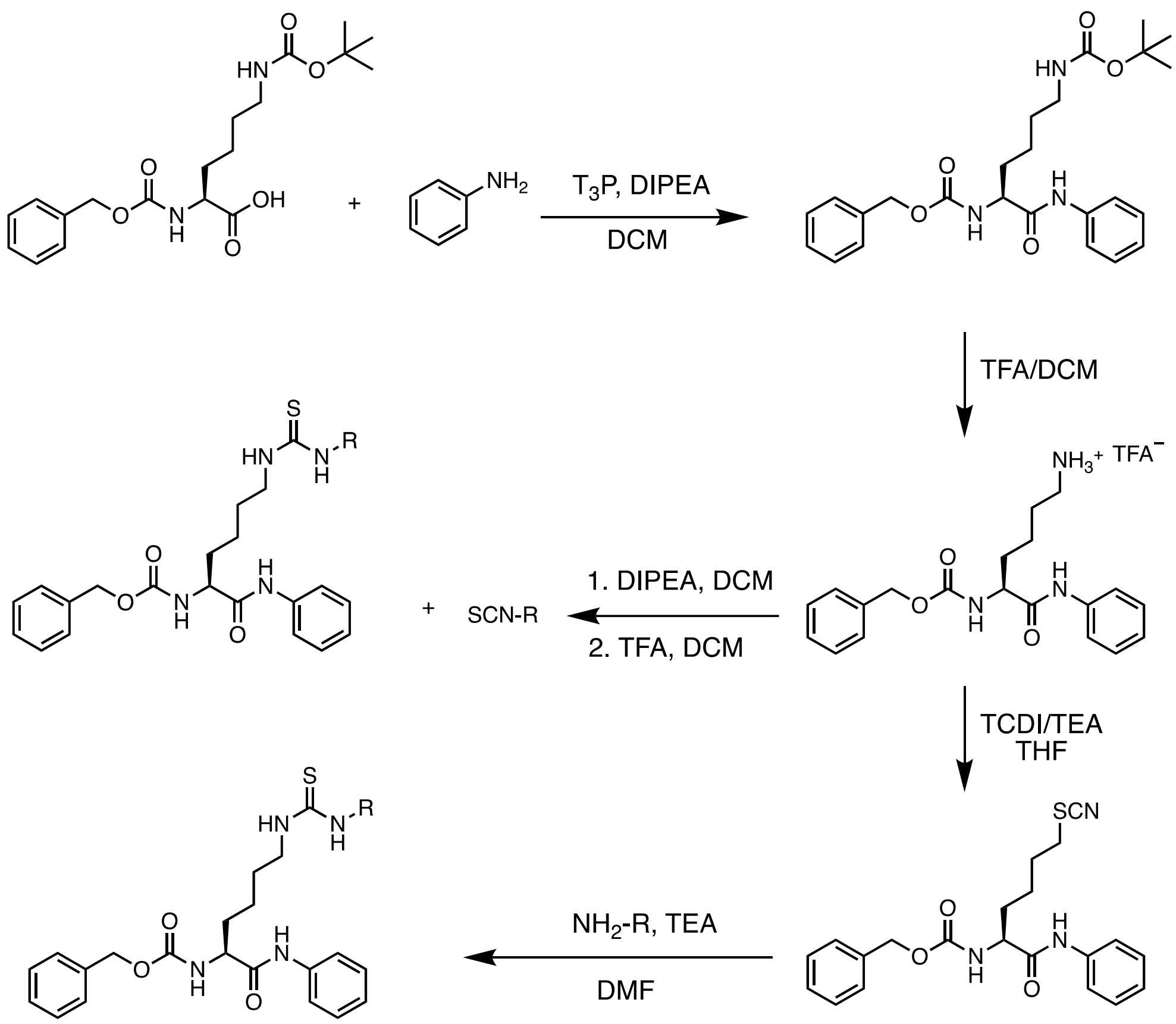

Figure S9. *Isothiocyanate synthesis*: Either t-butyl glycinate or β-alanine t-butyl ester hydrochloride (1 eq) in DCM, triethylamine (5 eq), and carbon disulfide (3 eq) stirred for 1 hour at RT, cooled to 0°C for the addition of propanephosphonic anhydride (T3P, 1.8 eq), and reacted to completion. *Inhibitor synthesis*: Cbz-Lys(Boc)-OH (1 eq), aniline (1 eq), T3P (2 eq), and diisopropylethylamine (DIEA, 3 eq) in DCM. Then, boc deprotection of Cbz-Lys(boc)-aniline (1 eq) in a 25% trifluoroacetic acid in DCM solution. The TFA salt of Cbz-Lys-Aniline (1 eq), can be coupled with a prepared isothiocyanate (1.1 eq) with DIEA (2 eq) in DCM, followed by removal of the tert-butyl group of the thiourea in 25% TFA in DCM to produce the desired acid. Alternately, TFA salt of Cbz-Lys-phenylamide can be reacted with 1,1′-thiocarbonyl diimidazole (1.2 eq.) and triethylamine (2.5 eq.) in THF. Direct reaction with the isothiocyanate with a free amine (1.5 eq) in the presence of triethylamine (2 eq.) in DMF afforded the desired product which was purified by semi-preparative HPLC.

Benzyl tert-butyl (6-oxo-6-(phenylamino)hexane-1,5-diyl)(S)-dicarbamate (Cbz-Lys(Boc)-Aniline). Cbz-Lys(Boc)-OH (500 mg, 1.31 mmol) was added to a round bottom flask and purged with Ar(g) and dissolved in anhydrous DCM (25 mL). Subsequently, DIPEA 687 µL, 3.94 mmol) and aniline (120 µL, 1.31 mmol) were added to the solution followed by the dropwise addition of 50% T3P in ethyl acetate (1.56 mL, 2.63 mmol) to the flask. Following two hours, or until HPLC showed depletion of starting material the reaction was quenched with H_2O (20 mL), followed by washing of the organic layer with H_2O (4x, 20 mL), 0.5 M HCl (1 x 15 mL), and brine (2 x 20 mL). The organic extract was dried over anhydrous $NaSO_4$, filtered, and concentrated under reduced pressure and the off-white solid was moved directly to the next step without purification. LC-MS t_R: 5.7 min, m/z 356.1 [M+1H, -Boc], $C_{25}H_{33}N_3O_5$, Calcd. 456.24 [M+1H], 356.19 [M+1, -Boc]

Benzyl (S)-(6-amino-1-oxo-1-(phenylamino)hexan-2-yl)carbamate (Cbz-Lys-Aniline • TFA). Cbz-Lys(Boc)-phenylamide (482 mg, 1.06 mmol) was added to a flask and dissolved in DCM (20 mL) under Ar(g) followed by the addition of 25% v/v trifluoroacetic acid (TFA), 6.5 mL). The reaction was stirred for 4 hours, or until deemed complete by TLC and LC-MS at which time solvent was removed under reduced pressure. This intermediate was moved to the next step without further purification. LC-MS t_R: 3.39 min, m/z 356.1 [M+1H], $C_{20}H_{25}N_3O_3$, Calcd. 356.19 [M+1H]

Benzyl (S)-(1-oxo-1-(phenylamino)-6-thiocyanatohexan-2-yl)carbamate (Cbz-Lys(SCN)-phenylamide. Cbz-Lys-phenylamide • TFA (150 mg, 0.42 mmol) was added to a vial and purged with $N_{2(g)}$ followed by the addition of anhydrous THF (6 mL). To this solution, solid 1,1′-thiocarbonyl diimidazole (90 mg, 0.51 mmol) and triethylamine (TEA, 145 uL, 1.05 mmol) were added and the reaction was allowed to proceed overnight at RT. The crude reaction mixture was concentrated under reduced pressure and dissolved in ethyl acetate (40 mL) followed by washing with H_2O (15 mL, 3x), brine (15 mL, 1x). The collected organic layer was dried over Na_2SO_4 and concentrated by rotary evaporation to yield an off-white solid which was moved directly to the next step without further purification.

Benzyl (*S*)-(6-(3-methylthioureido)-1-oxo-1-(phenylamino)hexan-2-yl)carbamate (1). Cbz-Lys(SCN)-phenylamide (50 mg, 0.125 mmol) was added to a vial and purged with $N_{2(g)}$ followed by the addition of anhydrous N-N-dimethylformamide (DMF) (1 mL). To this solution, methylamine (33% (w/w) in absolute EtOH, (17.5 µL, 0.18 mmol) and TEA (35 µL, 0.25 mmol) was added at 0ºC. The reaction was allowed to warm to room temperature and proceed overnight at RT at which time it was diluted in H_2O and extracted with ethyl acetate (3x, 20 mL) and the organic layer was washed brine (15 mL, 2x), dried over dried over Na_2SO_4 and concentrated by rotary evaporation. The crude material was dissolved in a small volume of DMF and subjected to semi-preparative HPLC yielding the pure title compound as a white solid (47.2 mg, 92% yield). LC-MS t_R: 4.80 min, m/z 429.1 [M+1H], $C_{22}H_{28}N_4O_3S$, Calcd. 429.19 [M+1H] ^{1}H NMR (400 MHz, DMSO) δ 1.26 – 1.60 (m, 4H), 1.64 (dt, *J* = 14.2, 6.5 Hz, 2H), 2.64 – 2.93 (m, 3H), 4.13 (q, *J* = 7.8 Hz, 1H), 5.03 (s, 2H), 6.93 – 7.14 (m, 1H), 7.14 – 7.45 (m, 8H), 7.55 (dd, *J* = 34.1, 7.9 Hz, 3H), 9.96 (s, 1H).

Benzyl (S)-(6-(3-dodecylthioureido)-1-oxo-1-(phenylamino)hexan-2-yl)carbamate. (2) Cbz-Lys(SCN)-phenylamide (50 mg, 0.125 mmol) was added to a vial and purged with $N_{2(g)}$ followed by the addition of anhydrous DMF (1 mL). To this solution, dodecylamine (35 mg, 0.19 mmol) and TEA (35 µL, 0.25 mmol) was added at 0ºC. The reaction was allowed to warm to room temperature and proceed overnight at RT at which time it was diluted in H_2O and extracted with ethyl acetate (3x, 20 mL) and the organic layer was washed brine (15 mL, 2x), dried over dried over Na_2SO_4 and concentrated by rotary evaporation. The crude material was purified silica column chromatography (30-50% ethyl acetate/hexanes) which following concentration under reduced pressure yielded 48.6 mg, 69% yield as a white solid. LC-MS t_R: 7.6 min, m/z 583.3 [M+1H], $C_{33}H_{50}N_4O_3S$, Calcd. 583.36 [M+1H] ^{1}H NMR (400 MHz, DMSO) δ 0.73 – 0.96 (m, 3H), 1.24 (s, 18H), 1.39 – 1.78 (m, 6H), 4.13 (d, J = 6.1 Hz, 1H), 5.03 (s, 2H), 7.04 (t, J = 7.4 Hz, 1H), 7.17 – 7.44 (m, 8H), 7.50 (d, J = 7.9 Hz, 1H), 7.59 (d, J = 8.0 Hz, 2H), 9.96 (s, 1H).

***tert*-Butyl 2-isothiocyanatoacetate. t-butyl glycinate** (500 mg, 3.8 mmol) was added to a round bottom flask, purged with Ar(g), followed by the addition of DCM (25 mL). To this solution triethylamine (2.64 mL, 19 mmol) and carbon disulfide (0.69 mL, 11.4 mmol) were added followed by stirring the solution for 1 hour. The reaction mixture was then cooled to 0°C in an ice bath and 50% T3P in ethyl acetate (4.52 mL, 7.6 mmol) was added dropwise. The reaction was allowed to warm to room temperature and stirred for an additional two hours, or until TLC analysis showed reaction completion using a p-anisaldehyde staining. Following completion, the reaction mixture was quenched with 10 mL of water for 30 minutes to hydrolyze residual T3P and diluted with DCM (250 mL). The organic layer was washed with H_2O (2 x 20 mL), 1 M HCL (2 x 20 mL), H_2O (2 x 20 mL), saturated sodium bicarbonate (2 x 20 mL), H_2O (1 x 20 mL), and brine (1 x 20 mL). The organic extract was then dried over anhydrous NaSO4, filtered, and concentrated under reduced pressure. The crude residue was purified via silica column chromatography (2% ethyl acetate/hexanes) which following concentration yielded the title product as a pale yellow liquid (460 mg, 69%). ^{1}H NMR (400 MHz, $CDCl_3$) δ 1.44 (s, 3H), 4.03 (s, 1H). ^{13}C NMR (101 MHz, $CDCl_3$) δ 27.97, 47.06, 83.93, 138.21, 164.98.

***tert*-Butyl 3-thiocyanatopropanoate. β-alanine t-butyl ester hydrochloride** (1 g, 5.5 mmol) was added to a round bottom flask, purged with Ar(g), followed by the addition of DCM (50 mL). To this solution triethylamine (3.84 mL, 27.5 mmol) and carbon disulfide (0.99 mL, 16.5 mmol) were added followed by stirring the solution for 1 hour. The reaction mixture was then cooled to 0°C in an ice bath and 50% T3P in ethyl acetate (5.9 mL, 9.9 mmol) was added dropwise. The reaction was allowed to warm to room temperature and stirred for an additional two hours, or until TLC analysis showed reaction completion using a p-anisaldehyde staining. Following completion, the reaction mixture was quenched with 10 mL of water for 30 minutes to hydrolyze residual T3P and diluted with DCM (500 mL). The organic layer was washed with H_2O (2 x 40 mL), 1 M HCL (2 x 40 mL), H_2O (2 x 40 mL), saturated sodium bicarbonate (2 x 40 mL), H_2O (1 x 40 mL), and brine (1 x 40 mL). The organic extract was then dried over anhydrous $NaSO_4$, filtered, and concentrated under reduced pressure. The crude residue was purified via silica column chromatography (2% ethyl acetate/hexanes) which following concentration yielded the title product as a pale yellow liquid (863 mg, 83%). ^{1}H NMR (400 MHz, $CDCl_3$) δ 1.49 (s, 1H), 2.61 (dd, J = 7.2, 5.9 Hz, 1H), 3.75 (dd, J = 7.2, 5.9 Hz, 1H). ^{13}C NMR (101 MHz, $CDCl_3$) δ 28.02, 35.86, 41.00, 81.81, 132.08, 168.95.

(S)-((5-(((Benzyloxy)carbonyl)amino)-6-oxo-6-(phenylamino)hexyl)carbamothioyl)glycine (3). Cbz-Lys-phenylamide • TFA (50 mg, 0.14 mmol) was added to a round bottom flask, purged with Ar(g), followed by the addition of anhydrous DMF (1 mL) and N,N-diisopropylethylamine (DIEA) (49 µL, 0.28 mmol). Then, tert-butyl 2-isothiocyanatoacetate (27.2 mg, 0.16 mmol) was added to the flask followed by stirring overnight at RT. Subsequently, the reaction was diluted with H_2O, extracted with ethyl acetate (3 x 100 mL) and the combined organic layers were washed with brine (1 x 75 mL), dried over anhydrous $NaSO_4$ and concentrated under reduced pressure. The crude product was then dissolved in anhydrous DCM (2 mL) and 15% v/v TFA (0.3 mL). Following 1 hour, or upon completion as determined by LC-MS, the reaction was concentrated under reduced pressure. Purification via semi-preparative HPLC yielded (48.6 mg, 73.5% yield). LC-MS t_R: 4.59 min, m/z 473.1 [M+1H], $C_{23}H_{28}N_4O_5S$, Calcd. 473.18 [M+1H] ^1H NMR (400 MHz, DMSO) δ 1.24 – 1.43 (m, 2H), 1.49 (tq, J = 11.6, 6.1, 5.2 Hz, 2H), 1.58 – 1.72 (m, 2H), 3.32 (s, 2H, overlap with residual water), 4.13 (d, J = 5.7 Hz, 3H), 5.04 (s, 2H), 7.04 (t, J = 7.4 Hz, 1H), 7.23 – 7.45 (m, 8H), 7.51 (d, J = 7.9 Hz, 1H), 7.59 (d, J = 8.0 Hz, 2H), 7.73 (t, J = 5.1 Hz, 1H), 9.97 (s, 1H), 12.51 (s, 1H).

(S)-3-Oxo-1-phenyl-5-(phenylcarbamoyl)-11-thioxo-2-oxa-4,10,12-triazapentadecan-15-oic acid (4). Cbz-Lys-phenylamide • TFA (50 mg, 0.14 mmol) was added to a round bottom flask, purged with Ar(g), followed by the addition of anhydrous DMF (1 mL) and N,N-diisopropylethylamine (DIEA) (49 µL, 0.28 mmol). Then, tert-butyl 3-thiocyanatopropanoate (29.3 mg, 0.155 mmol) was added to the flask followed by stirring overnight at RT. Subsequently, the reaction was diluted with H_2O, extracted with ethyl acetate (3 x 100 mL) and the combined organic layers were washed with brine (1 x 75 mL), dried over anhydrous $NaSO_4$ and concentrated under reduced pressure. The crude product was then dissolved in anhydrous DCM (2 mL) and 15% v/v TFA (0.3 mL). Following 1 hour, or upon completion as determined by LC-MS, the reaction was concentrated under reduced pressure. Purification via semi-preparative HPLC yielded (29.4 mg, 43% yield). LC-MS t_R: 4.63 min, m/z 487.1 [M+1H], $C_{24}H_{30}N_4O_5S$, Calcd. 487.19 [M+1H]

***N*^{2}-(((9*H*-Fluoren-9-yl)methoxy)carbonyl)-*N*^{6}-tetradecanethioyl-*L*-lysine (Fmoc-Lys(thiomyr)-OH)** A round bottom flask charged with Z-Lys(myr)-OH (1 g, 1.73 mmol) and Lawesson's reagent (630 mg, 1.56 mmol) was purged with $N_{2(g)}$ yielding positive pressure. Anhydrous THF (30 mL) was added and the reaction was allowed to react at RT overnight. The solvents were removed and the crude reaction mixture was purified by silica column (0.5% MeOH/DCM, 1% formic acid) to yield the title compound (250 mg, 24% yield). LC-MS t_R: 8.1 min, m/z 595.3 [M+1H], $C_{35}H_{50}N_2O_4S$, Calcd. 595.35 [M+1H] ^1H NMR (400 MHz, MeOD) δ 0.78 (t, J = 6.8 Hz, 3H), 1.09 – 1.25 (m, 19H), 1.27 – 1.44 (m, 2H), 1.44 – 1.71 (m, 5H), 1.77 (dddt, J = 16.8, 13.2, 8.4, 4.0 Hz, 1H), 2.47 (t, J = 7.6 Hz, 2H), 3.40 – 3.54 (m, 2H), 3.63 – 3.78 (m, 1H), 4.00 – 4.17 (m, 2H), 4.18 – 4.40 (m, 2H), 7.23 (dt, J = 30.5, 7.4 Hz, 4H), 7.55 (t, J = 7.1 Hz, 2H), 7.60 – 7.80 (m, 2H). ^{13}C NMR (101 MHz, MeOD) δ 13.08, 22.33, 23.04, 26.87, 29.07, 29.23, 29.31, 29.35, 29.39, 31.07, 31.67, 45.22, 45.73, 53.78, 54.51, 66.60, 119.55, 124.88, 126.79, 127.41, 141.20, 143.77, 143.90, 157.27, 174.45, 205.15.

Table S1. Mass spectral analysis and retention times of synthetically prepared peptides

Peptide	Observed (m/z)	Calculated (m/z)	t_R
ACSK617 formyl	816.4 [1+]	816.43 [1+]	3.00 min
ACSK617 acetyl	830.5 [1+]	830.44 [1+]	2.50 min
ACSK617 propionyl	844.6 [1+]	844.46 [1+]	2.62 min
ACSK617 butyryl	858.5 [1+]	858.48 [1+]	2.67 min
ACSK617 crotonyl	856.4 [1+]	856.46 [1+]	2.75 min
ACSK617 hexanoyl	886.5 [1+]	886.51 [1+]	3.26 min
ACSK617 octanoyl	914.5 [1+]	914.54 [1+]	3.89 min
ACSK617 decanoyl	942.5 [1+]	942.57 [1+]	4.26 min
ACSK617 dodecanoyl	970.7 [1+]	970.60 [1+]	4.32 min
ACSK617 myristoyl	998.8 [1+]	998.63 [1+]	14.90 min*
ACSK617 palmitoyl	1026.8 [1+]	1026.66 [1+]	15.53 min*
ACSK617 malonyl	874.6 [1+]	874.43 [1+]	2.51 min
ACSK617 succinyl	888.7 [1+]	888.45 [1+]	2.84 min
ACSK617 glutaryl	902.6 [1+]	902.47 [1+]	2.52 min
ACSK617 biotinoyl	1014.4 [1+]	1014.51 [1+]	2.67 min
ACSK617 thiomyristol	1014.5 [1+]	1014.61 [1+]	4.70 min

*Using a (Luna 5 um C18(2) 100 Å, 4.6 mm, 250 mm with the method as follows (30-52% ACN (20 mins), 52-100% ACN 30 min).

LCMS Spectra:
Note: In addition to the parent ion peak, a fairly prominent **-**108 m/z peak was observed in the MS spectra of Cbz-Lys(prop)-AMC, Cbz-Lys(but)-AMC, Cbz-Lys(mal)-AMC, Cbz-Lys(succ)-AMC, and Cbz-Lys(glu)-AMC when analyzed in negative mode. This -108 m/z ion likely corresponds to the fragmentation of the parent ion(s) and cleavage of the benzyl of the N-benzyloxycarbonyl (Cbz) group. Such cleavage has been proposed by Xu and colleagues,[6] in which fragmentation of Cbz containing molecules, under negative mode ionization, produces benzyl alcohol as a product as well as a substrate-based isocyanate, which can undergo rearrangement, followed by the loss of CO, to form a substrate-based aziridine ion in place of the endogenous Cbz group. This proposal is consistent with the loss of 108 m/z observed for these samples. Also, for the Cbz-Lys(acyl)-AMC substrates and the Fmoc-Lys(acyl)-OH molecules, non-covalent dimers are observed in some of the MS spectra.

Cbz-Lys(ac)-AMC

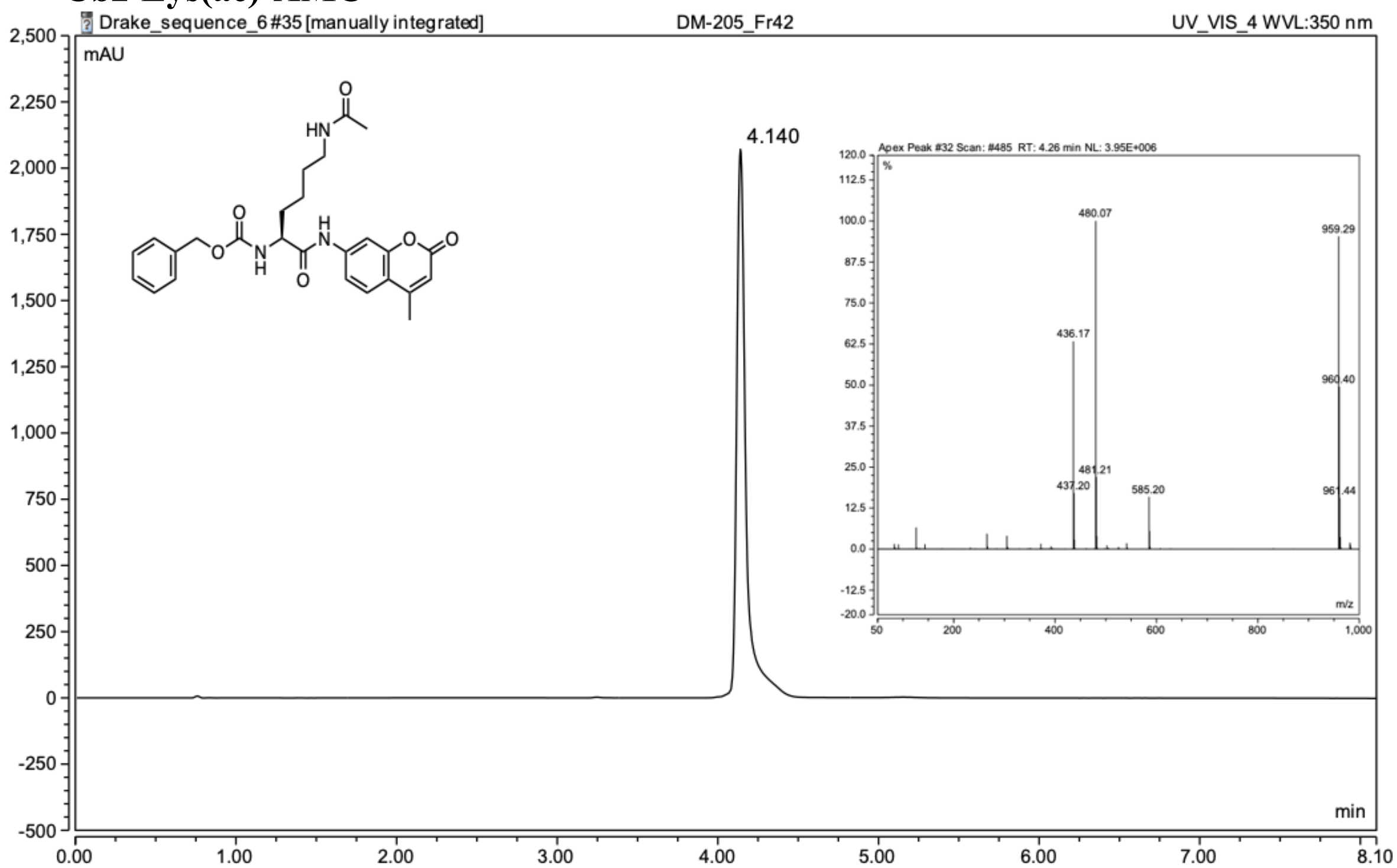

Cbz-Lys(prop)-AMC

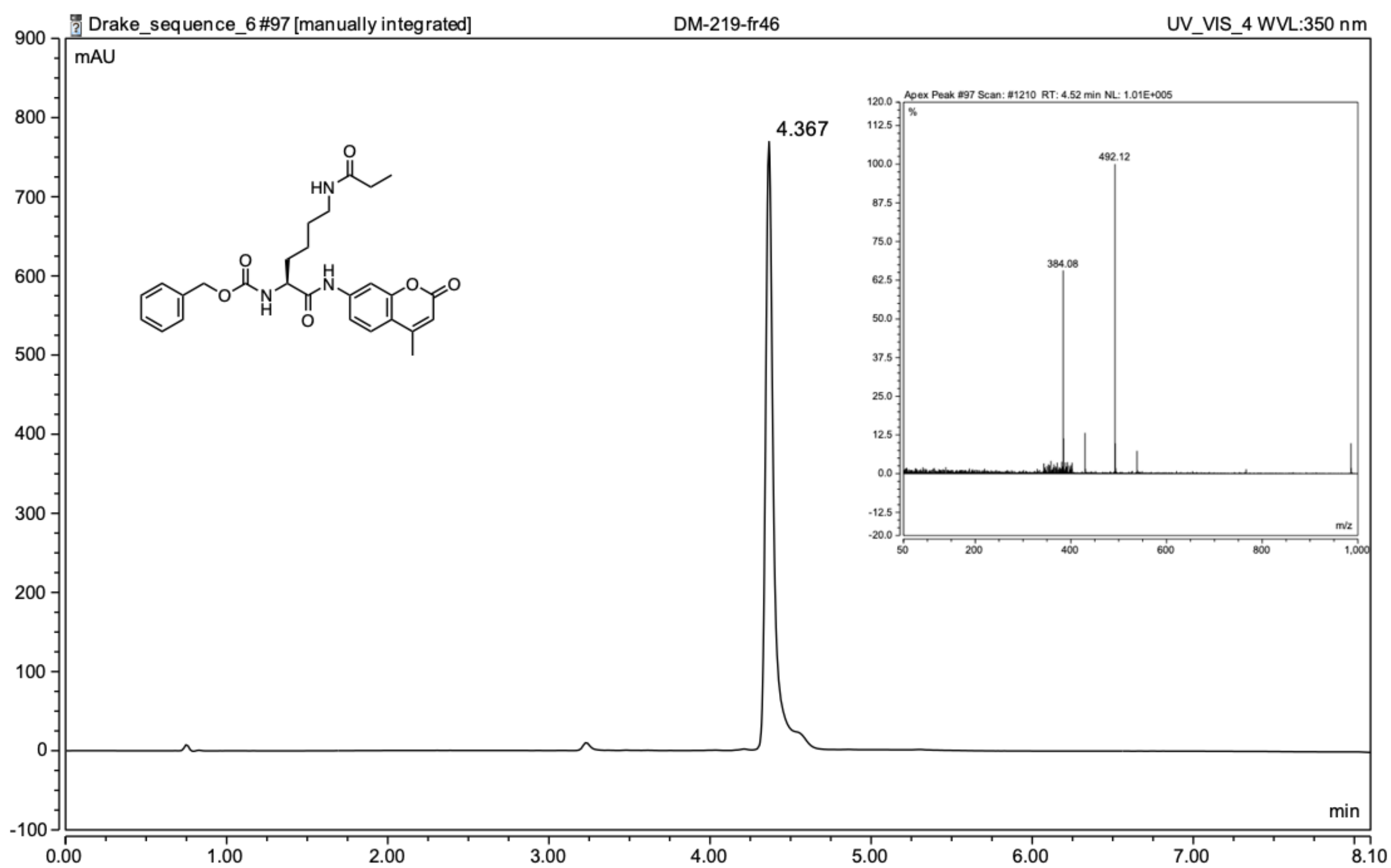

Cbz-Lys(but)-AMC

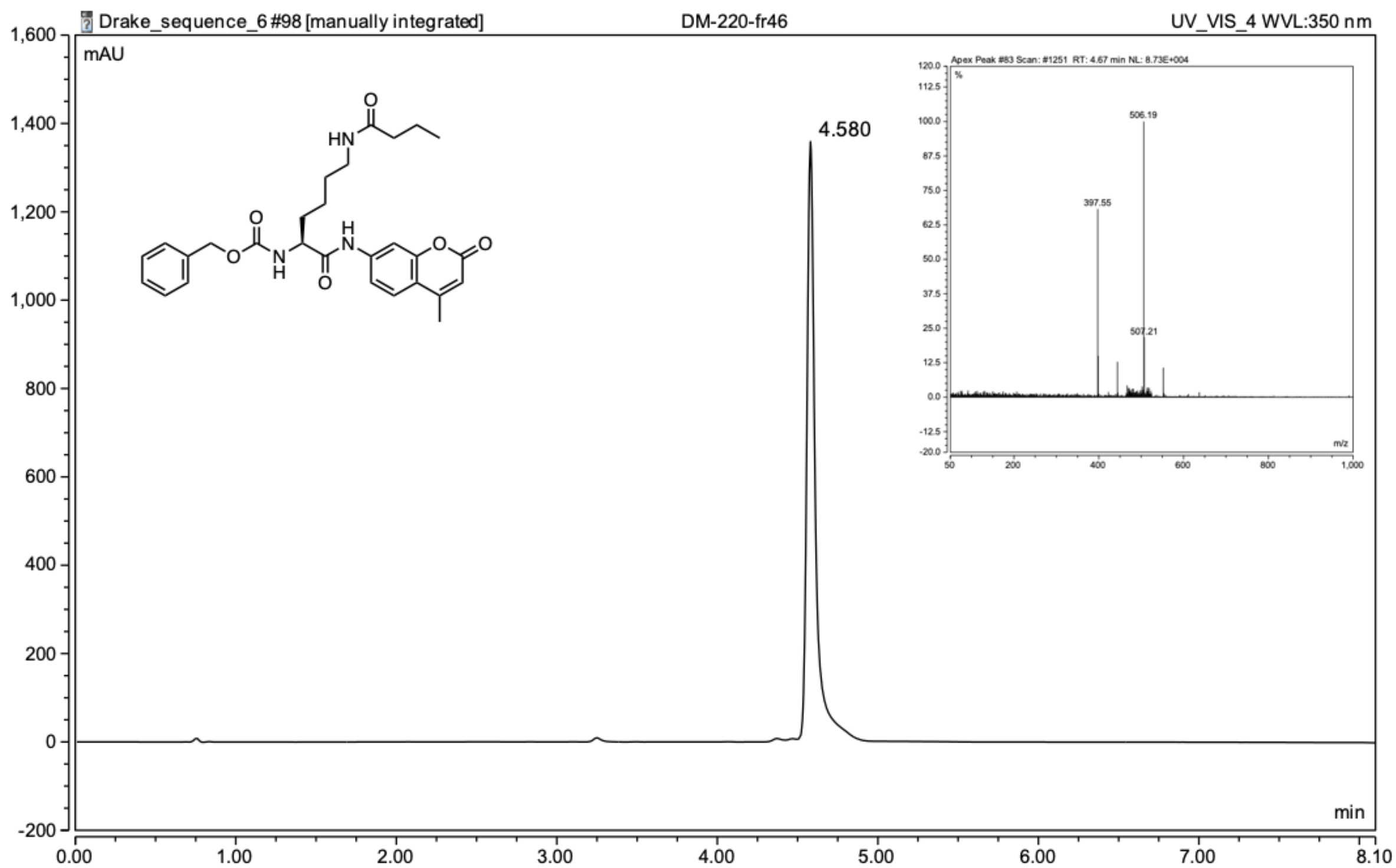

Cbz-Lys(mal)-AMC

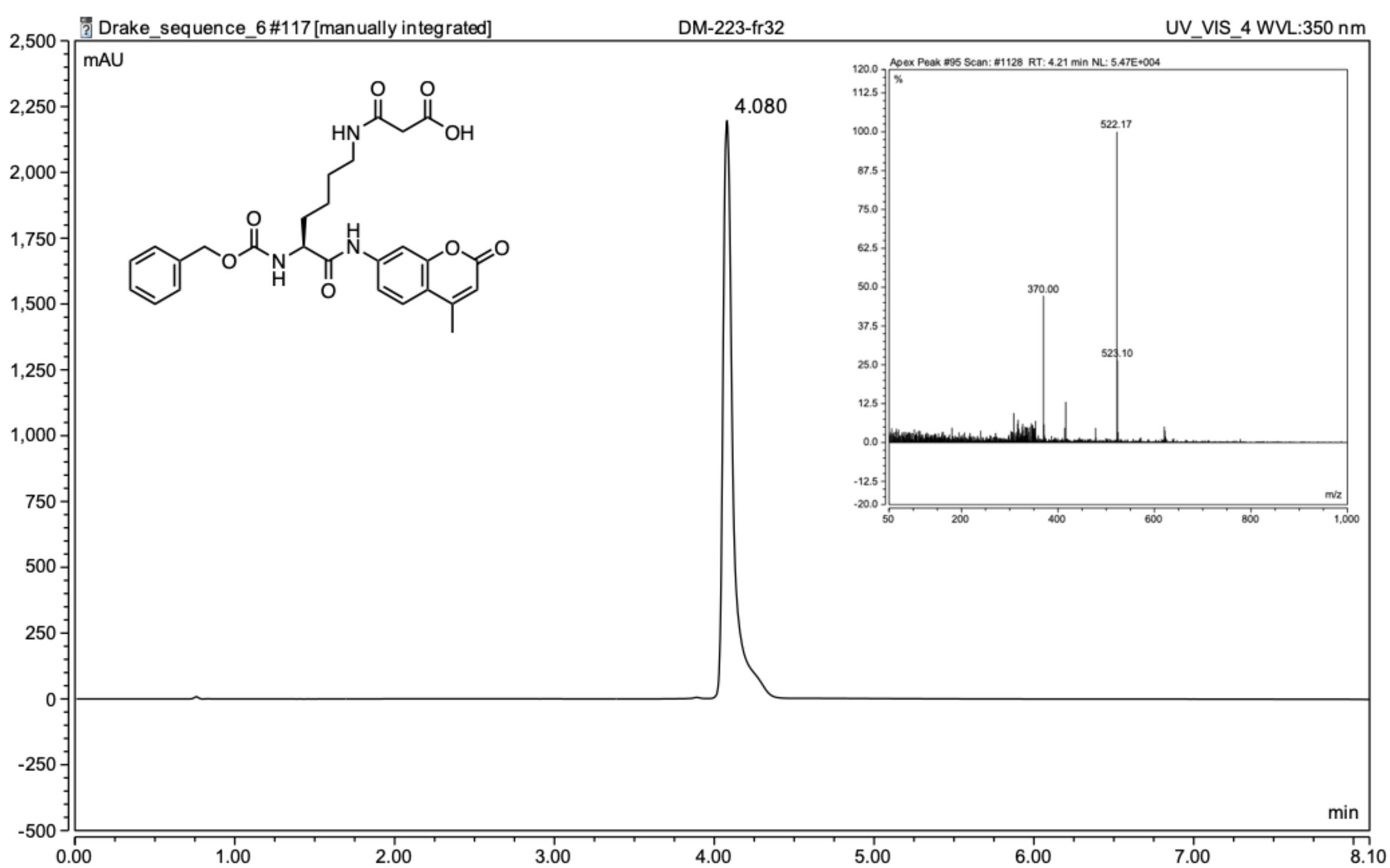

Cbz-Lys(succ)-AMC

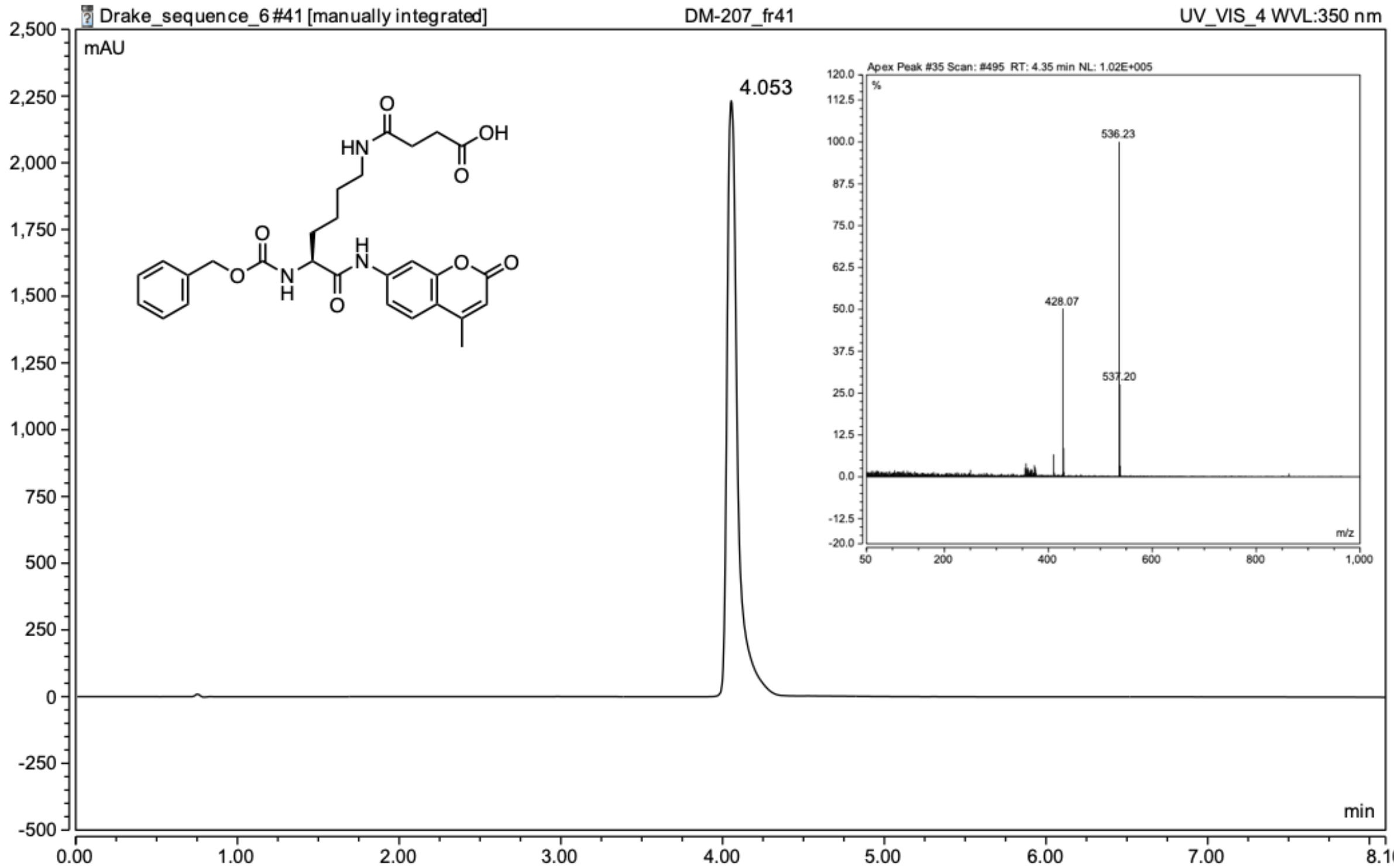

Cbz-Lys(glu)-AMC

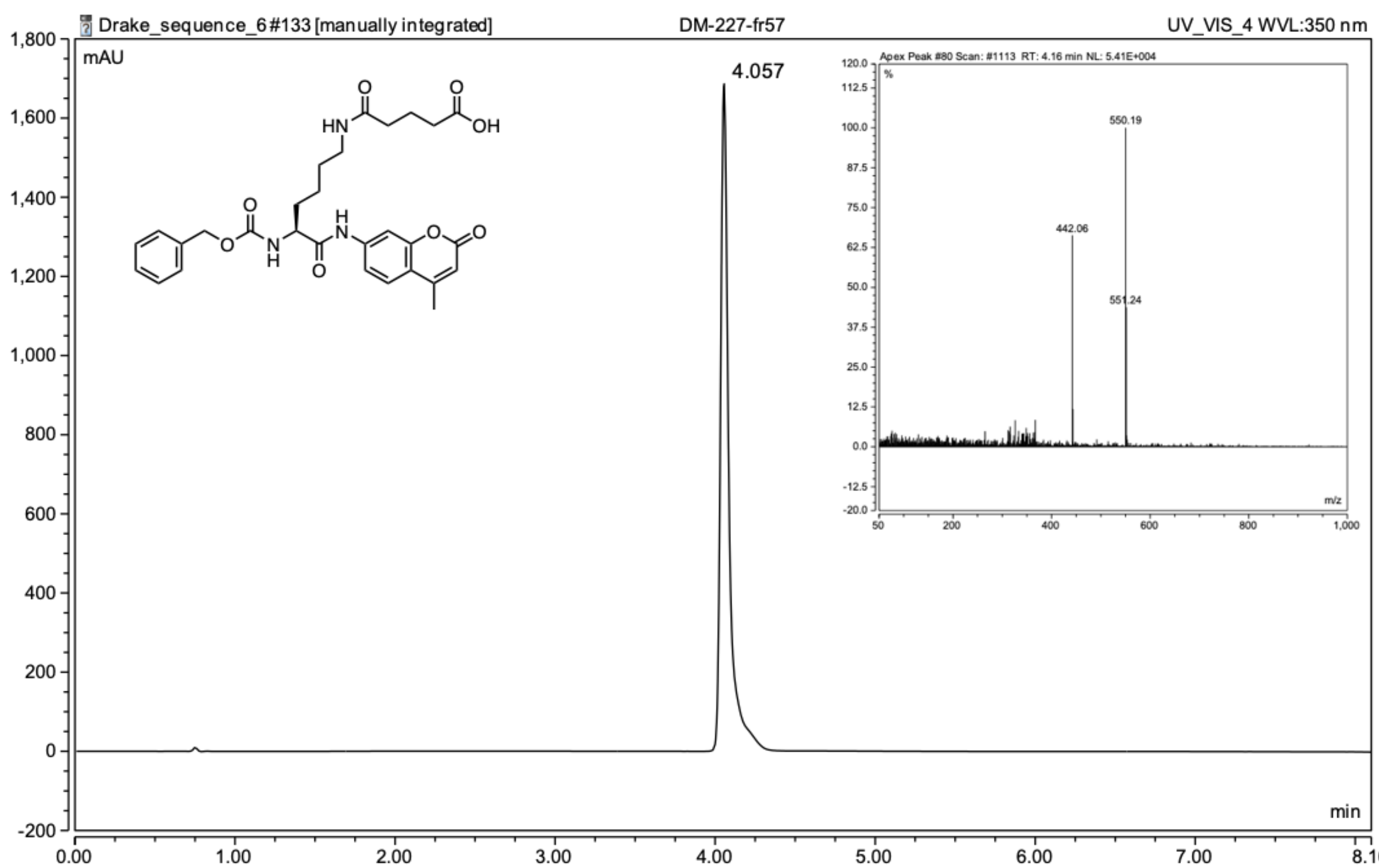

Fmoc-Lys(prop)-OH

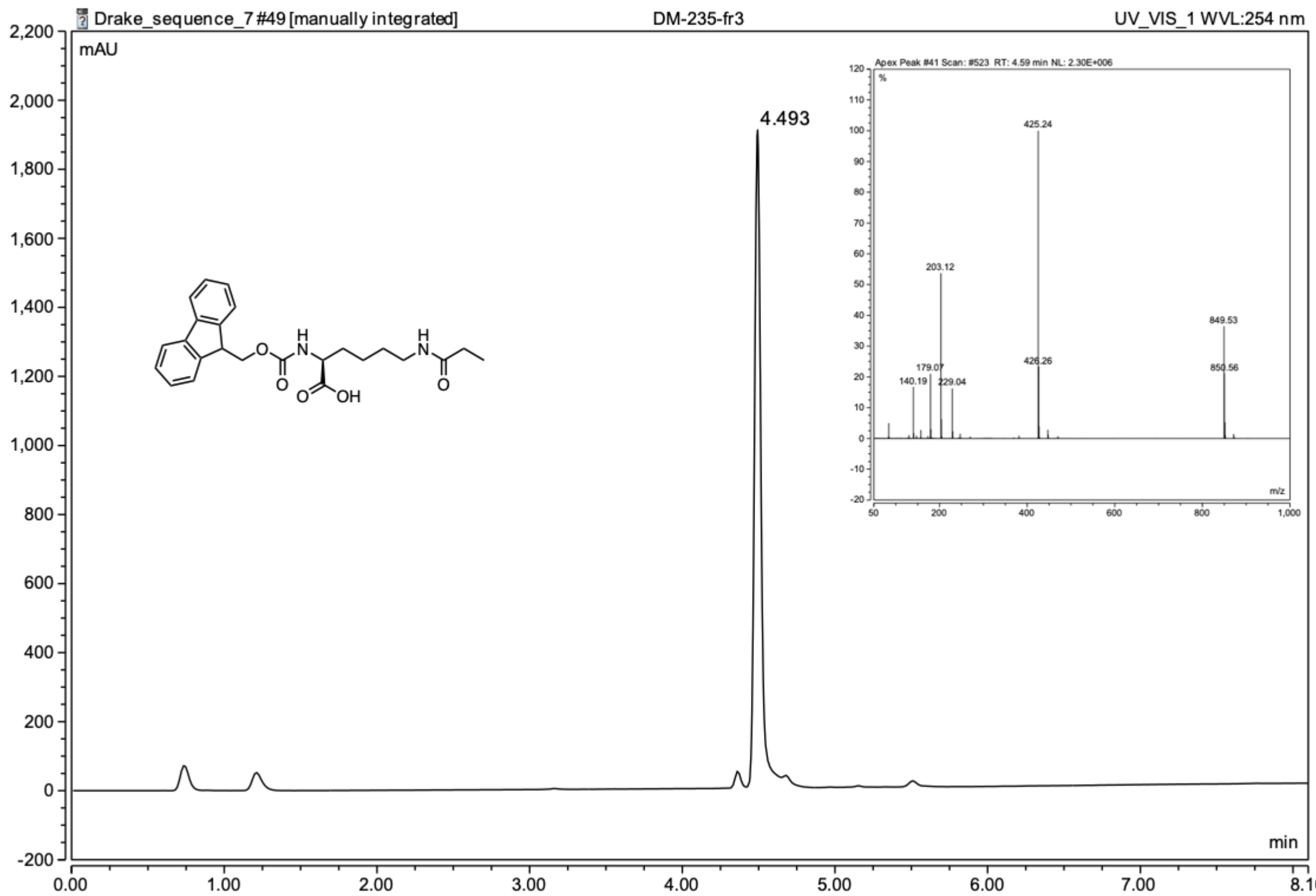

Fmoc-Lys(but)-OH

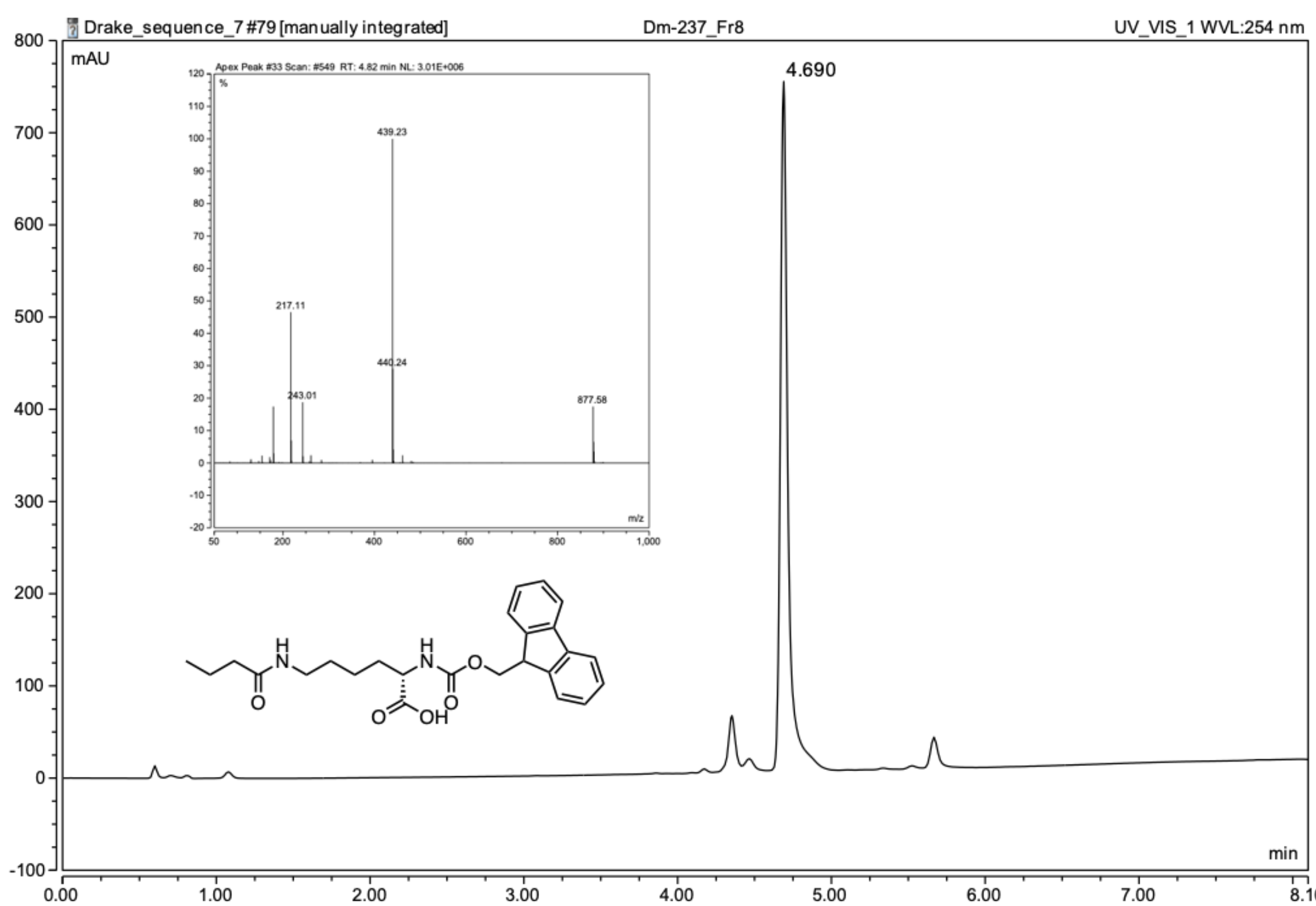

Fmoc-Lys(crot)-OH

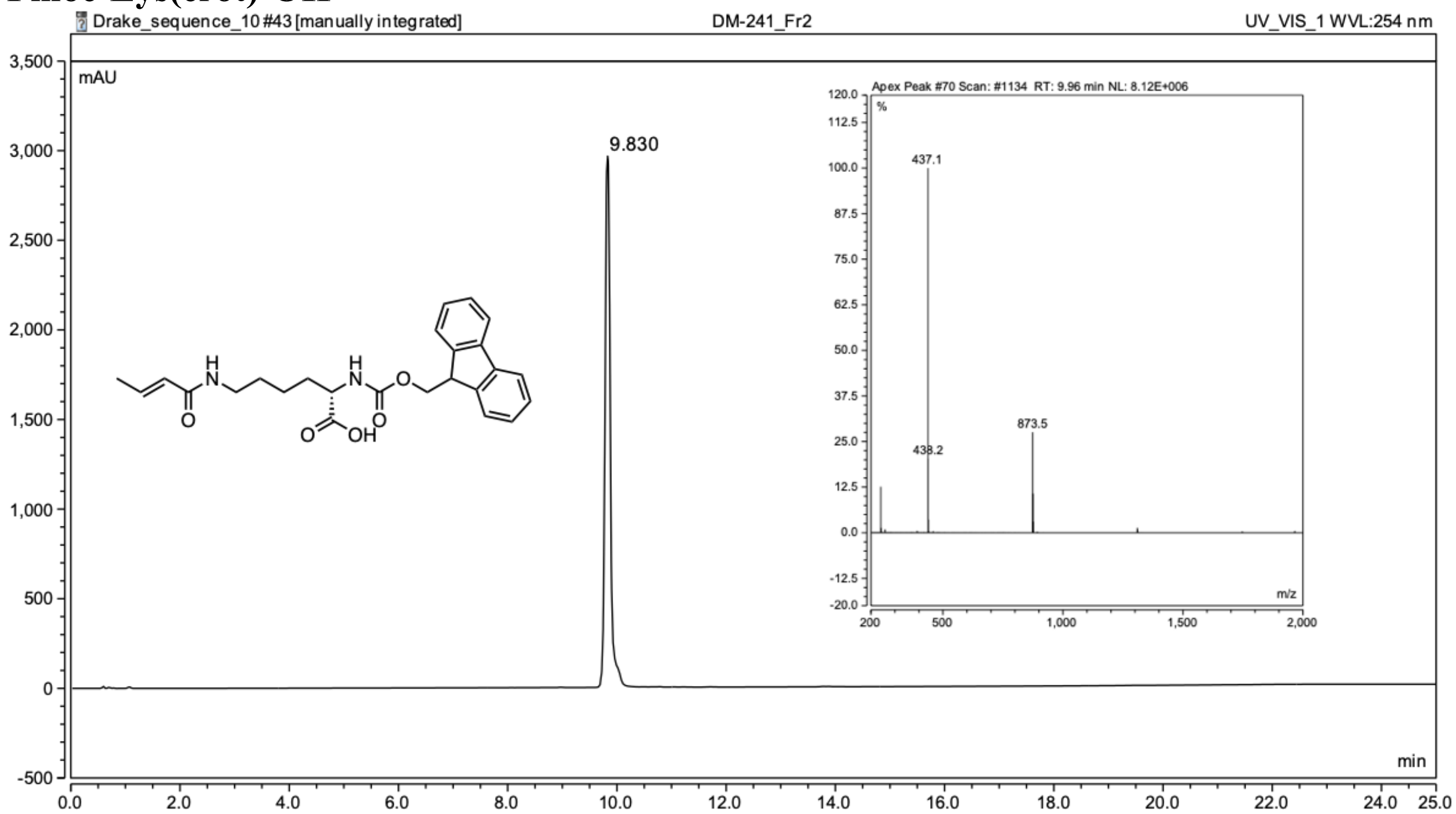

Fmoc-Lys(myr)-OH

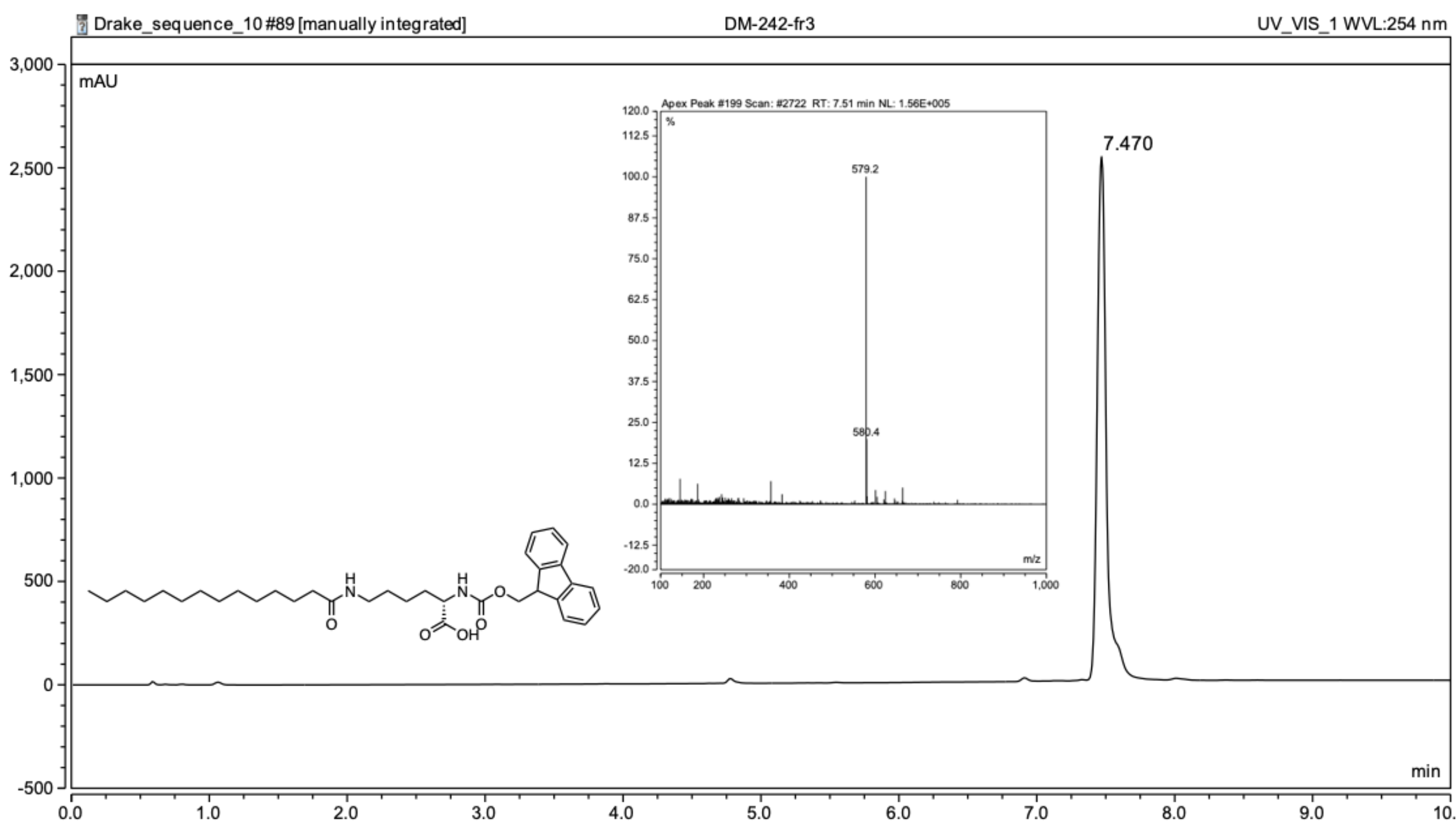

Fmoc-Lys(mal-O-*t*But)-OH

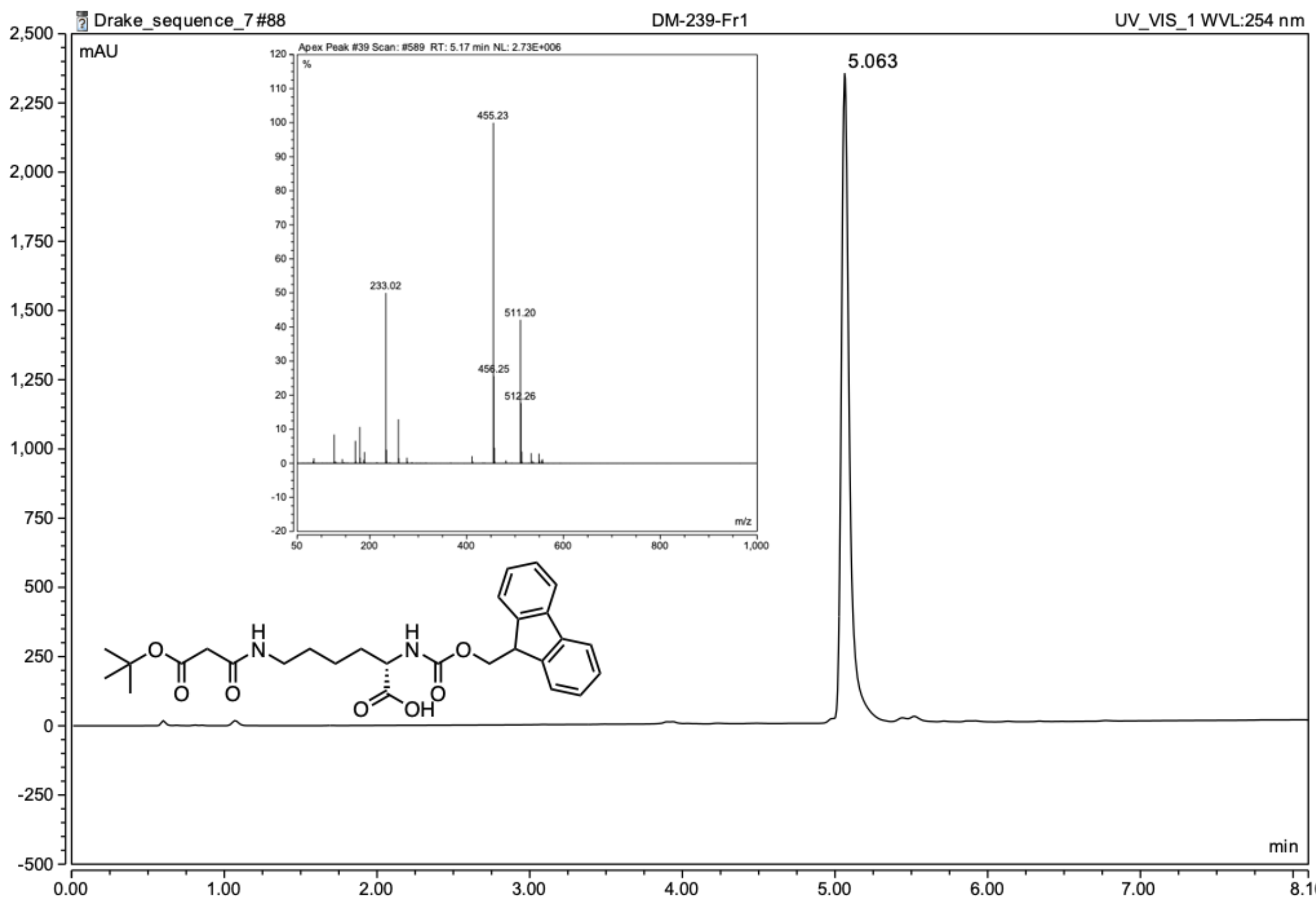

Fmoc-Lys(succ-O-*t*But)-OH

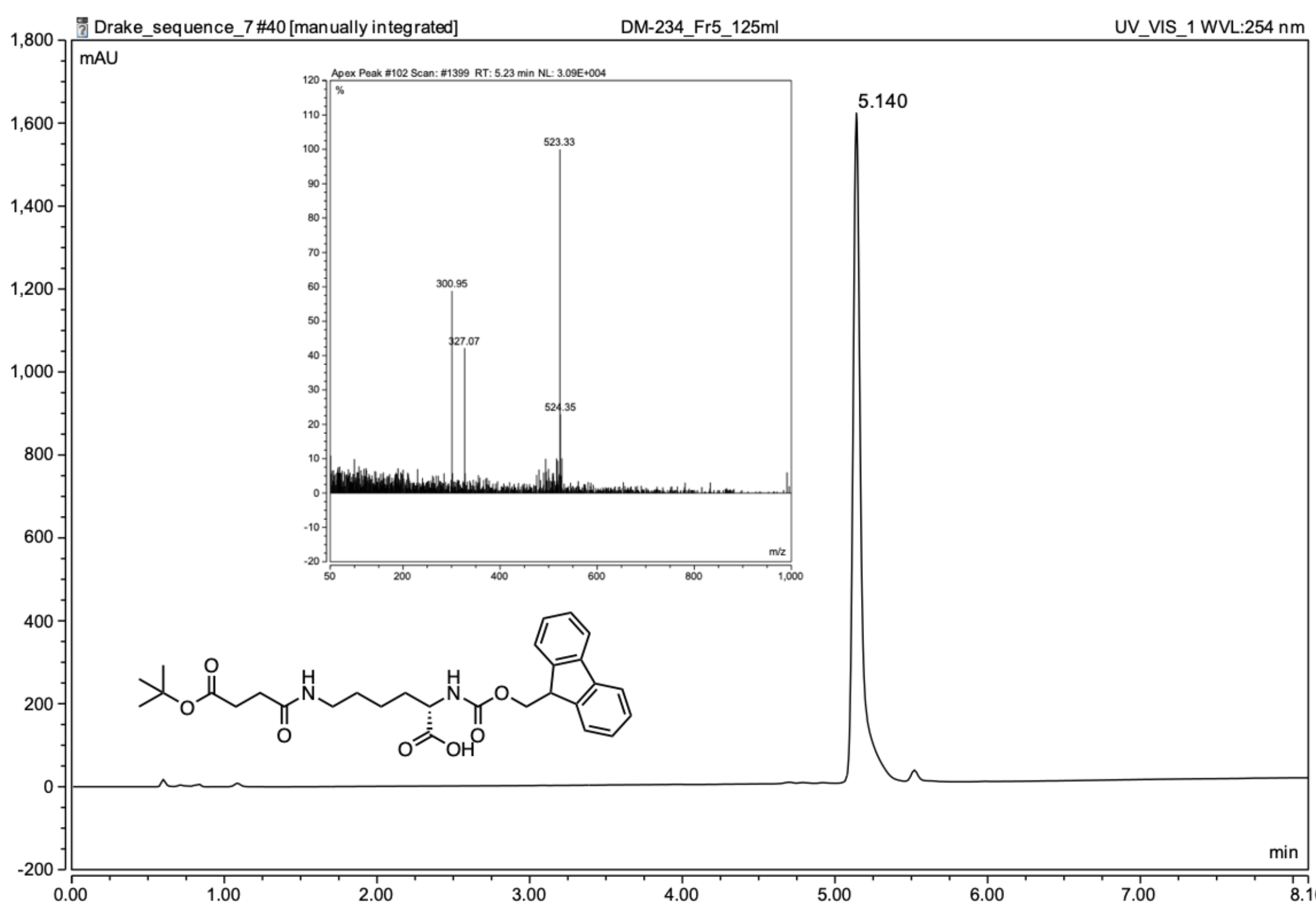

Fmoc-Lys(glut-O-*t*But)-OH

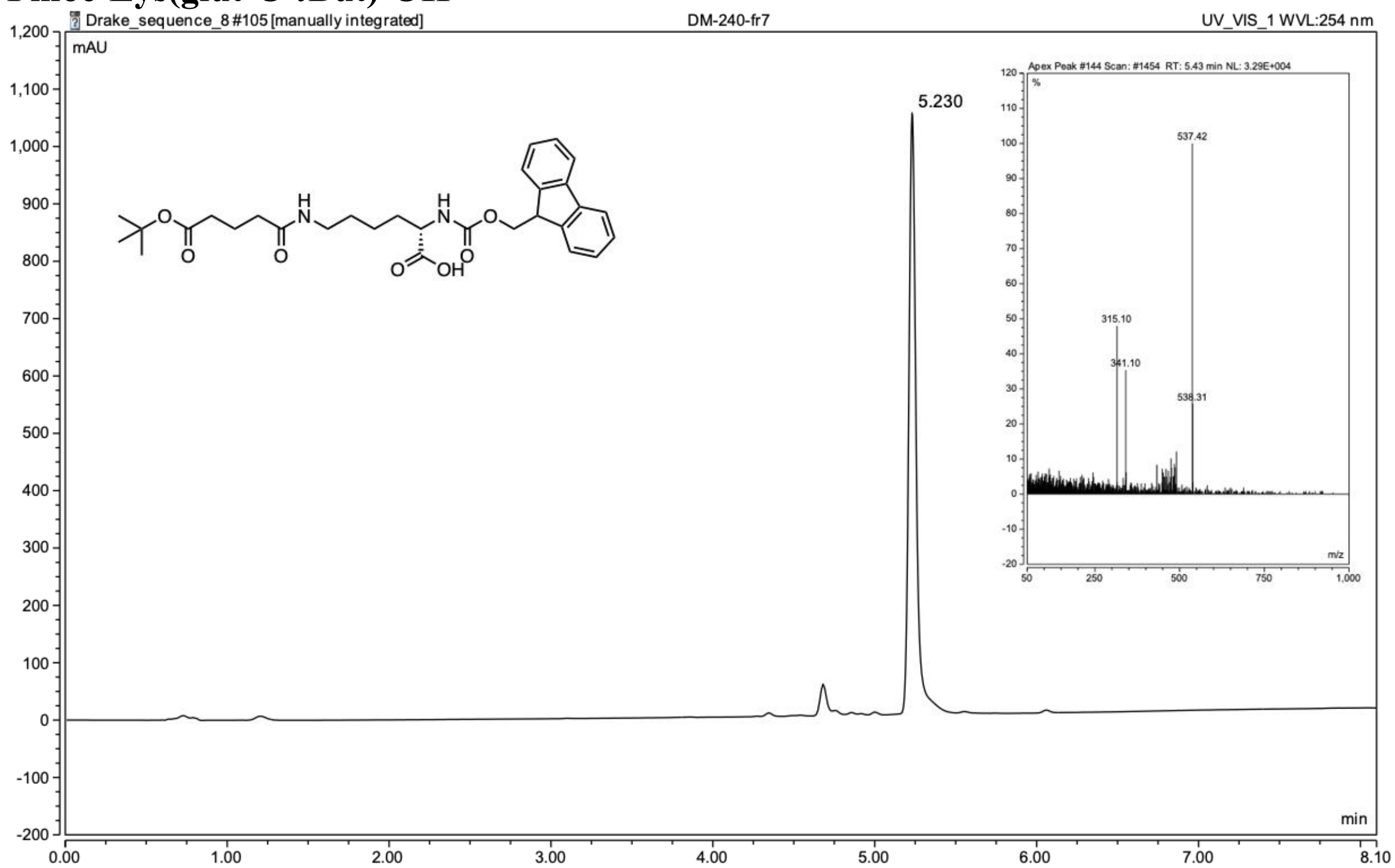

Cbz-Lys(boc)-aniline

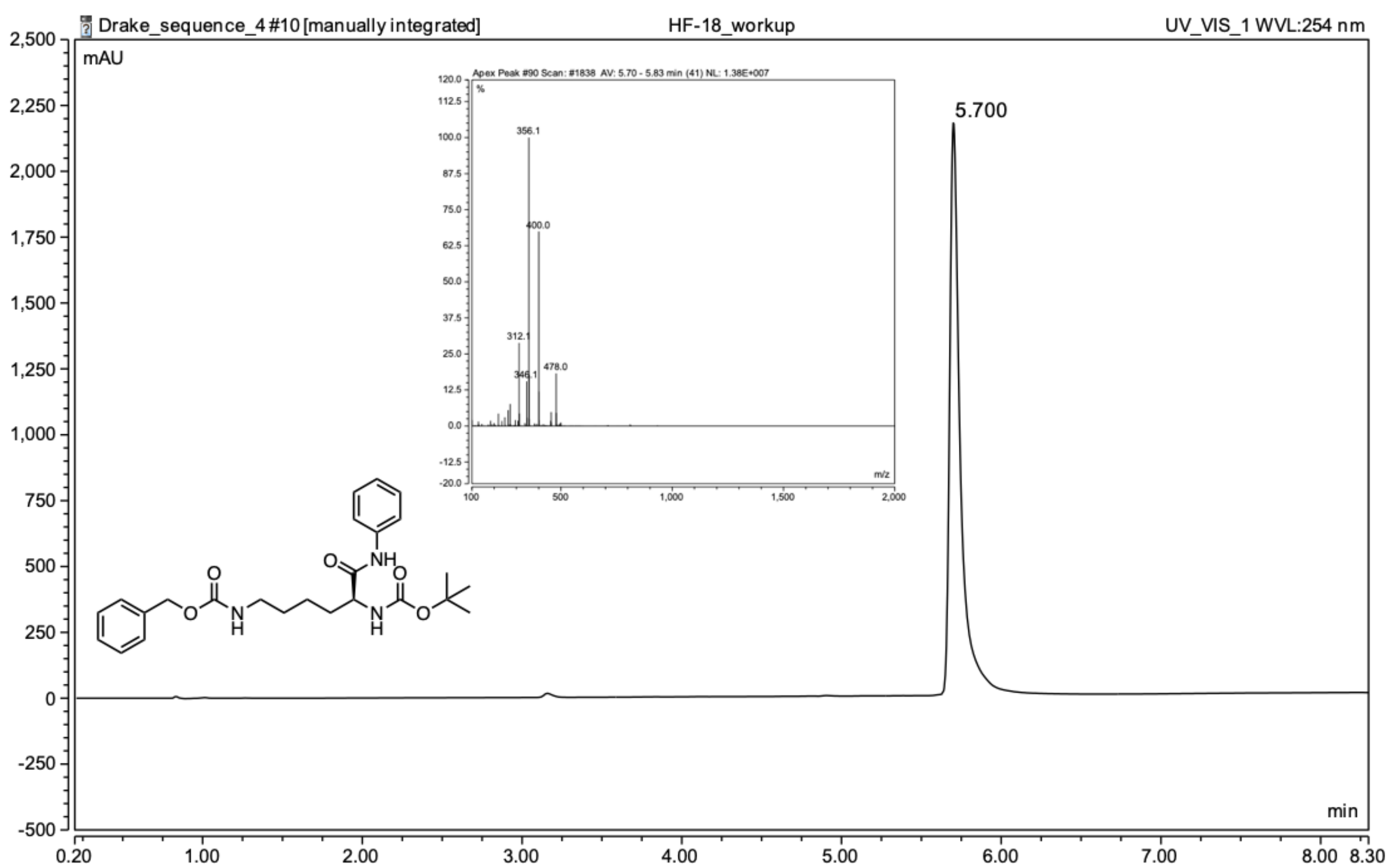

Cbz-Lys-Aniline • TFA

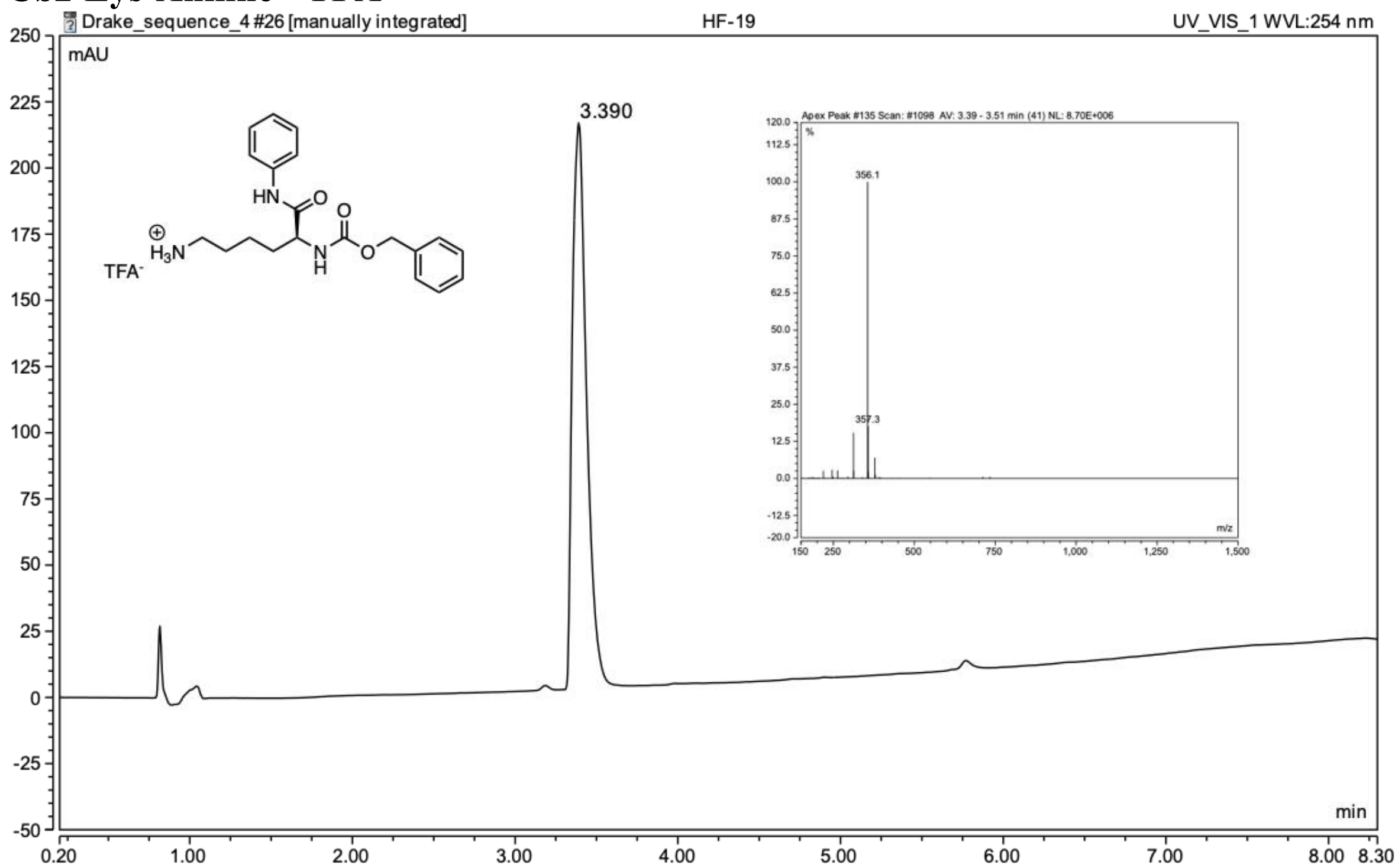

benzyl (*S*)-(6-(3-methylthioureido)-1-oxo-1-(phenylamino)hexan-2-yl)carbamate (1)

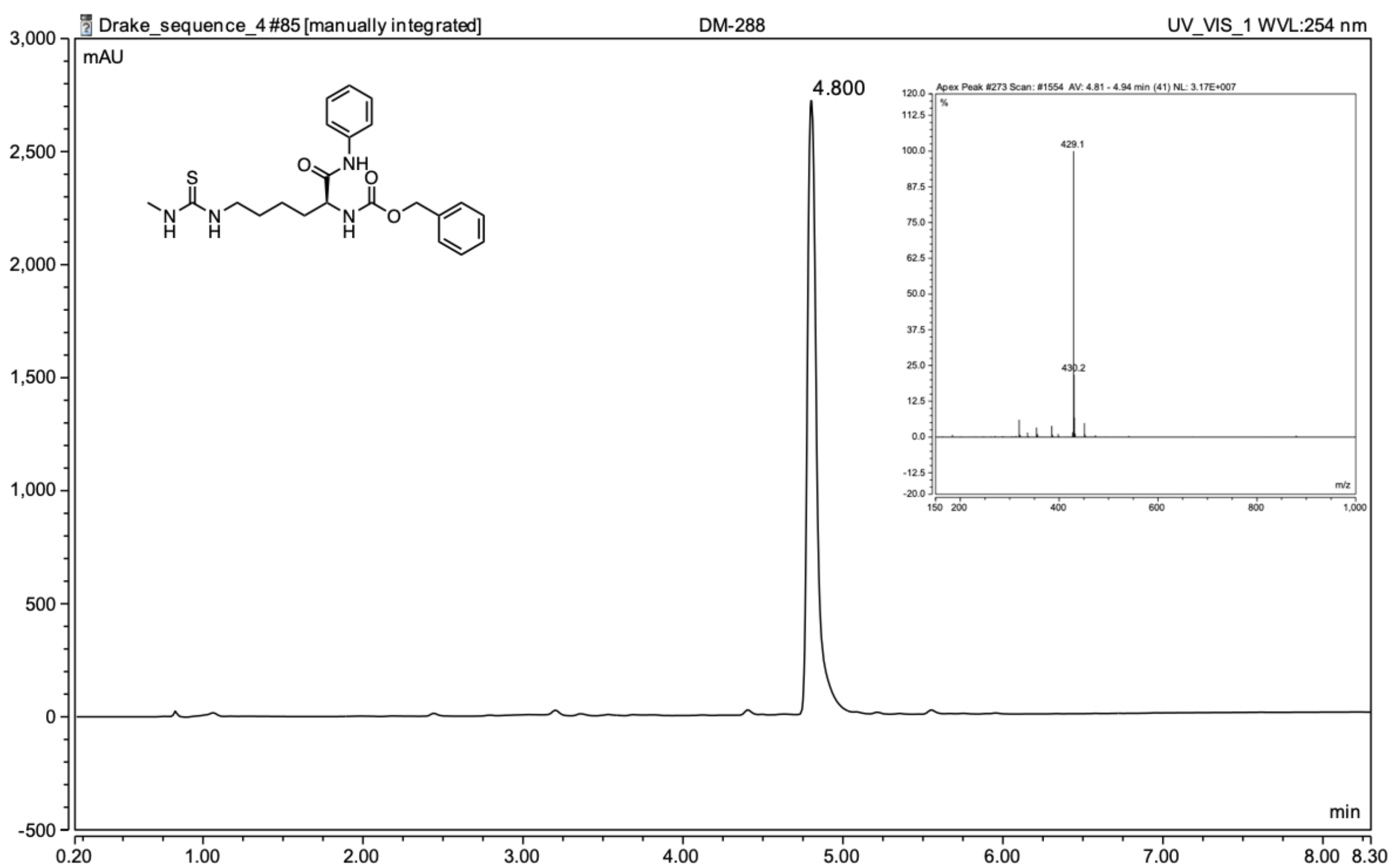

benzyl (S)-(6-(3-dodecylthioureido)-1-oxo-1-(phenylamino)hexan-2-yl)carbamate. (2)

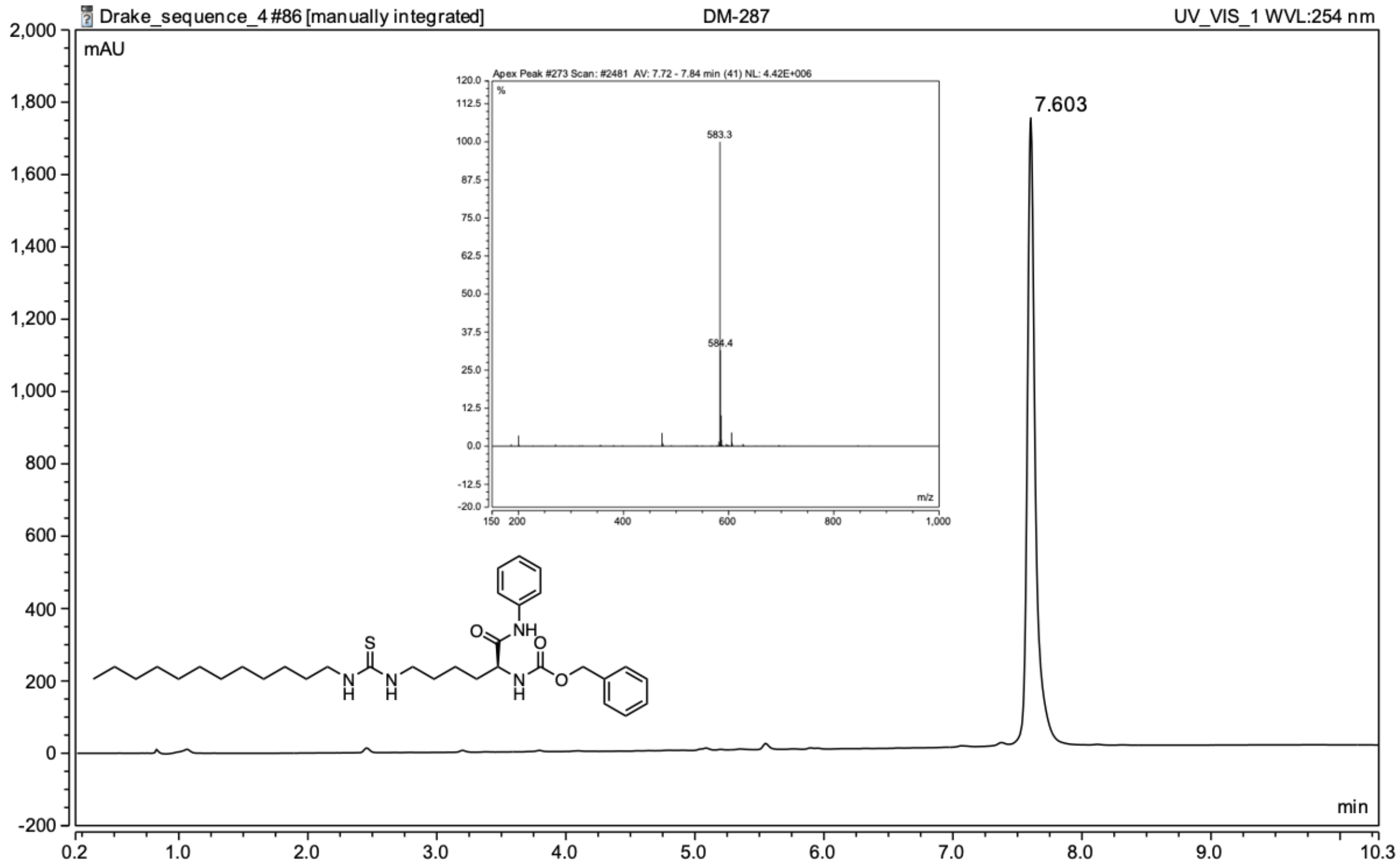

(S)-((5-(((benzyloxy)carbonyl)amino)-6-oxo-6-(phenylamino)hexyl)carbamothioyl)glycine (3).

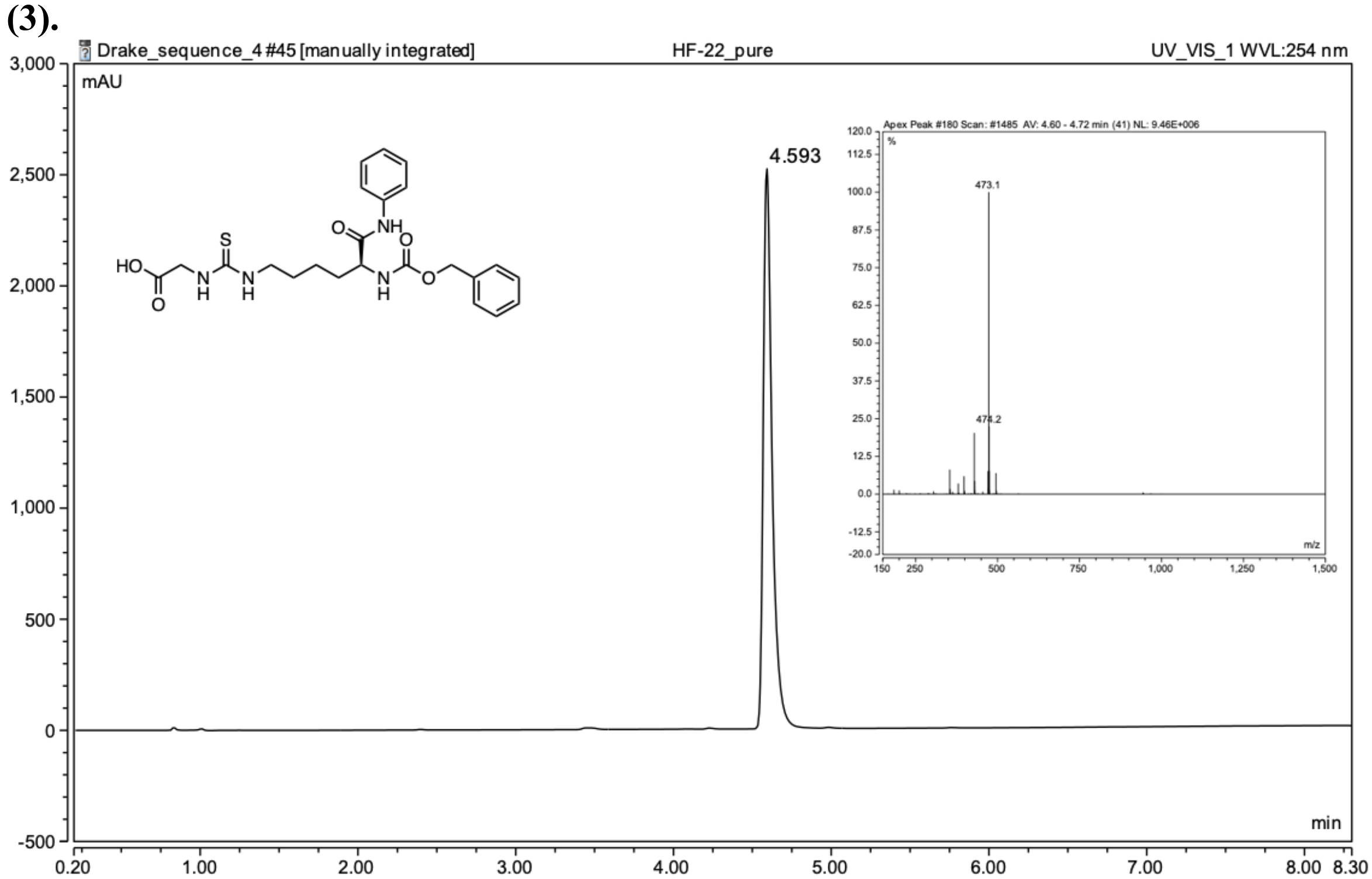

S)-3-oxo-1-phenyl-5-(phenylcarbamoyl)-11-thioxo-2-oxa-4,10,12-triazapentadecan-15-oic acid (4).

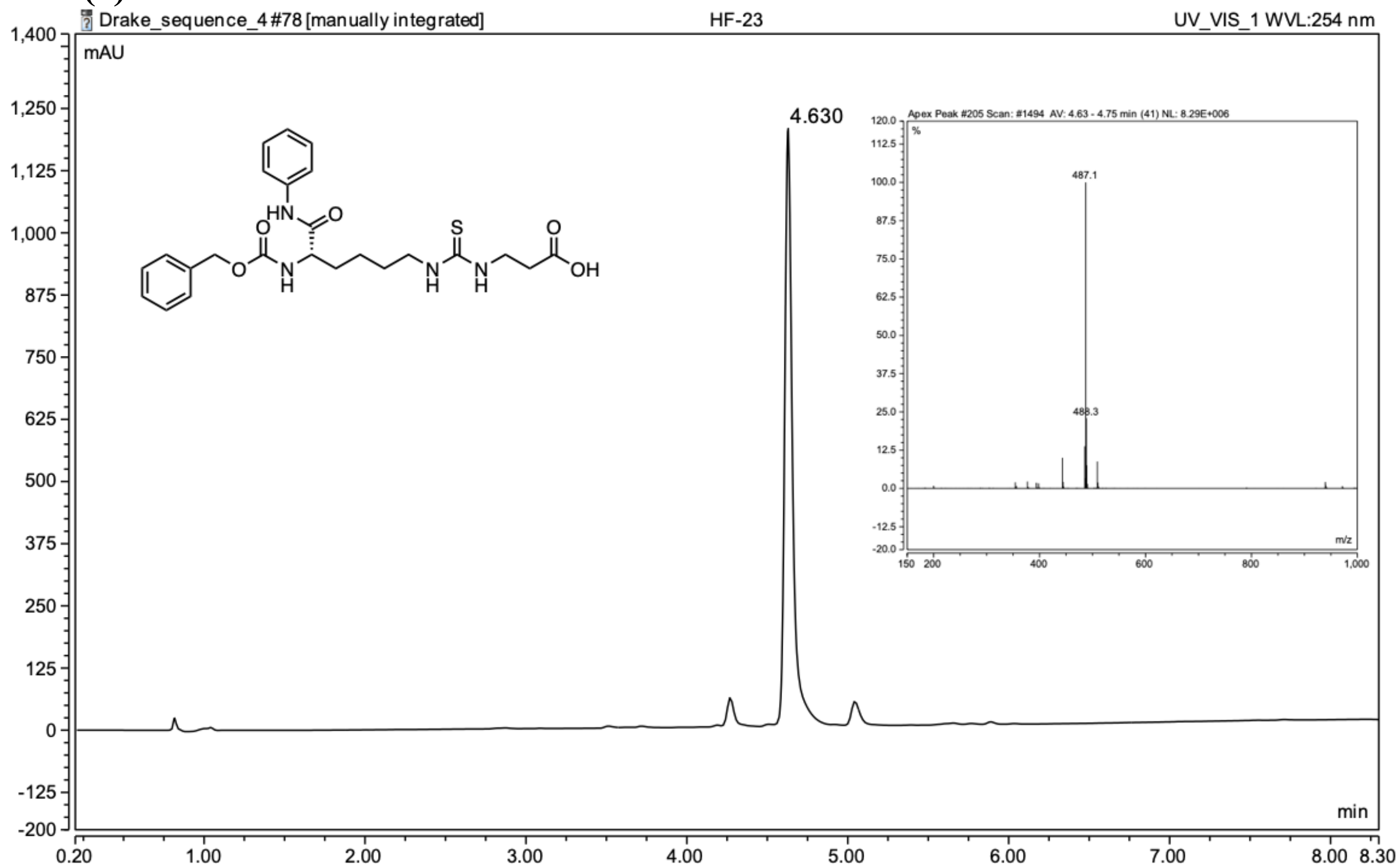

N

N^2-(((9*H*-fluoren-9-yl)methoxy)carbonyl)-N^6-tetradecanethioyl-*L*-lysine

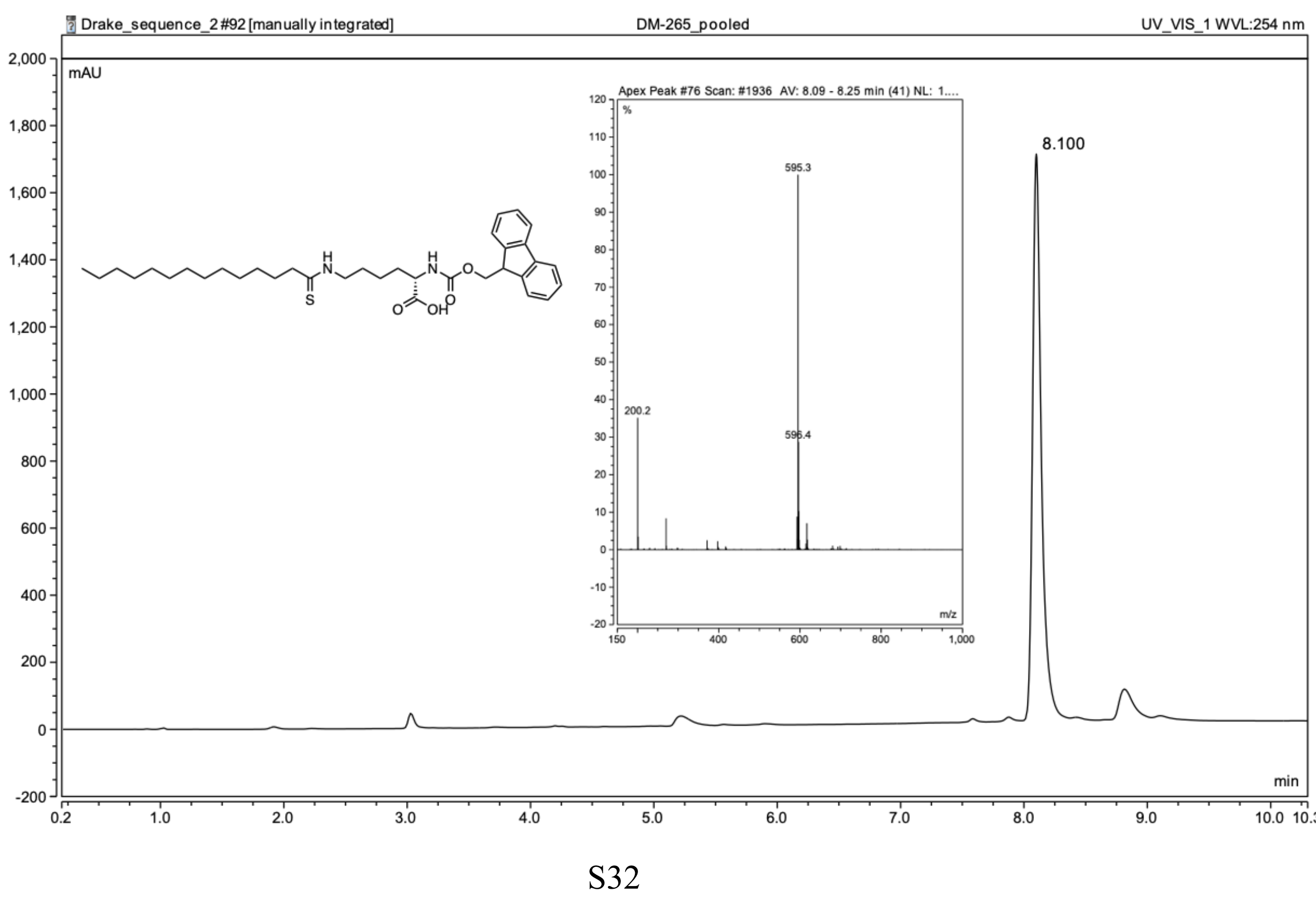

ACSK617 formyl

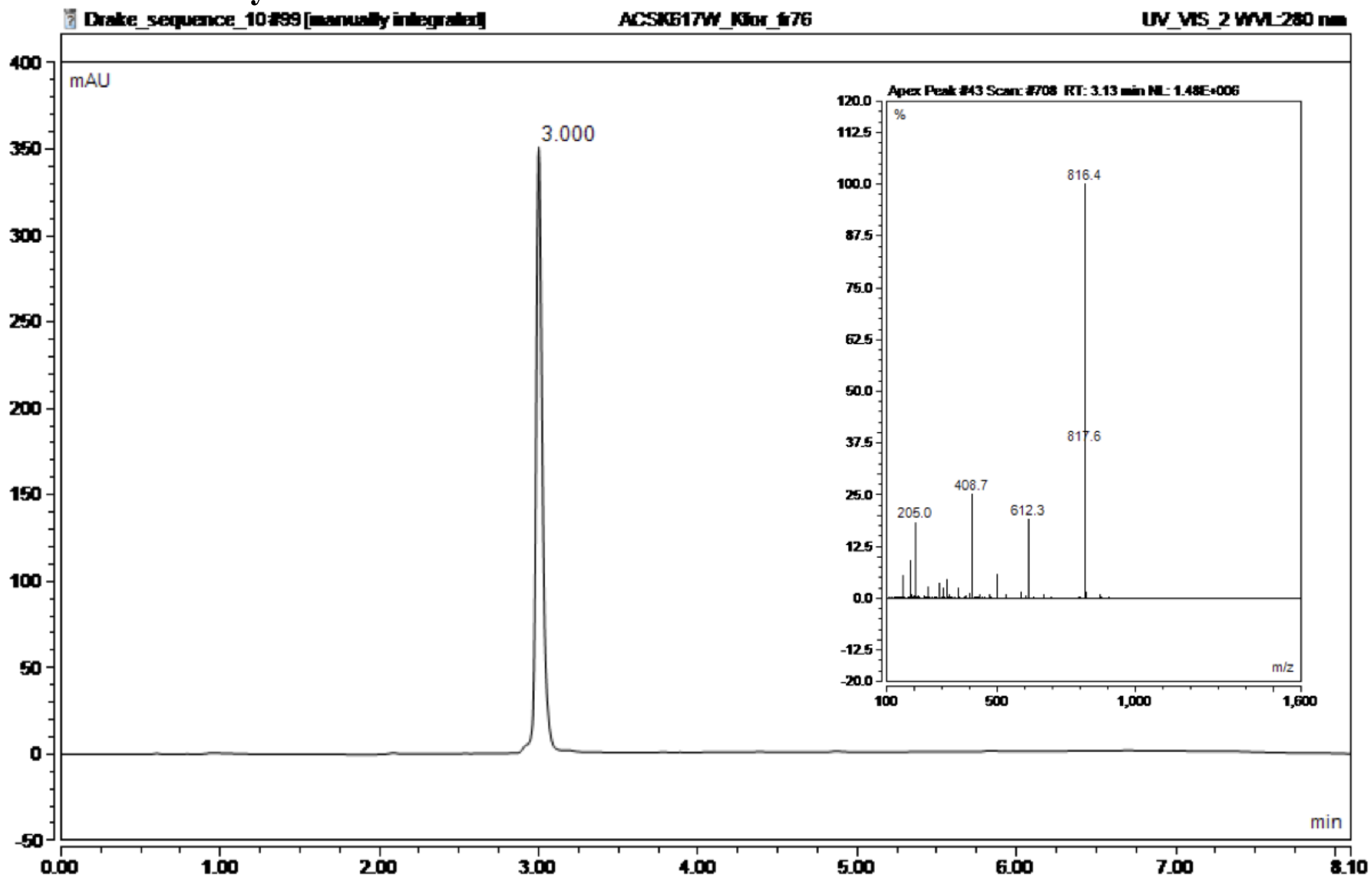

ACSK617 acetyl

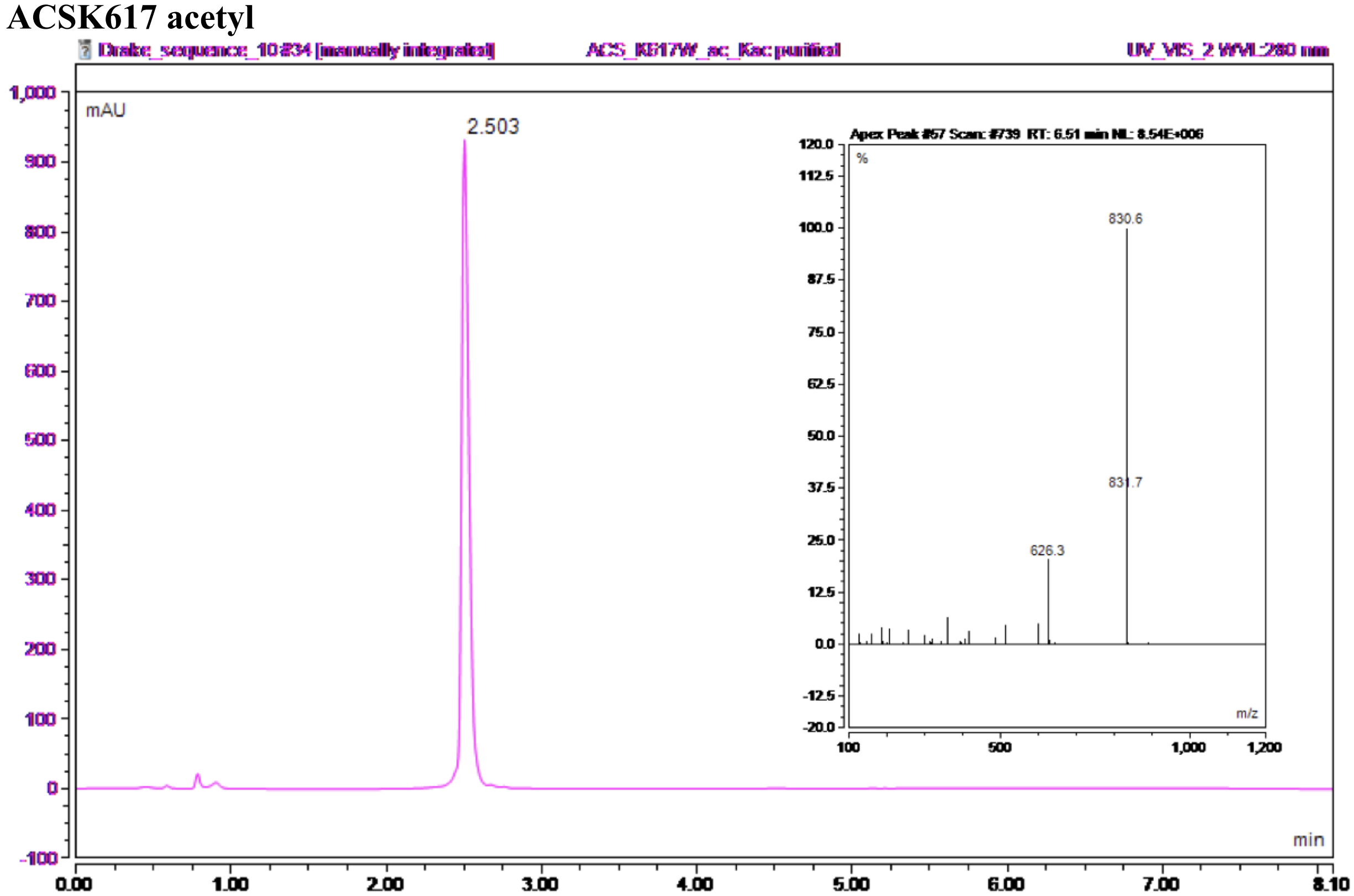

ACSK617 propionyl

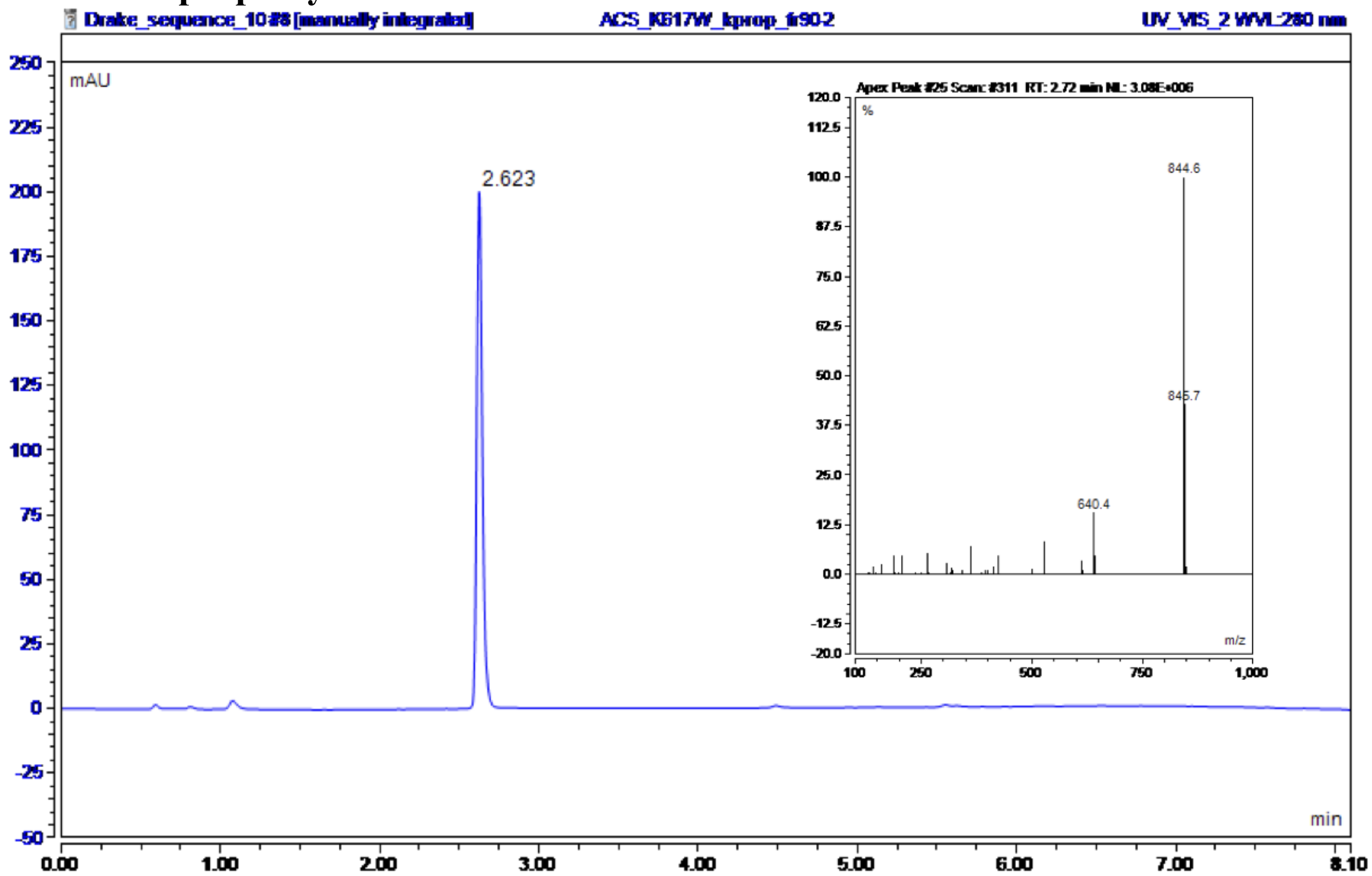

ACSK617 butyryl

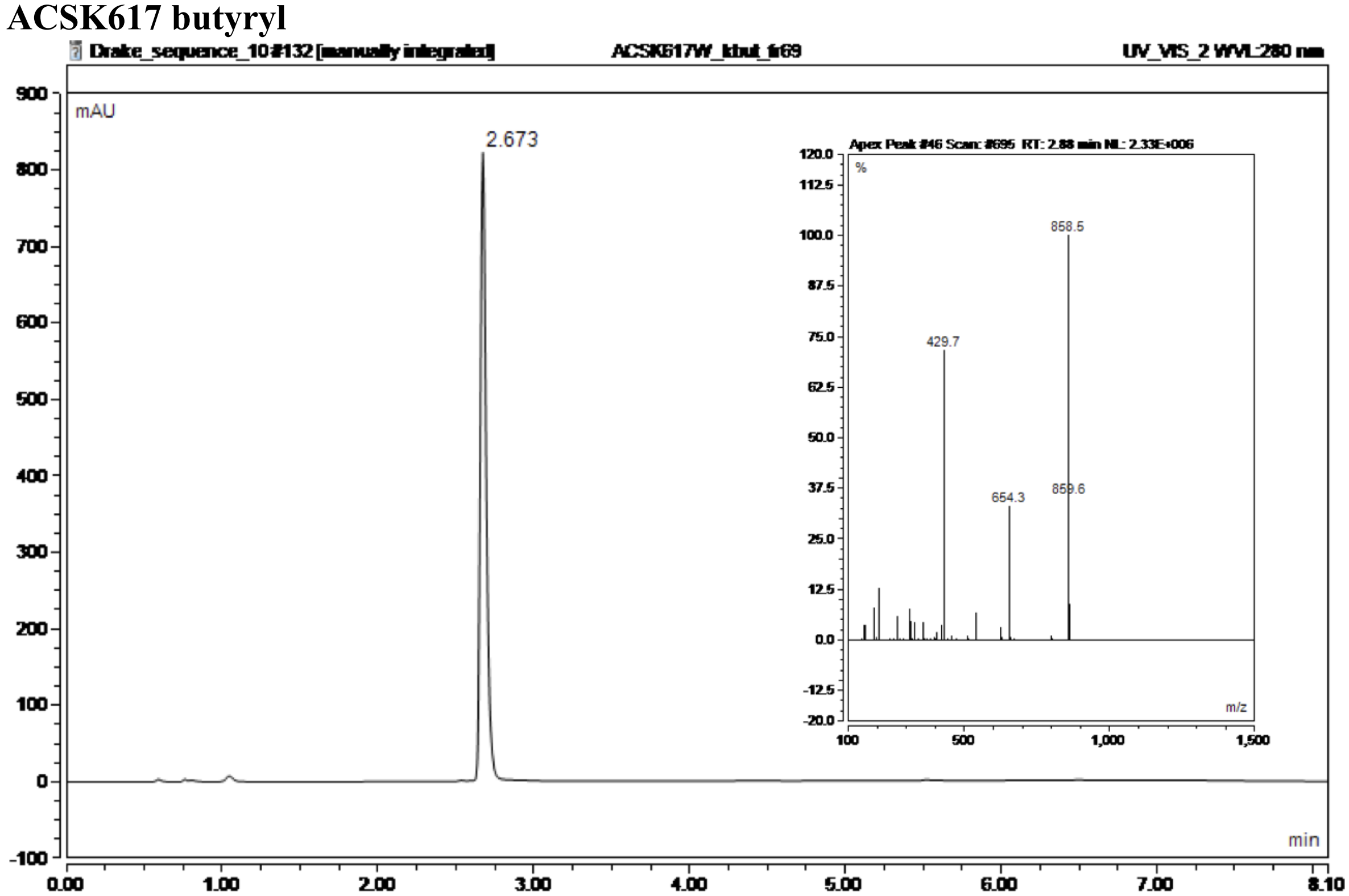

ACSK617 crotonyl

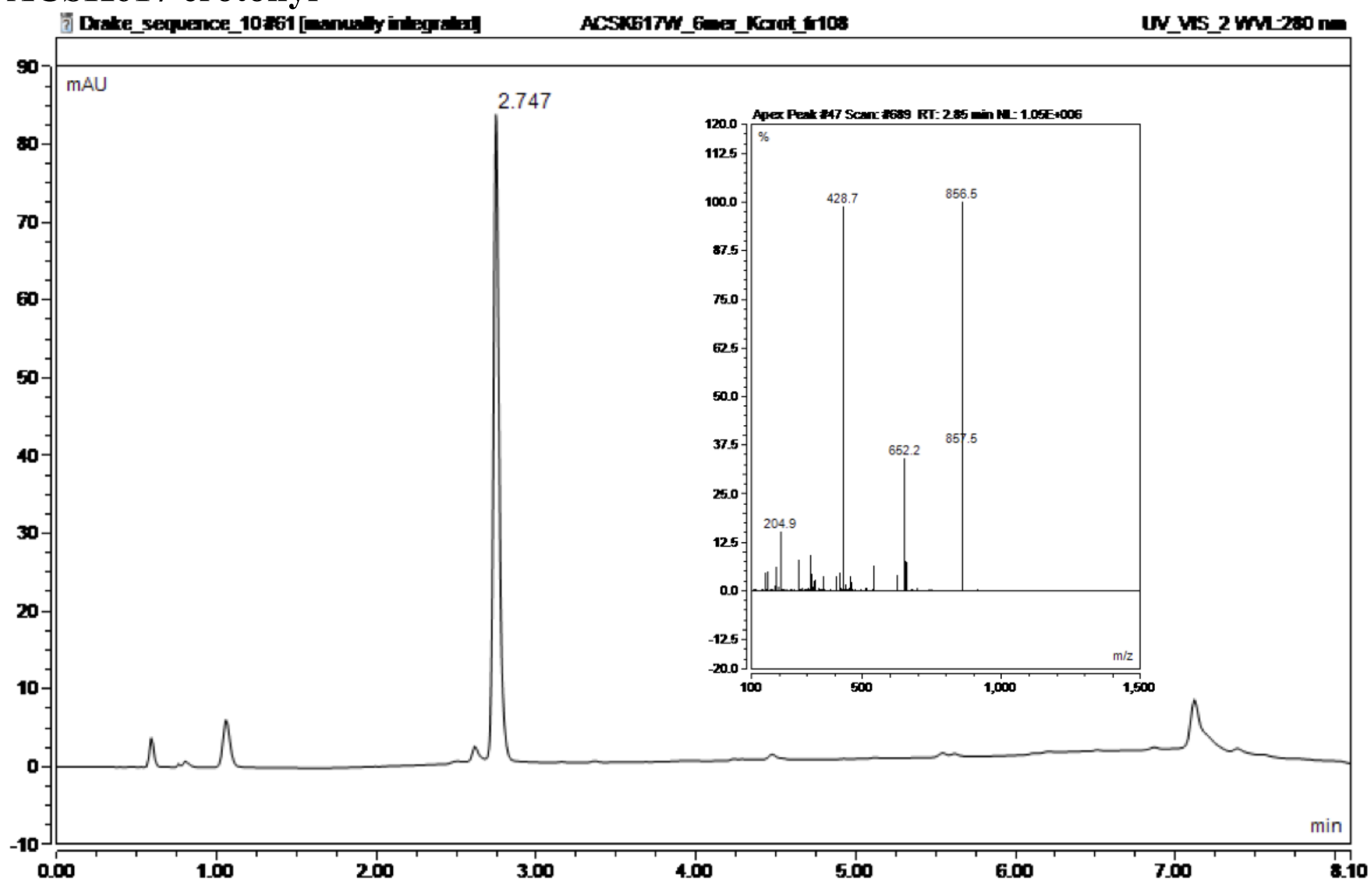

ACSK617 hexanoyl

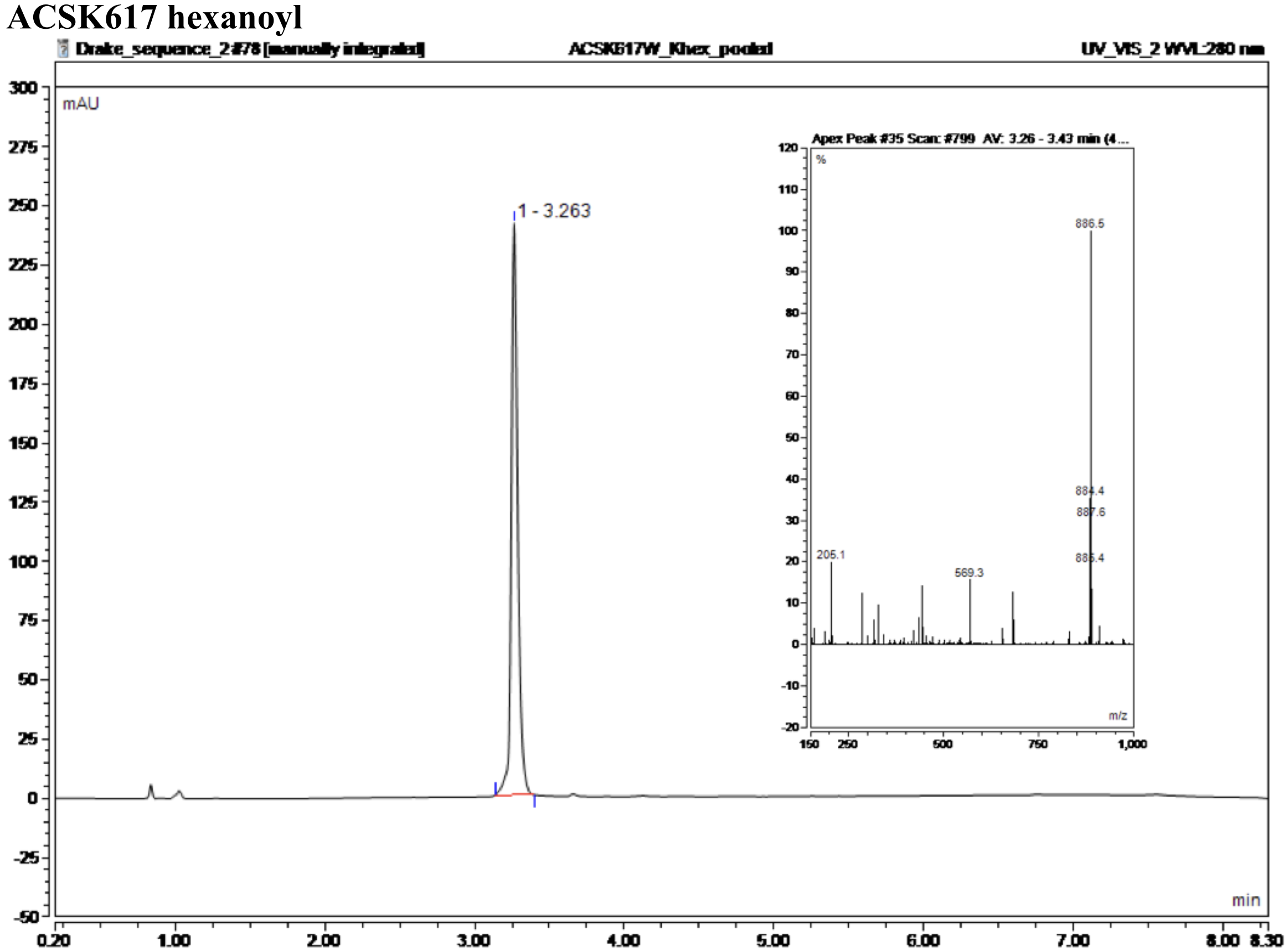

ACSK617 octanoyl

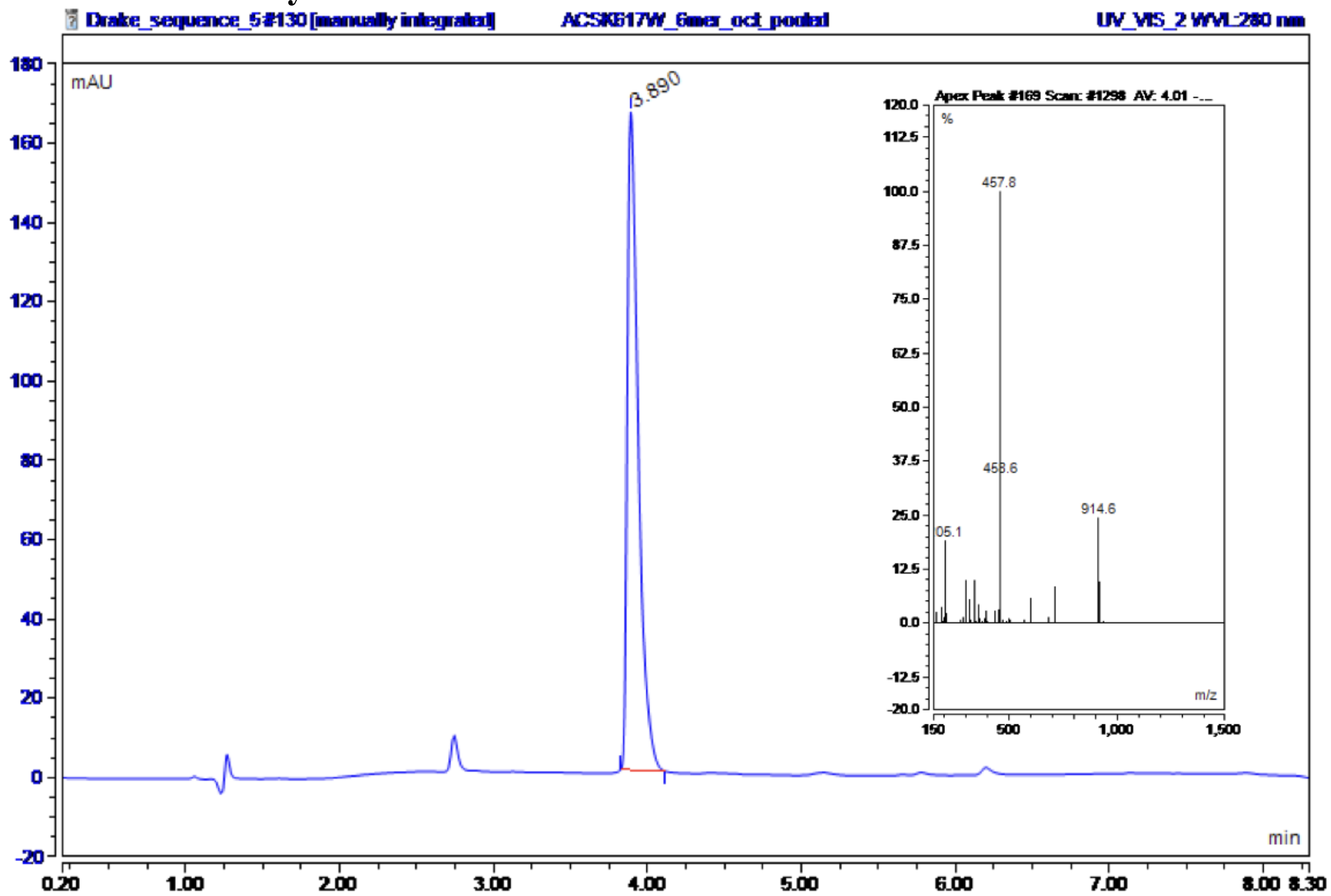

ACSK617 decanoyl

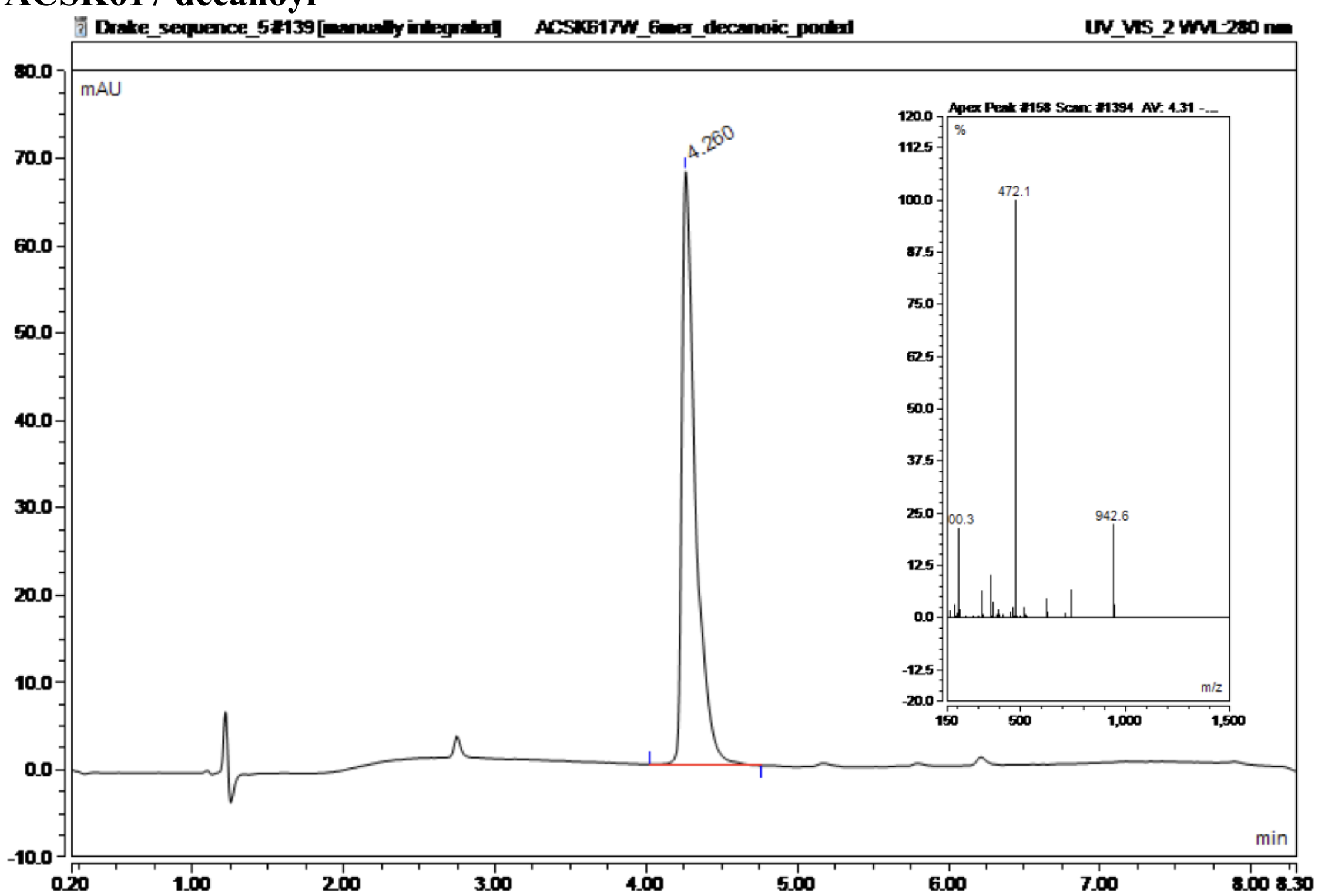

ACSK617 dodecanoyl

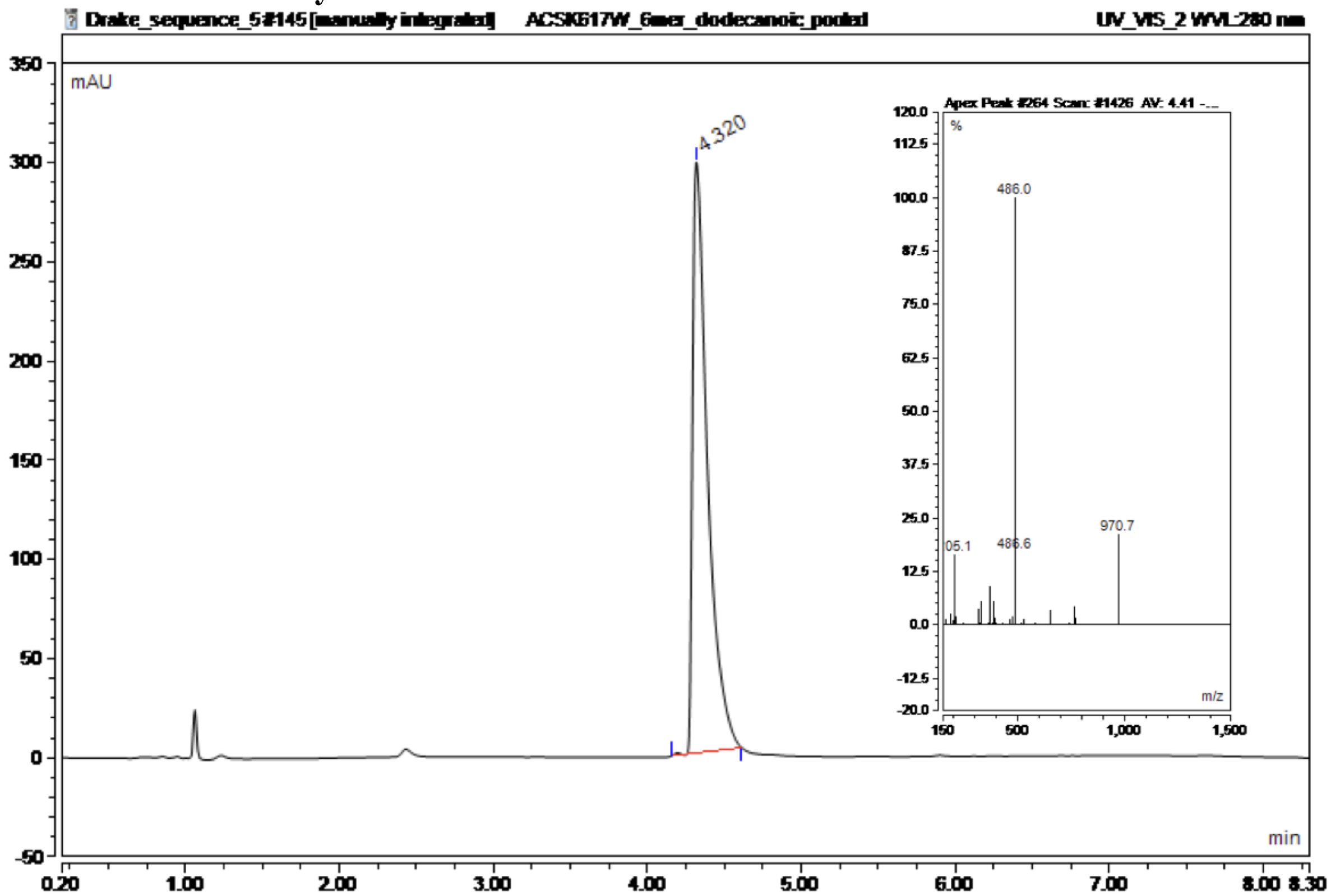

ACSK617 myristoyl

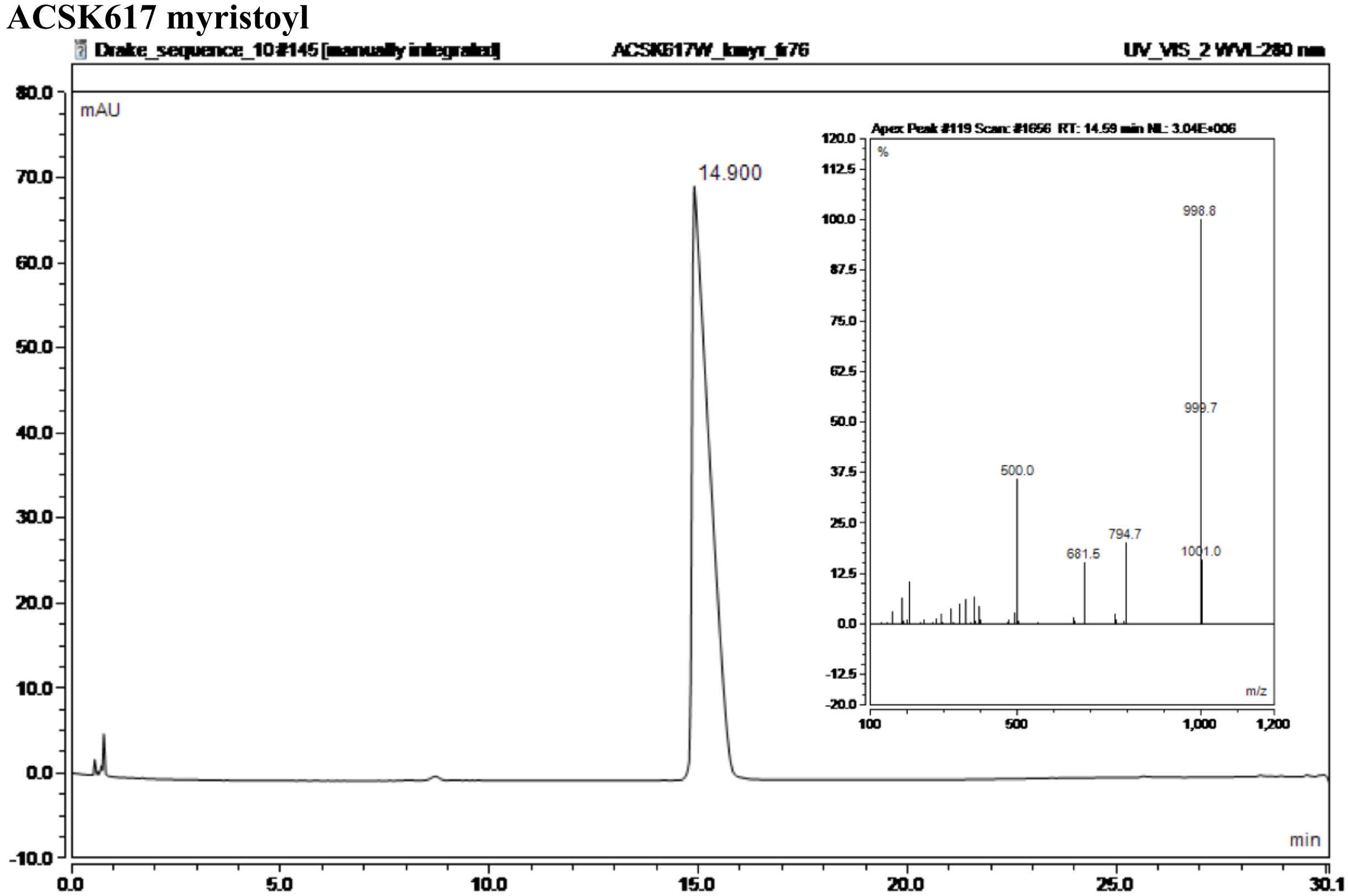

ACSK617 palmitoyl

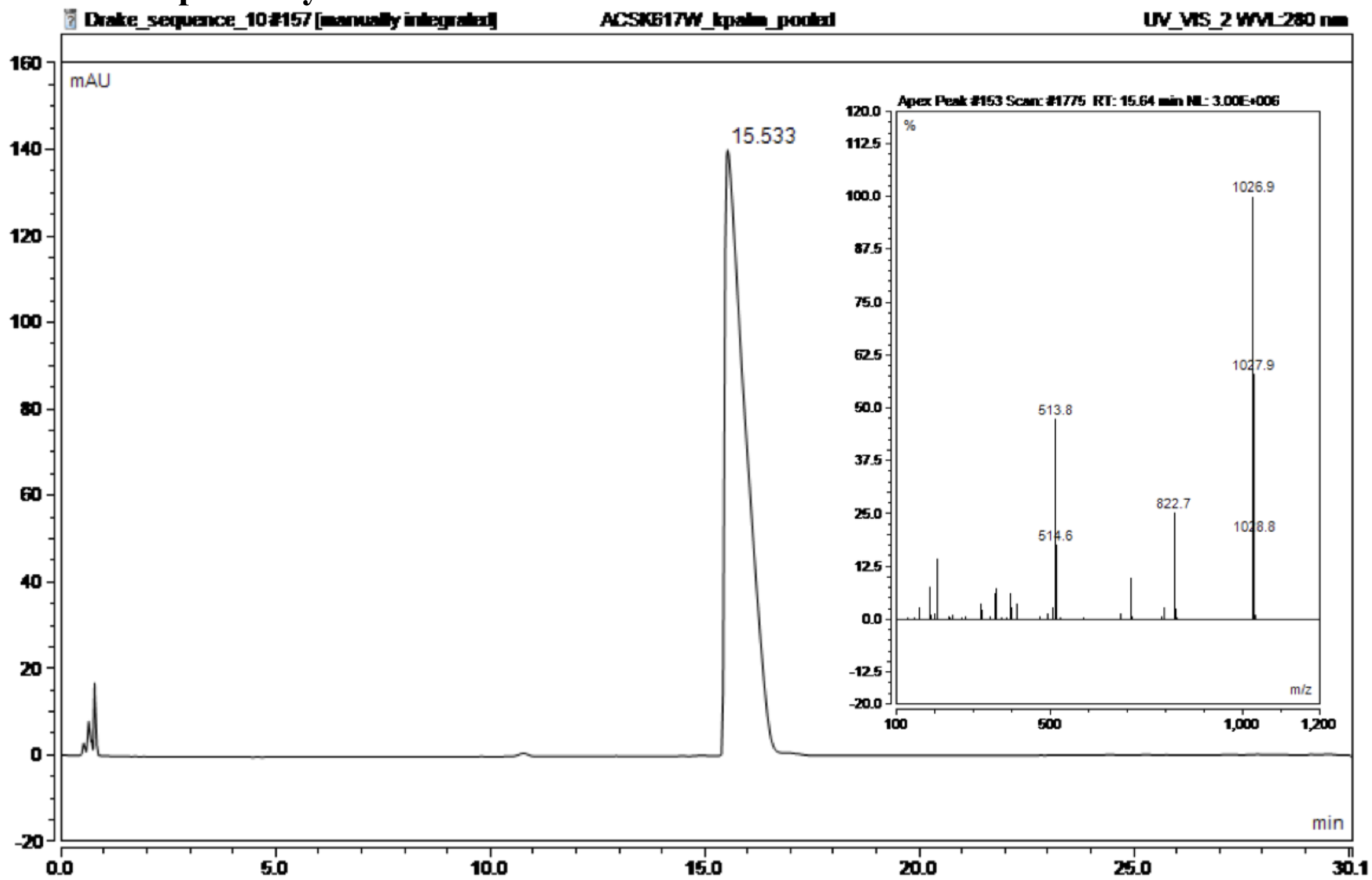

ACSK617 malonyl

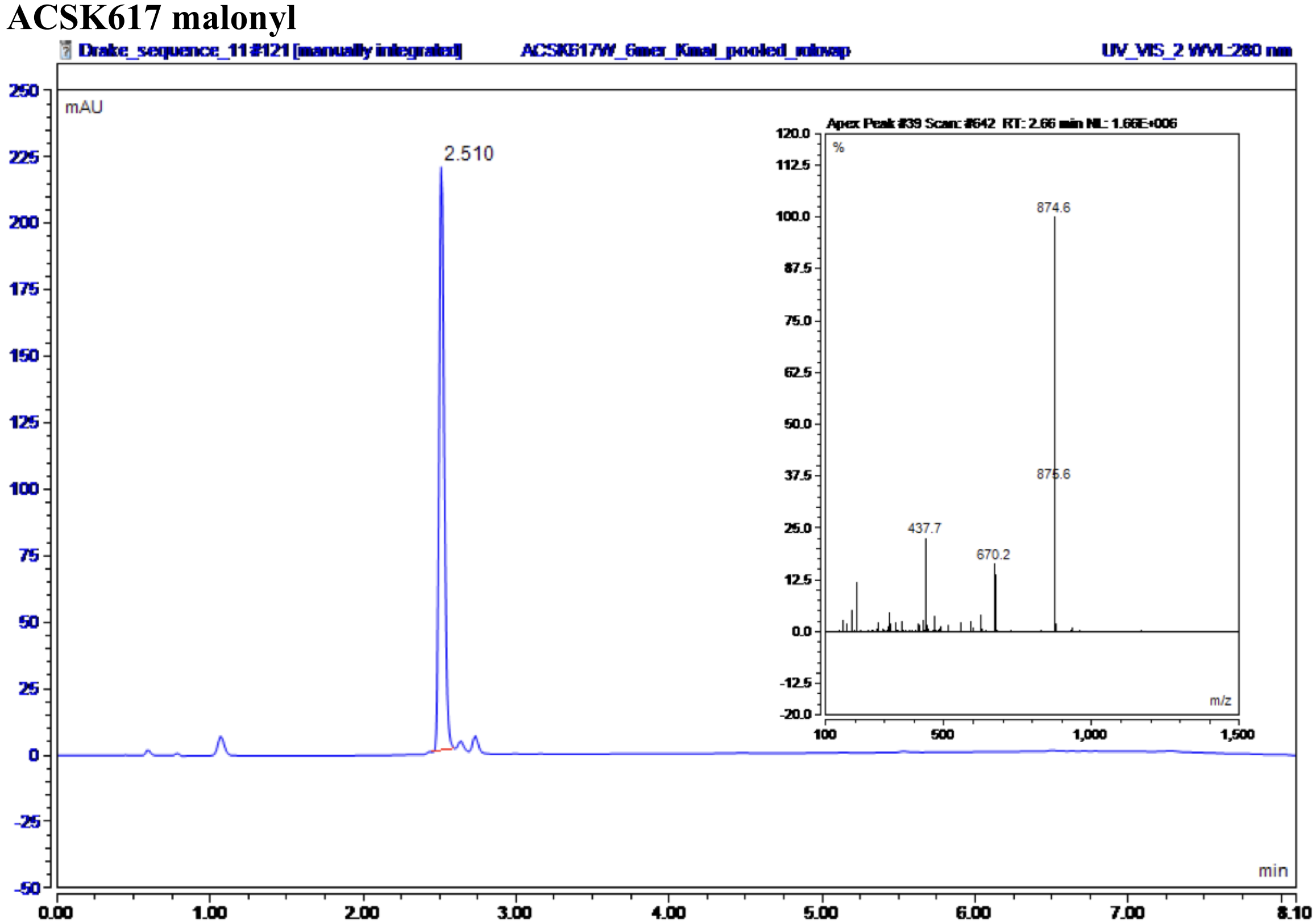

ACSK617 succinyl

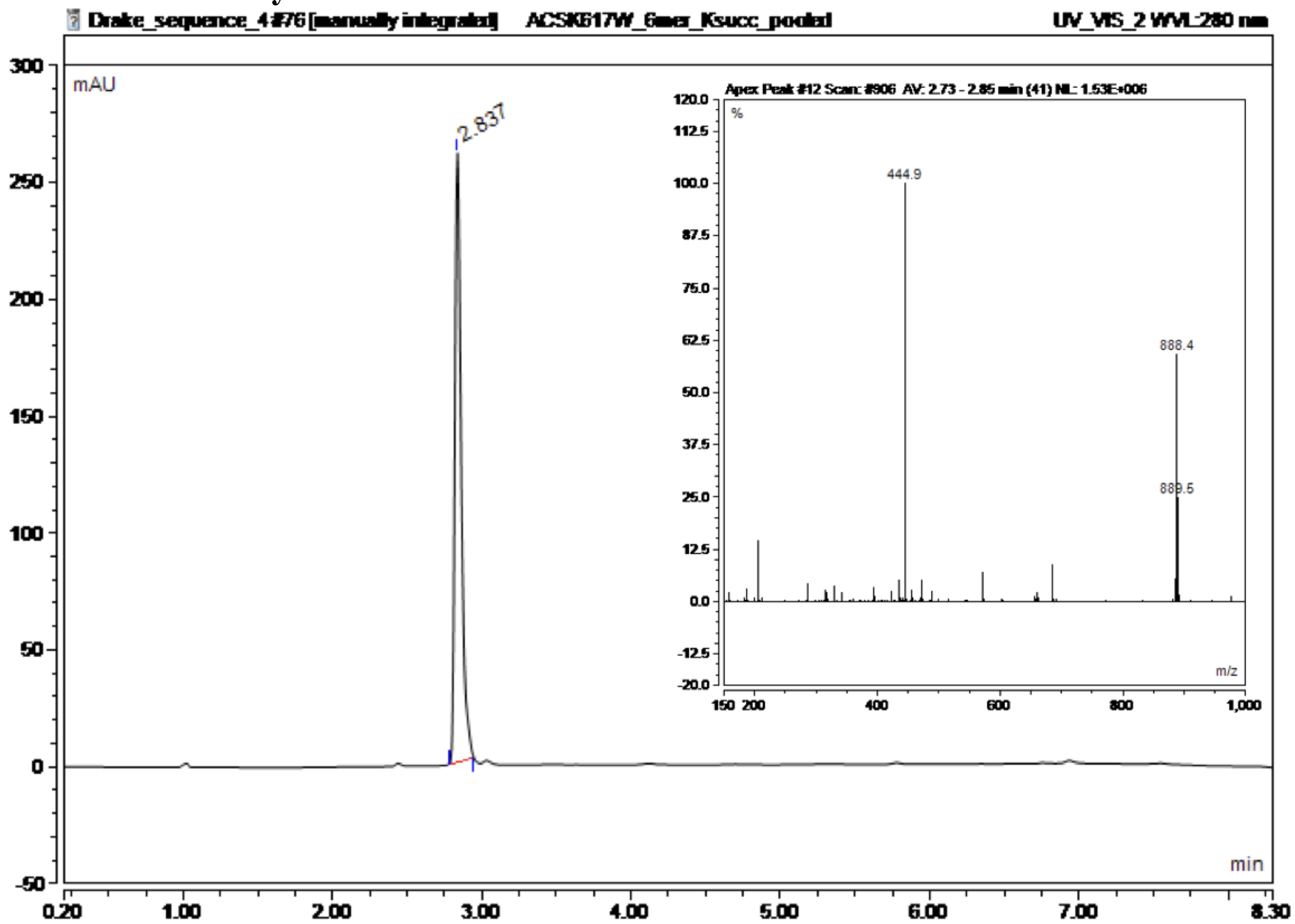

ACSK617 glutaryl

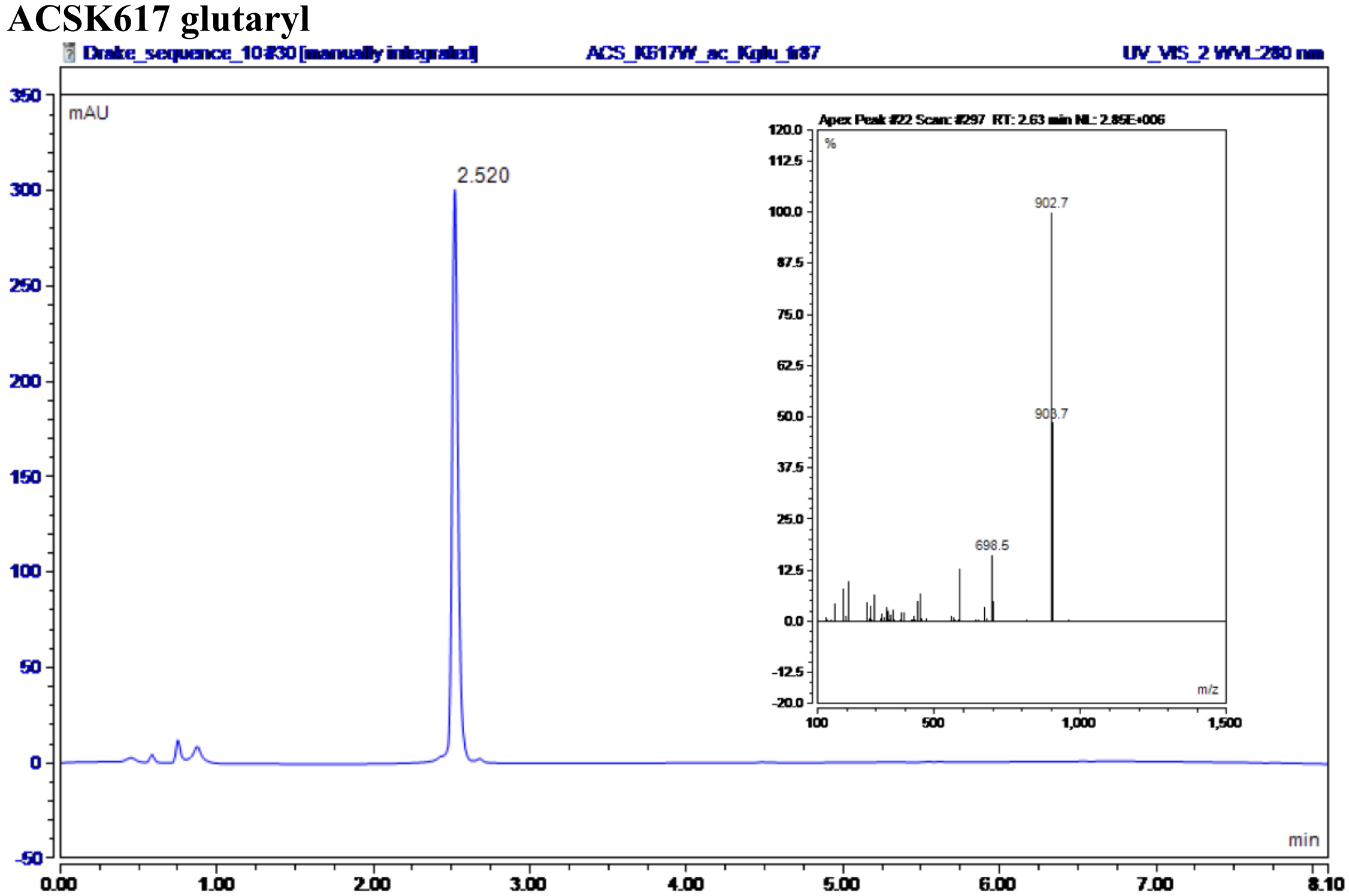

ACSK617 biotinoyl

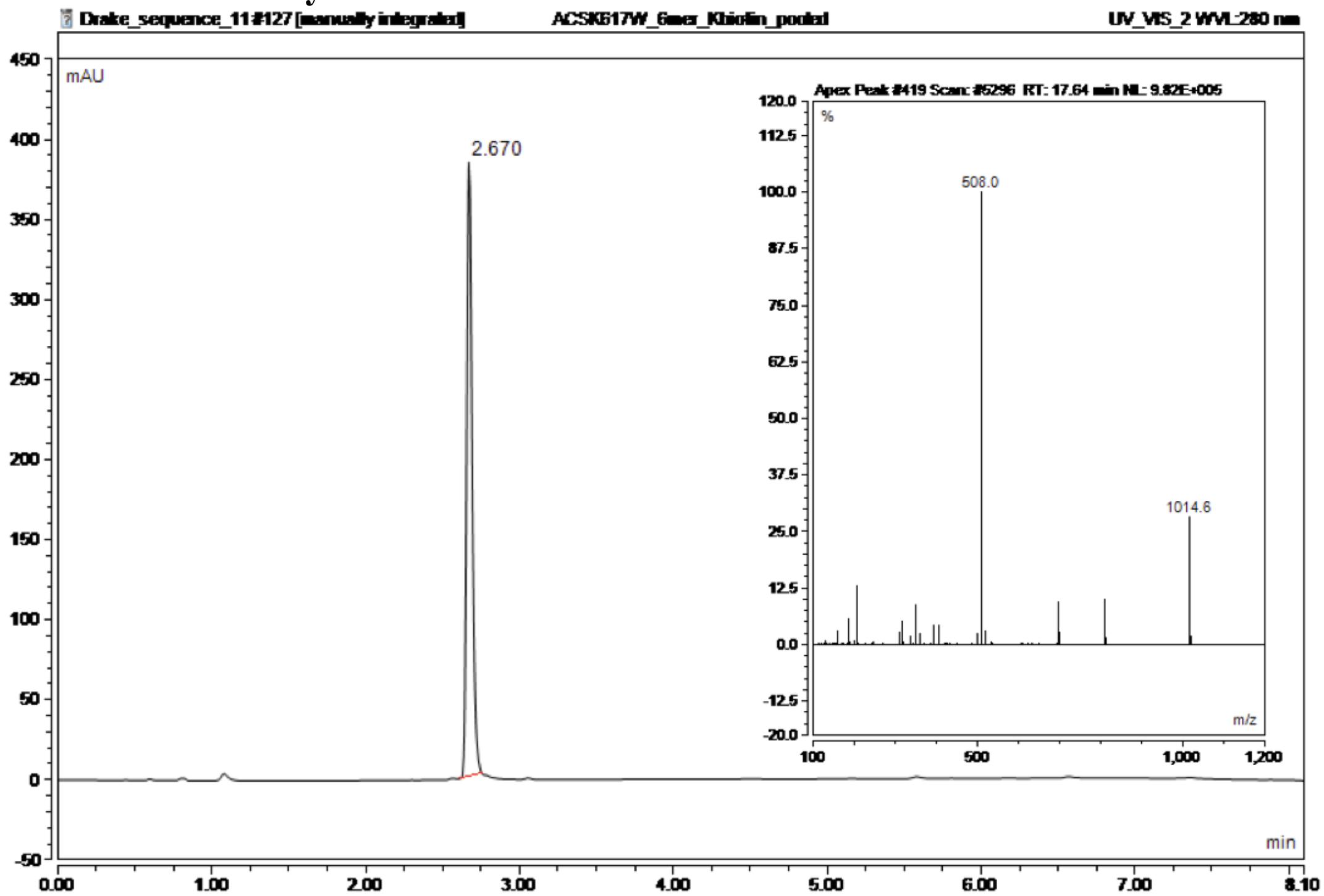

ACSK617 thiomyristol

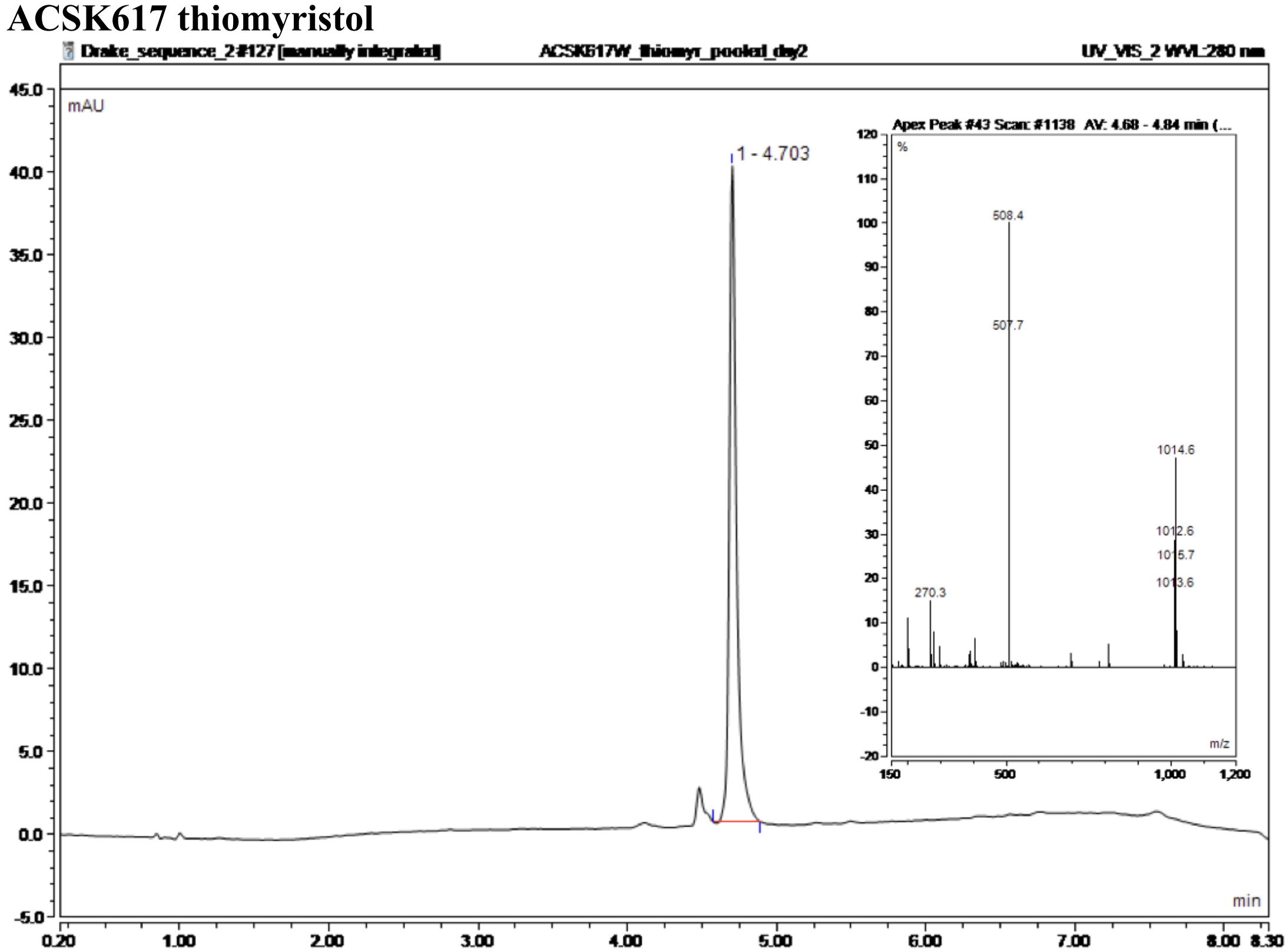

Supplementary Information

NMR Spectra:

(Cbz-Lys(ac)-AMC)

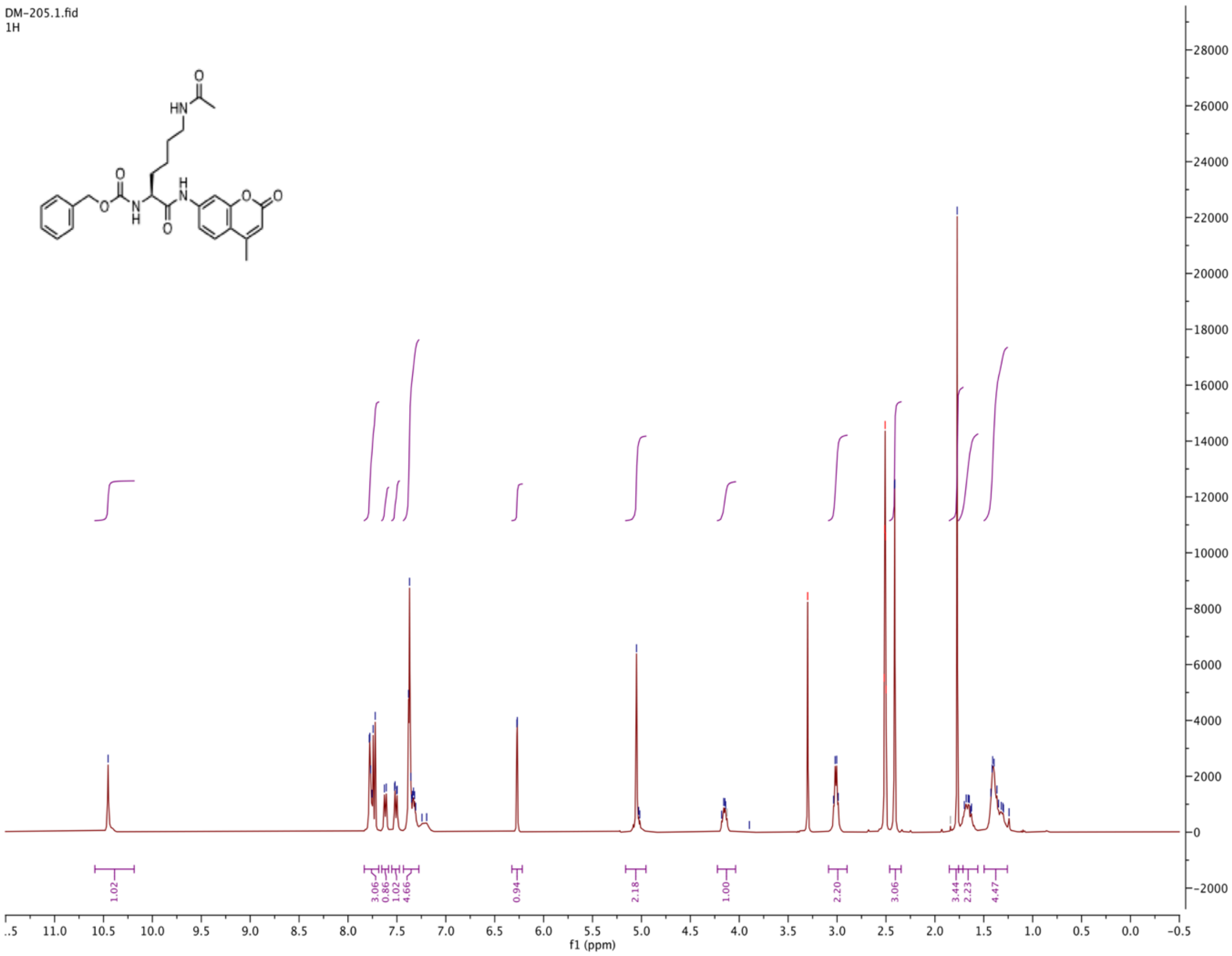

(Cbz-Lys(prop)-AMC)

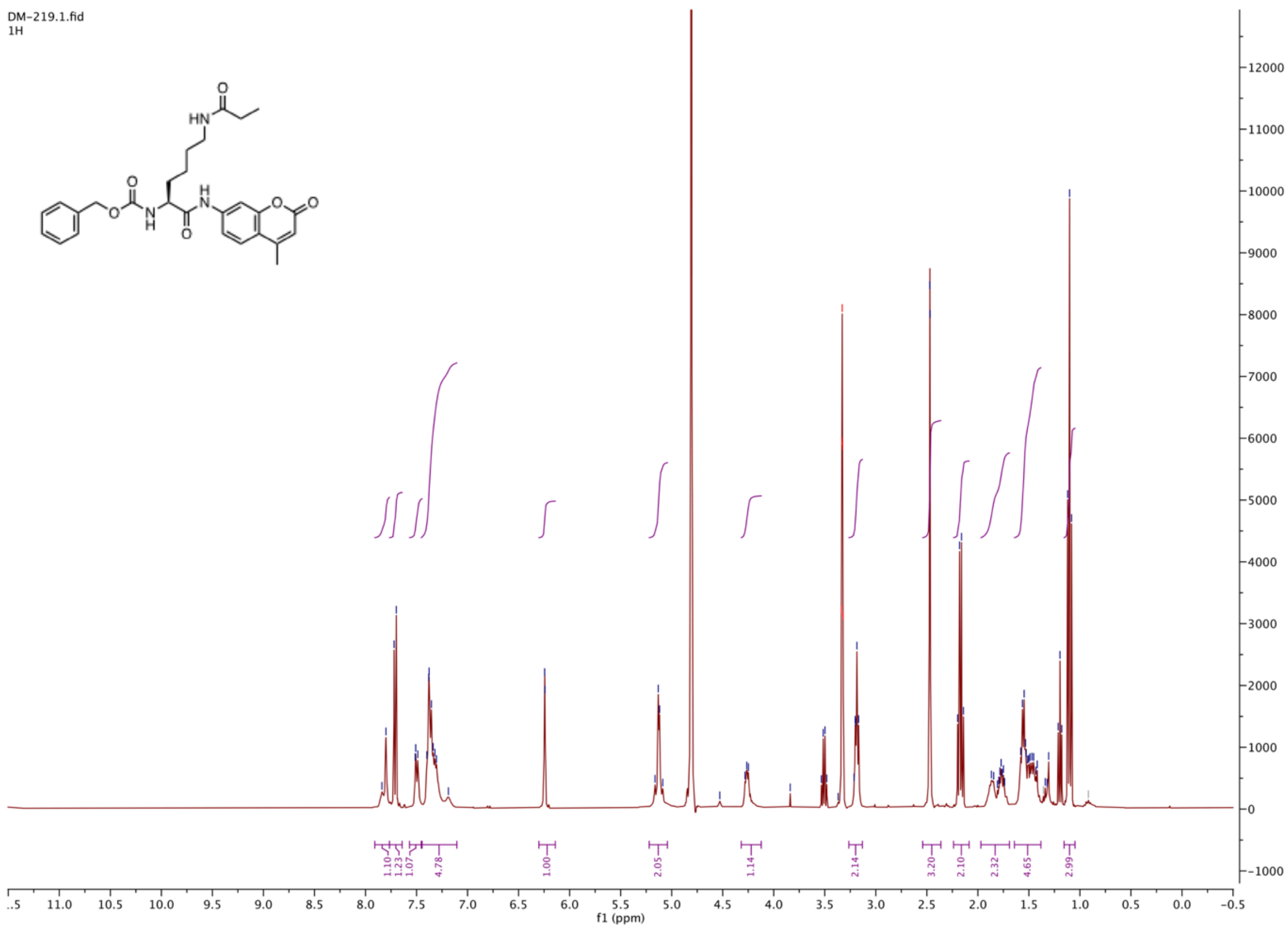

(Cbz-Lys(but)-AMC)

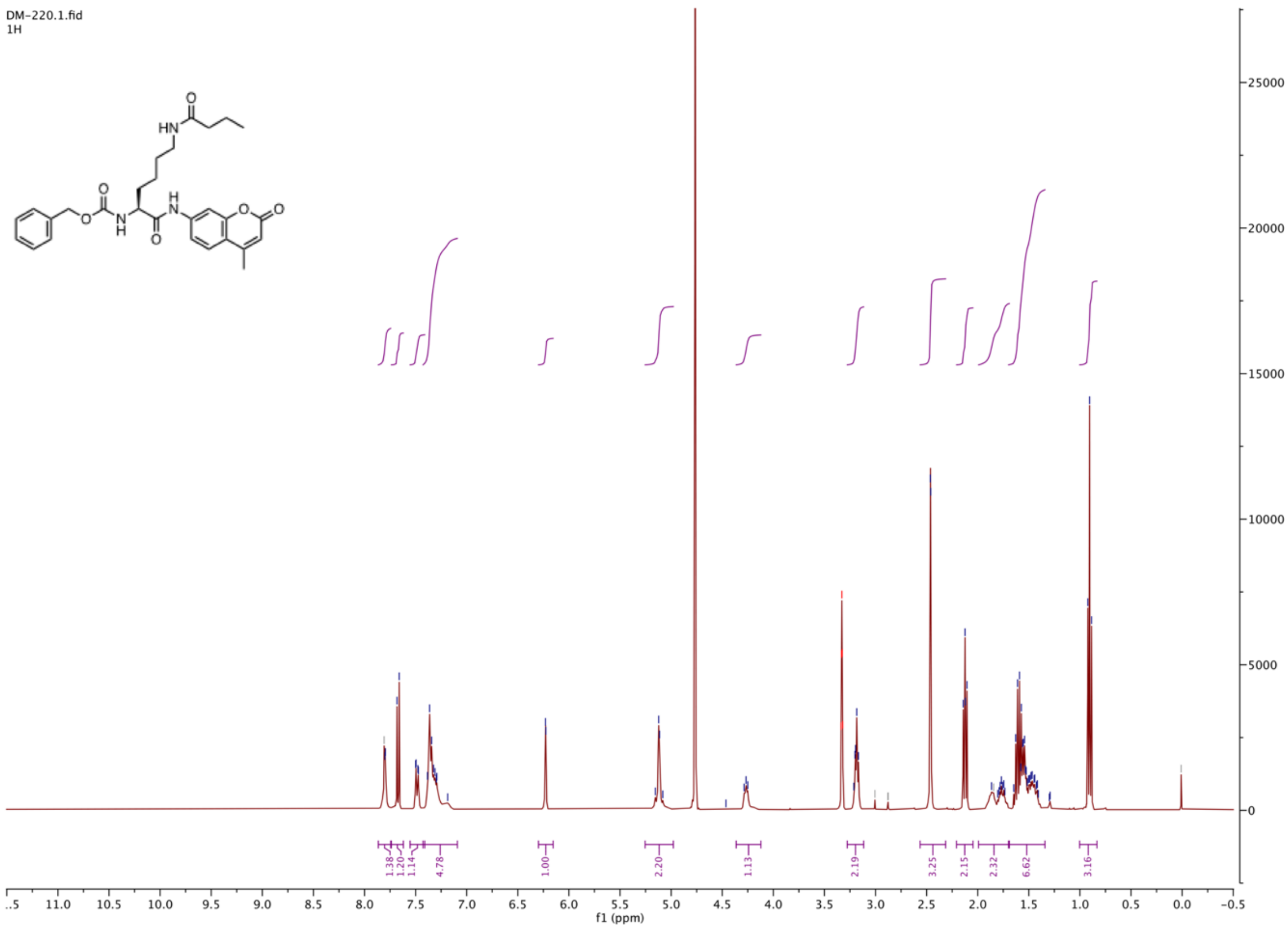

(Cbz-Lys(mal)-AMC)

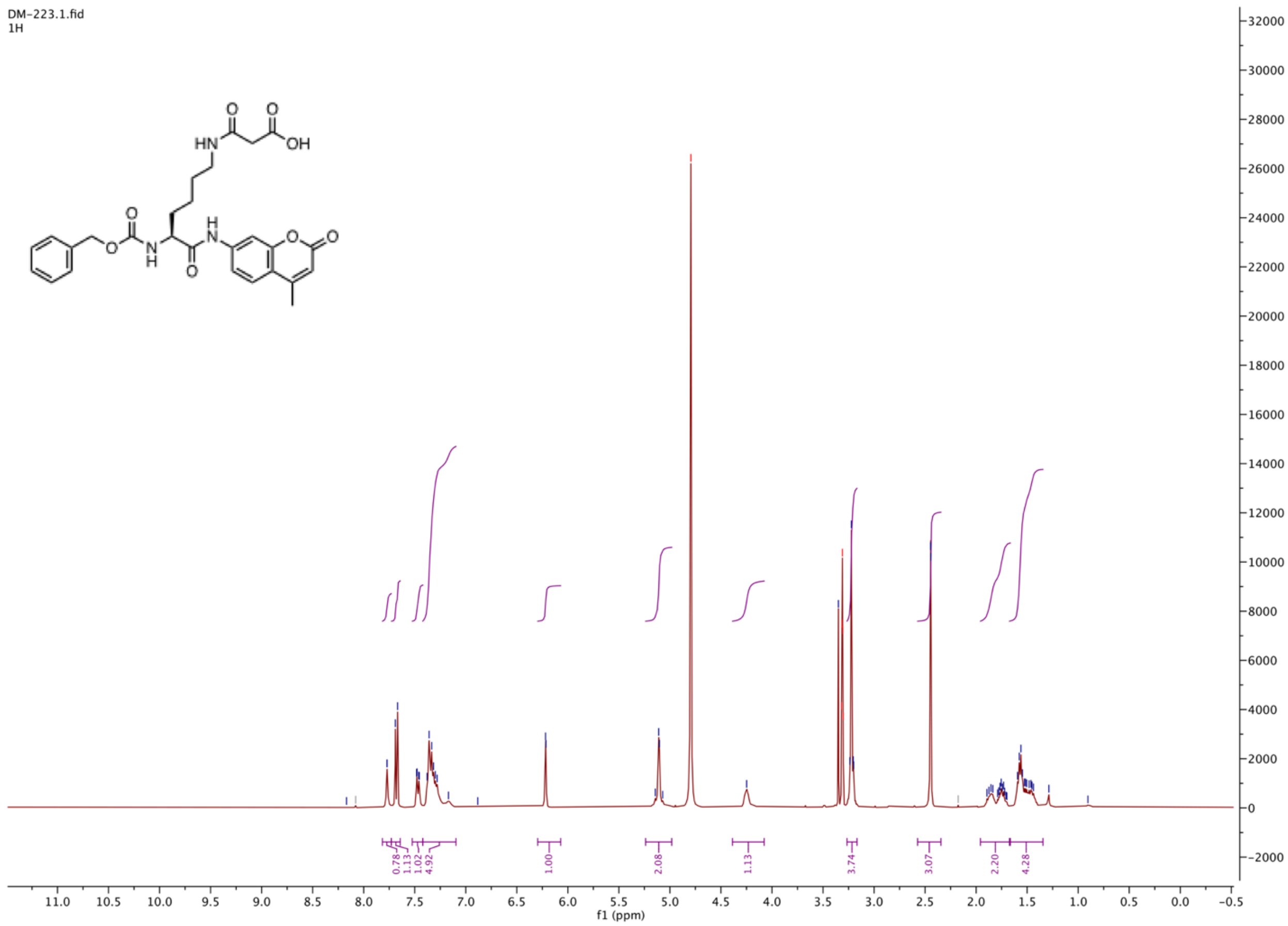

(Cbz-Lys(succ)-AMC)

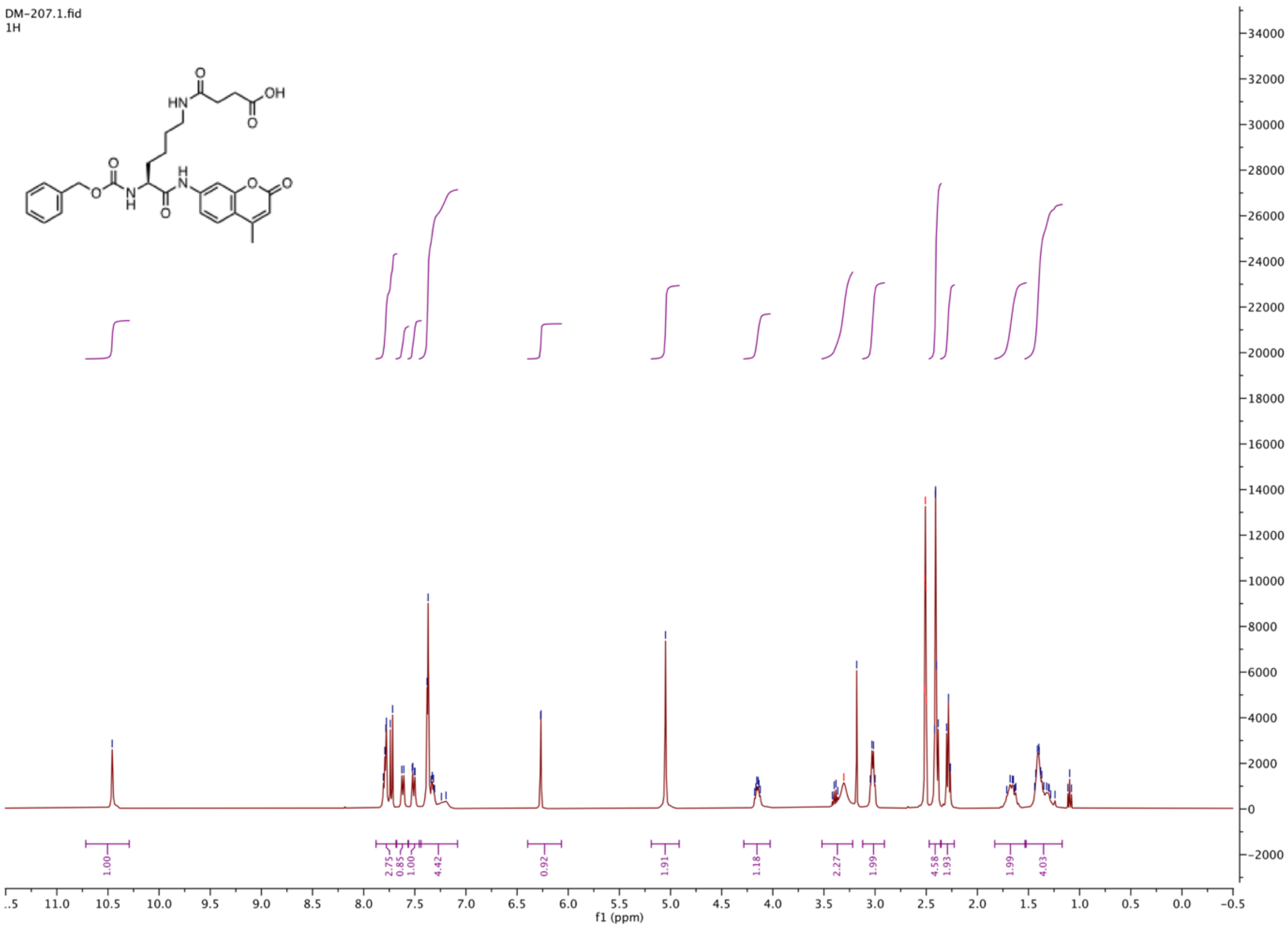

5-(*tert*-butoxy)-5-oxopentanoic

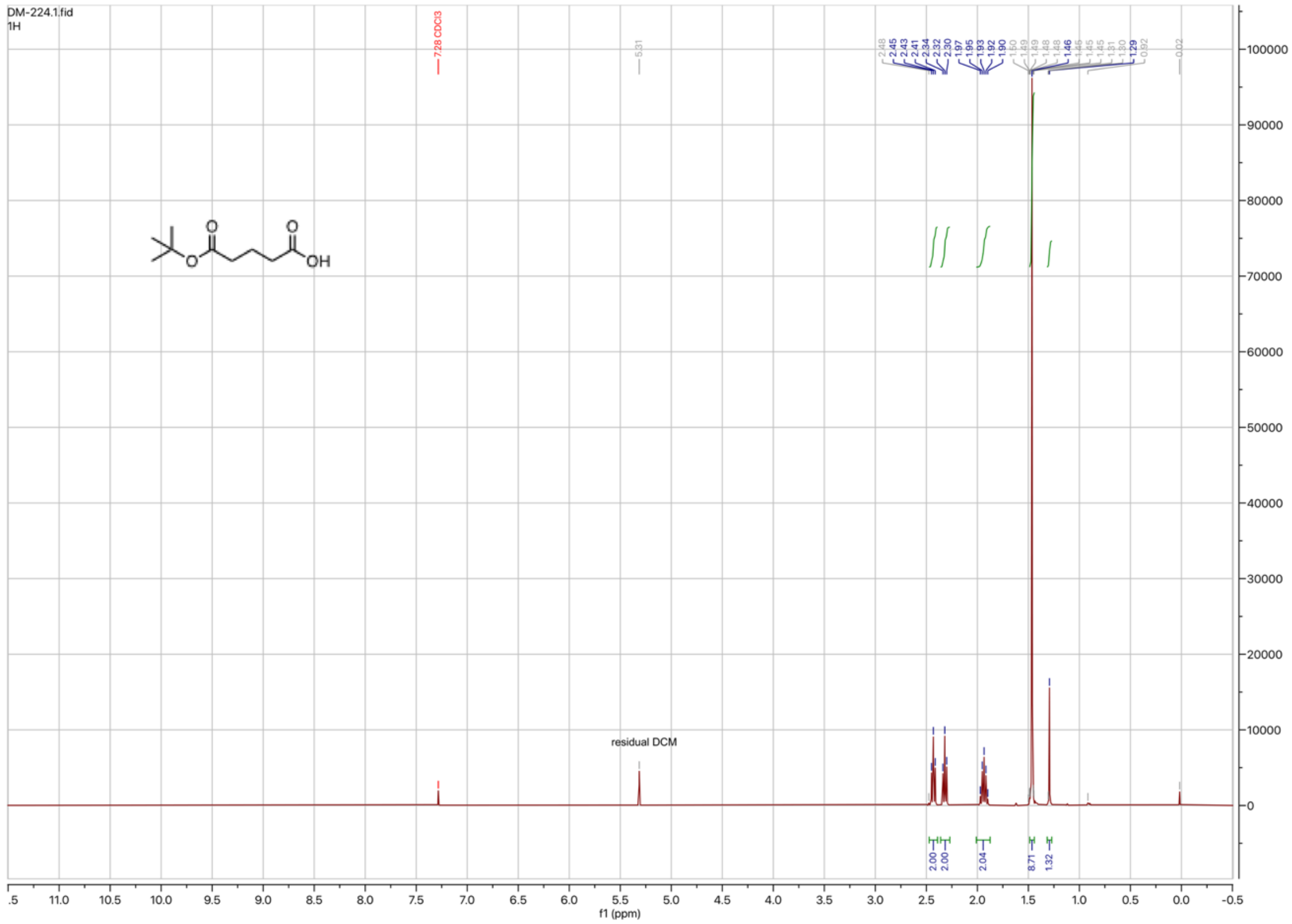

(Cbz-Lys(glut)-AMC)

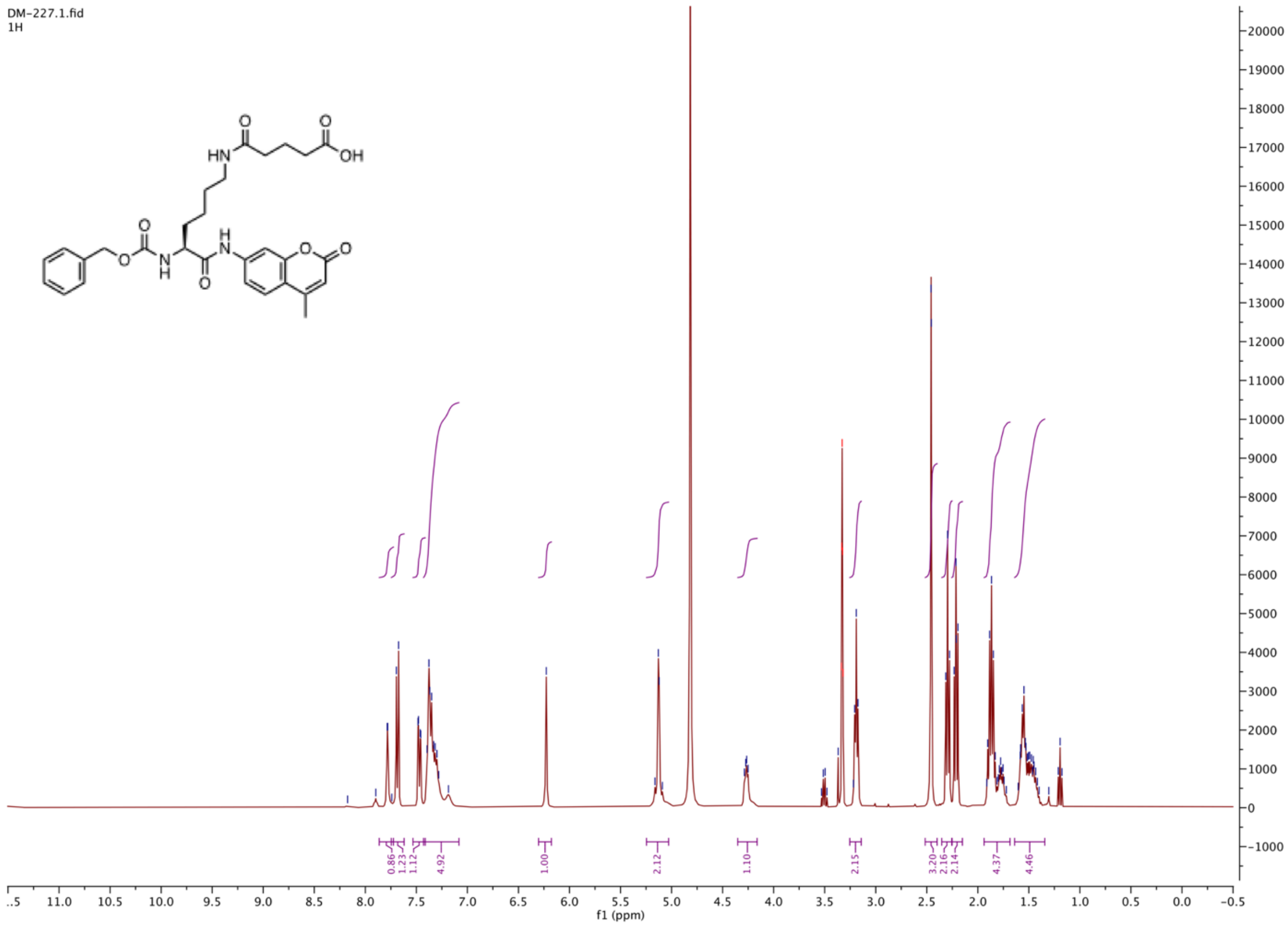

benzyl (*S*)-(6-(3-methylthioureido)-1-oxo-1-(phenylamino)hexan-2-yl)carbamate (1)

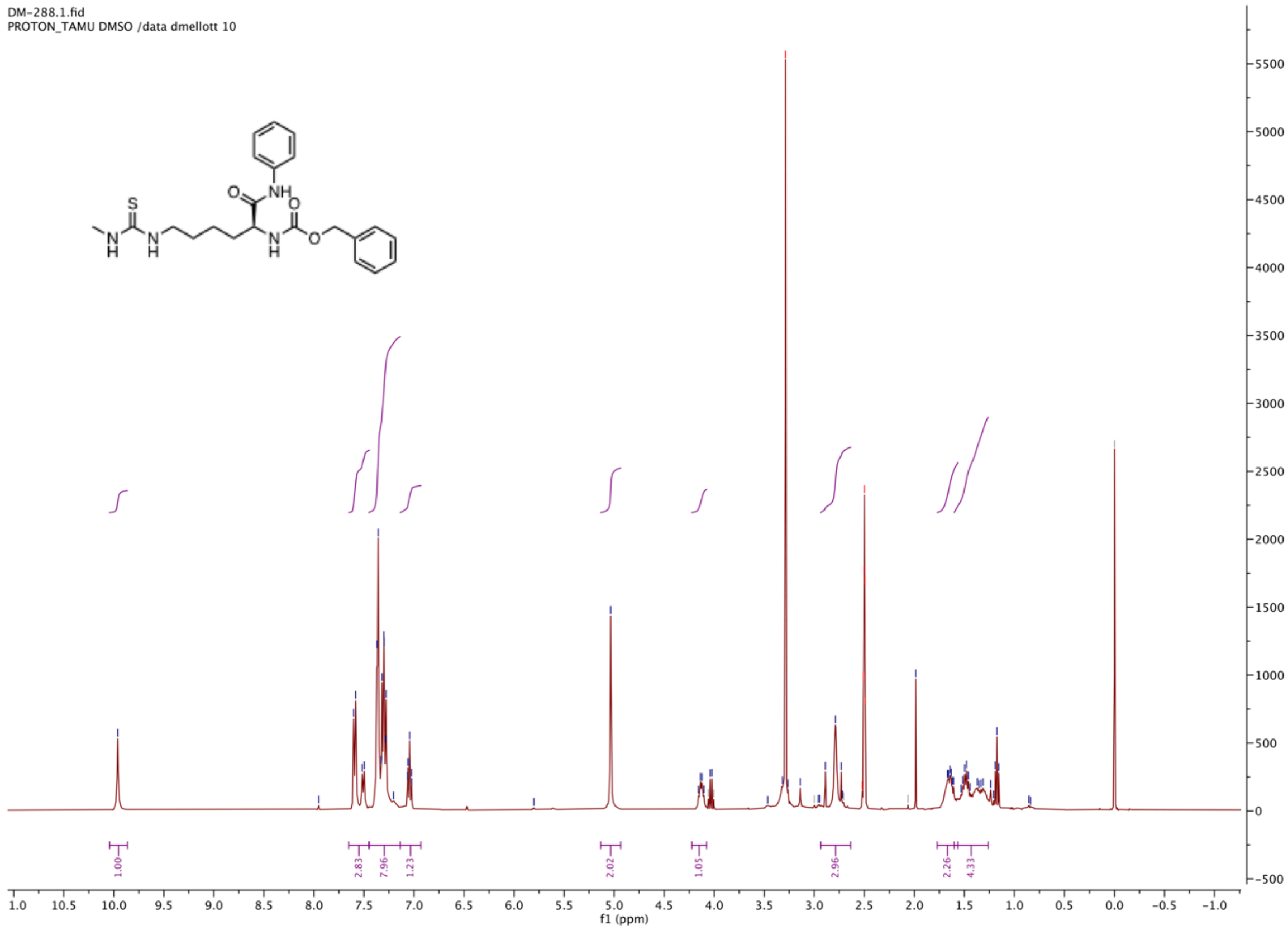

benzyl (S)-(6-(3-dodecylthioureido)-1-oxo-1-(phenylamino)hexan-2-yl)carbamate (2)

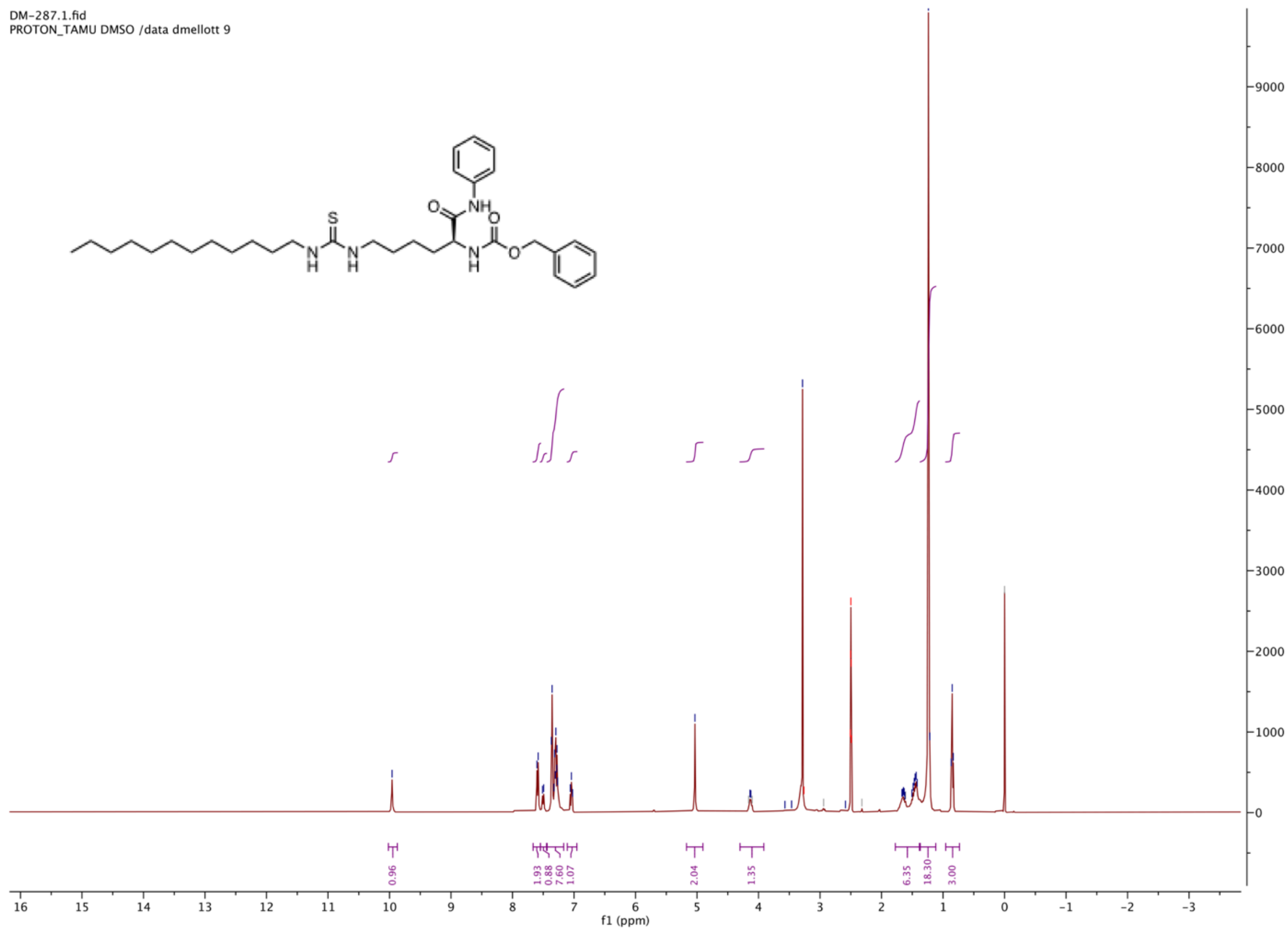

(S)-((5-(((benzyloxy)carbonyl)amino)-6-oxo-6-(phenylamino)hexyl)carbamothioyl)glycine (3)

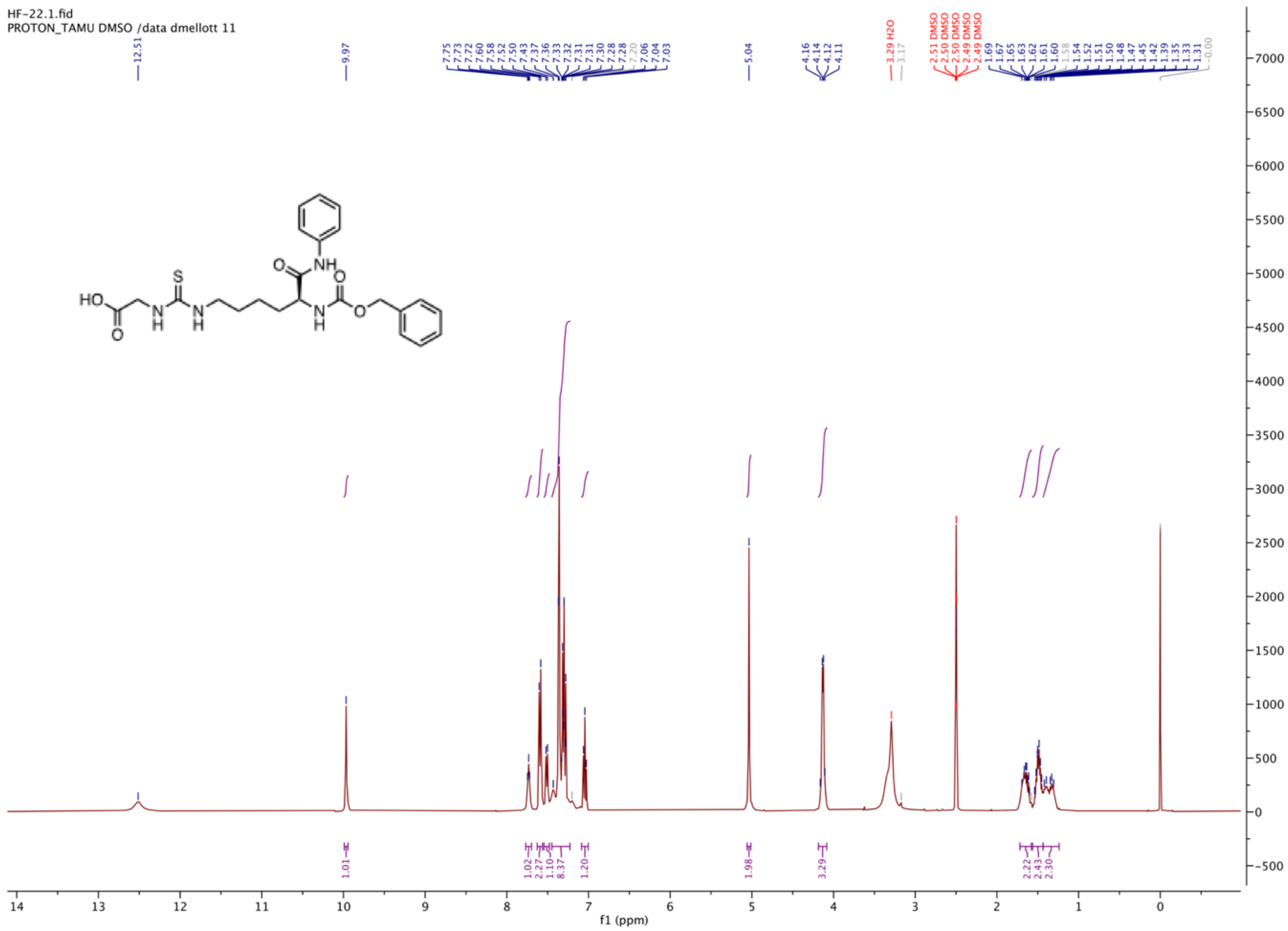

References:

1. Gu, J.; Deng, J.-Y.; Li, R.; Wei, H.; Zhang, Z.; Zhou, Y.; Zhang, Y.; Zhang, X.-E., Cloning and characterization of NAD-dependent protein deacetylase (Rv1151c) from Mycobacterium tuberculosis. *Biochemistry (Moscow)* **2009,** *74* (7), 743-748.
2. Pham, T. V.; Mellott, D. M.; Moghadamchargari, Z.; Chen, K.; Krieger, I.; Laganowsky, A.; Sacchettini, J. C.; Meek, T. D., Covalent Inactivation of Mycobacterium tuberculosis Isocitrate Lyase by cis-2,3-Epoxy-Succinic Acid. *ACS Chemical Biology* **2021,** *16* (3), 463-470.
3. Mellott, D. M.; Torres, D.; Krieger, I. V.; Cameron, S. A.; Moghadamchargari, Z.; Laganowsky, A.; Sacchettini, J. C.; Meek, T. D.; Harris, L. D., Mechanism-Based Inactivation of Mycobacterium tuberculosis Isocitrate Lyase 1 by (2R,3S)-2-Hydroxy-3-(nitromethyl)succinic acid. *Journal of the American Chemical Society* **2021,** *143* (42), 17666-17676.
4. Meany, J. E.; Pocker, Y., The dehydration of glyoxalate hydrate: general-acid, general-base, metal ion and enzymic catalysis. *Journal of the American Chemical Society* **1991,** *113* (16), 6155-6161.
5. Heltweg, B.; Dequiedt, F.; Marshall, B. L.; Brauch, C.; Yoshida, M.; Nishino, N.; Verdin, E.; Jung, M., Subtype Selective Substrates for Histone Deacetylases. *Journal of Medicinal Chemistry* **2004,** *47* (21), 5235-5243.
6. Xu, J.; Ma, Y.; Xu, S.; Zhao, Y., Negative-ion electrospray ionization mass spectrometry of N-benzyloxycarbonyl-protected 1-substituted and cyclic taurines. *Rapid Communications in Mass Spectrometry* **2005,** *19* (13), 1835-1838.
